# Supplementary material for: Heteroaryl derivatives for hole-transport layers improve thermal stability of perovskite solar cells
Source: Nat Commun. 2026 Feb 13;17:1664. doi: 10.1038/s41467-025-68236-9 (PMC12909905; doi:10.1038/s41467-025-68236-9)
Supplement: Supplementary file 1 — Supplementary Information [file 41467_2025_68236_MOESM1_ESM.pdf]

Supplementary information

Heteroaryl Derivatives for Hole-Transport Layers Improve Thermal Stability of Perovskite Solar Cells

Hiroyuki Kanda[1*], Santa Mondal[1], Naoto Eguchi[1], Naoyuki Nishimura[1], Yoyo Hinuma[1], Kohei Yamamoto[1], Masaki Yumoto[1], Kenichi Tashiro[1], Hideyuki Takada[1], Aiko Narazaki[1], Takashi Koida[1], and Takurou N. Murakami[1]

[1]National Institute of Advanced Industrial Science and Technology (AIST), Tsukuba, Ibaraki 305-8565, Japan.

Corresponding author: Hiroyuki Kanda, email: hiroyuki.kanda@aist.go.jp

Supplementary Table 1. Code and chemical name of novel heteroaryl additives for Spiro-OMeTAD.

Code	Name	Code	Name
tBP	4-*tert*-Butylpyridine (reference)	K19	4-[4-(4,4,5,5-Tetramethyl-1,3,2-dioxaborolan-2-yl)phenyl]pyridine
K1	4-Ethylpyridine	K20	4-[3-(4,4,5,5-Tetramethyl-1,3,2-dioxaborolan-2-yl)phenyl]pyridine
K2	4-Propylpyridine	K21	4-Benzylpyridine
K3	4-Vinylpyridine	K22	4-Benzoylpyridine
K4	4-Ethynylpyridine	K23	4-Styrylpyridine
K5	4-(5-Nonyl)pyridine	K24	4-(3-Phenylpropyl)pyridine
K6	4-Aminopyridine	K25	4-(4-Pyridyl)morpholine
K7	4-Acetylpyridine	K26	4-(4-Piperidyl)pyridine
K8	Methyl isonicotinate	K27	4-Phenylpyridine
K9	*N*,*N*-Diethylisonicotinamide	K28	3-Phenylpyridine
K10	Allyl isonicotinate	K29	2-Phenylpyridine
K11	2,6-Di-*tert*-butylpyridine	K30	2-(*p*-Tolyl)pyridine
K12	2,6-Diethynylpyridine	K31	3-Phenylthiophene
K13	2,6-Dimethoxypyridine	K32	1-Phenylimidazole
K14	2,4,6-Trimethylpyridine	K33	2,2'-Bipyridyl
K15	2-Amino-4-(trifluoromethyl)pyridine	K34	2,6-Di(1-pyrazolyl)pyridine
K16	3,5-Lutidine	K35	Lepidine
K17	Pentafluoropyridine	K36	5,6,7,8-Tetrahydroisoquinoline
K18	4-(4-Pyridyl)benzaldehyde		

Supplementary Table 2. Stability result of the perovskite solar cells with Li+tBP (reference). Sample was stored at 85 °C in dark.

Time (h)	Jsc (mA/sq) Forward	Jsc (mA/sq) Reverse	Voc (V) Forward	Voc (V) Reverse	FF (-) Forward	FF (-) Reverse	PCE (%) Forward	PCE (%) Reverse
0	24.73	24.78	0.967	1.005	0.446	0.626	10.7	15.6
72	3.02	2.96	0.954	0.953	0.237	0.238	0.7	0.7

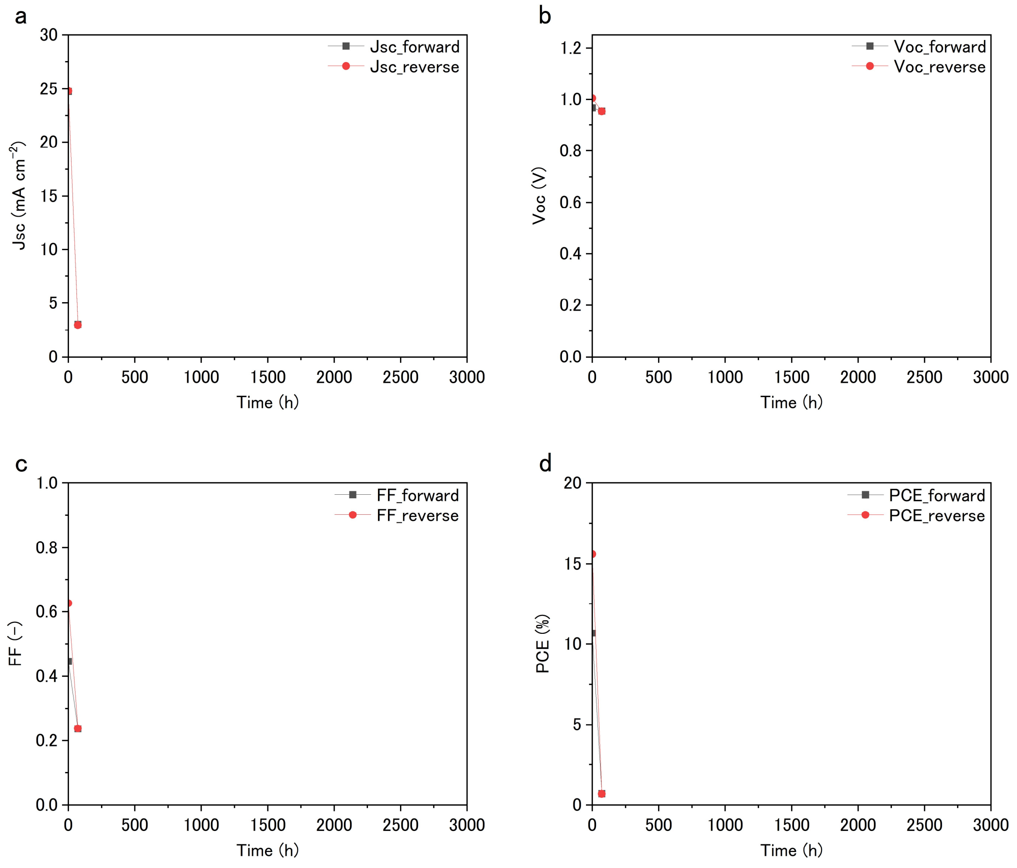

Supplementary Figure 1. Stability result of the perovskite solar cells with Li+tBP (reference). (a) J_{SC}, (b) V_{OC}, (c) *FF*, and (d) *PCE* as a function of the time with forward and reverse scan. Sample was stored at 85 °C in dark.

Supplementary Table 3. Stability result of the perovskite solar cells with Li,Co+tBP (reference). Sample was stored at 85 °C in dark.

	Jsc (mA/sq)	Jsc (mA/sq)	Voc (V)	Voc (V)	FF (-)	FF (-)	PCE (%)	PCE (%)
Time (h)	Forward	Reverse	Forward	Reverse	Forward	Reverse	Forward	Reverse
0	24.74	24.71	0.975	1.033	0.538	0.710	13.0	18.1
168	24.66	24.55	1.004	1.014	0.519	0.631	12.9	15.7
312	24.52	24.28	0.996	1.010	0.427	0.562	10.4	13.8
792	24.45	21.30	0.992	1.011	0.249	0.439	6.0	9.5
1128	24.14	16.88	0.974	0.994	0.151	0.342	3.5	5.7
1704	22.86	12.99	0.955	0.987	0.112	0.317	2.4	4.1
2856	10.10	7.87	0.927	0.929	0.186	0.253	1.7	1.8

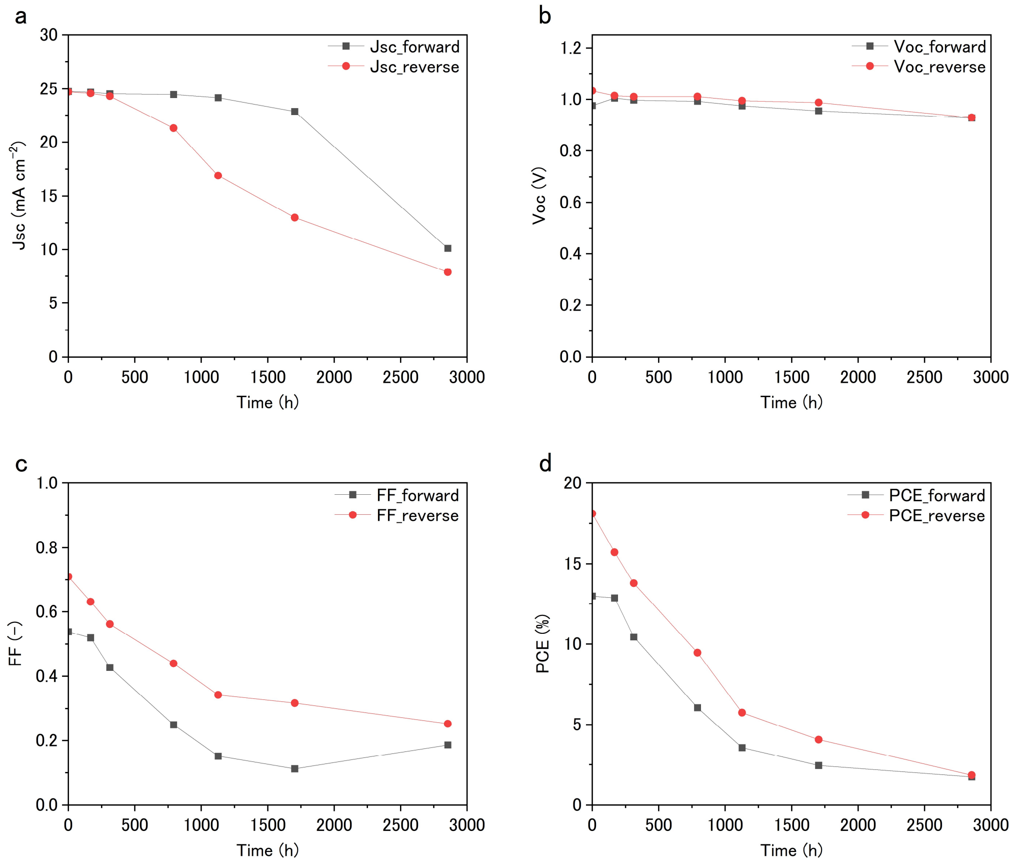

Supplementary Figure 2. Stability result of the perovskite solar cells with Li,Co+tBP (reference). (a) J_{SC}, (b) V_{OC}, (c) *FF*, and (d) *PCE* as a function of the time with forward and reverse scan. Sample was stored at 85 °C in dark.

Supplementary Table 4. Stability result of the perovskite solar cells with Li+K1. Sample was stored at 85 °C in dark.

Time (h)	Jsc (mA/sq) Forward	Jsc (mA/sq) Reverse	Voc (V) Forward	Voc (V) Reverse	FF (-) Forward	FF (-) Reverse	PCE (%) Forward	PCE (%) Reverse
0	24.88	24.88	0.805	0.877	0.507	0.632	10.1	13.8
168	25.10	25.02	0.854	0.875	0.479	0.609	10.3	13.3
312	24.99	24.94	0.853	0.883	0.480	0.617	10.2	13.6
792	24.83	24.81	0.825	0.884	0.474	0.600	9.7	13.2
1128	24.88	24.85	0.825	0.882	0.453	0.579	9.3	12.7
1704	24.81	24.74	0.798	0.877	0.448	0.562	8.9	12.2
2856	24.05	24.00	0.801	0.873	0.403	0.479	7.8	10.0

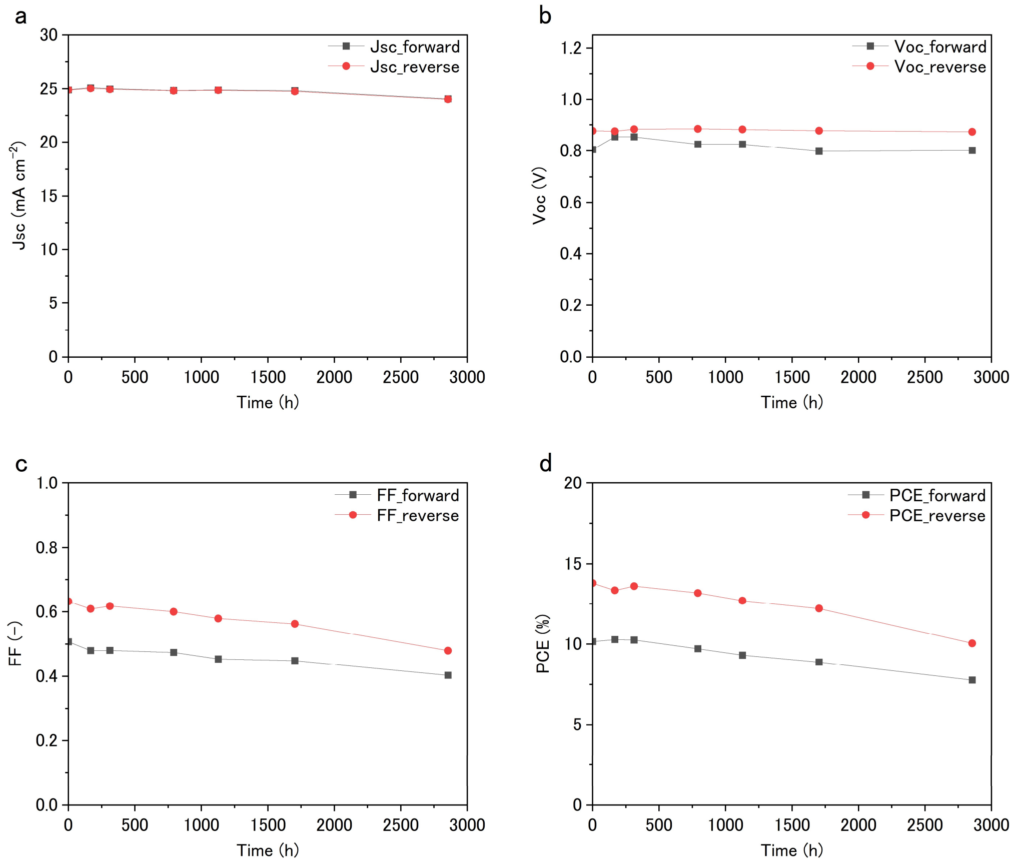

Supplementary Figure 3. Stability result of the perovskite solar cells with Li+K1. (a) J_{SC}, (b) V_{OC}, (c) *FF*, and (d) *PCE* as a function of the time with forward and reverse scan. Sample was stored at 85 °C in dark.

Supplementary Table 5. Stability result of the perovskite solar cells with Li,Co+K1. Sample was stored at 85 °C in dark.

Time (h)	Jsc (mA/sq) Forward	Jsc (mA/sq) Reverse	Voc (V) Forward	Voc (V) Reverse	FF (-) Forward	FF (-) Reverse	PCE (%) Forward	PCE (%) Reverse
0	24.79	24.81	0.803	0.871	0.430	0.534	8.6	11.5
168	24.84	24.77	0.804	0.837	0.401	0.569	8.0	11.8
288	24.91	24.86	0.801	0.840	0.405	0.554	8.1	11.6
768	24.90	24.80	0.777	0.847	0.374	0.469	7.2	9.9
1104	24.75	24.71	0.775	0.858	0.408	0.495	7.8	10.5
1776	24.71	24.67	0.752	0.842	0.397	0.494	7.4	10.3
2616	23.39	23.36	0.733	0.815	0.367	0.445	6.3	8.5

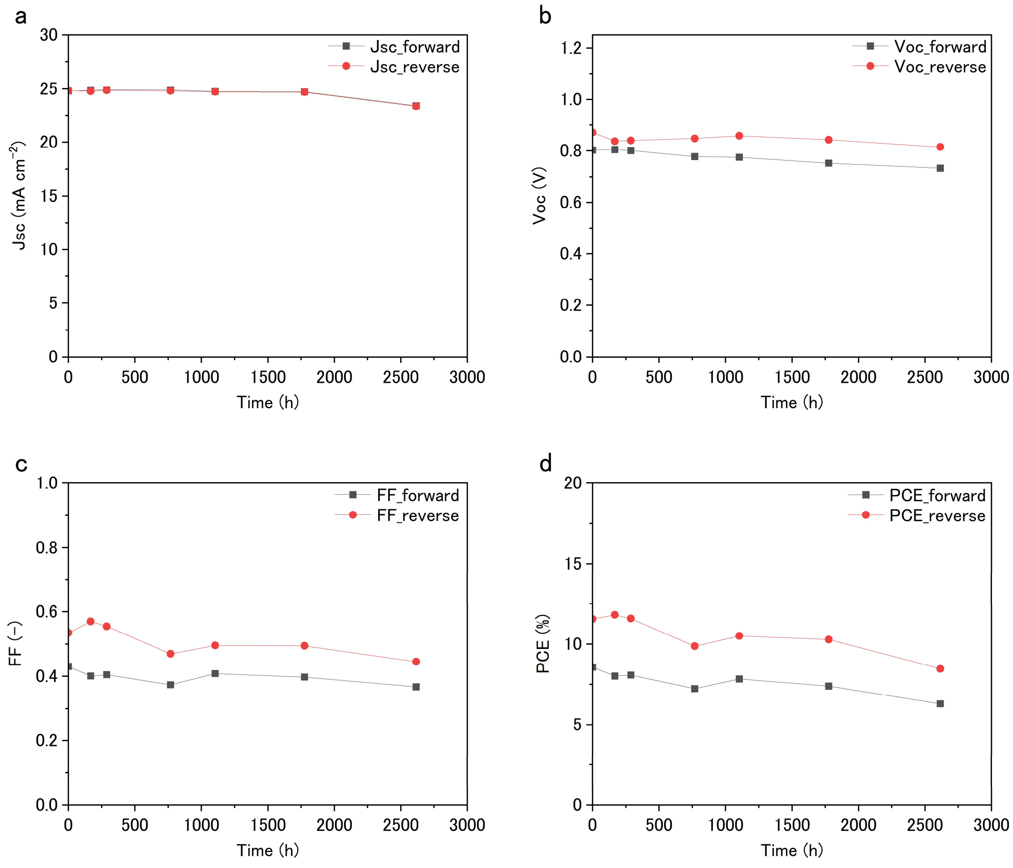

Supplementary Figure 4. Stability result of the perovskite solar cells with Li,Co+K1. (a) J_{SC}, (b) V_{OC}, (c) *FF*, and (d) *PCE* as a function of the time with forward and reverse scan. Sample was stored at 85 °C in dark.

Supplementary Table 6. Stability result of the perovskite solar cells with Li+K2. Sample was stored at 85 °C in dark.

Time (h)	Jsc (mA/sq) Forward	Jsc (mA/sq) Reverse	Voc (V) Forward	Voc (V) Reverse	FF (-) Forward	FF (-) Reverse	PCE (%) Forward	PCE (%) Reverse
0	24.85	24.86	0.884	0.914	0.455	0.558	10.0	12.7
168	24.88	24.87	0.862	0.880	0.505	0.679	10.8	14.9
312	24.77	24.75	0.868	0.891	0.499	0.669	10.7	14.8
1128	24.66	24.62	0.835	0.890	0.492	0.620	10.1	13.6
1704	24.59	24.57	0.815	0.885	0.480	0.601	9.6	13.1
2856	24.03	24.03	0.777	0.863	0.472	0.580	8.8	12.0

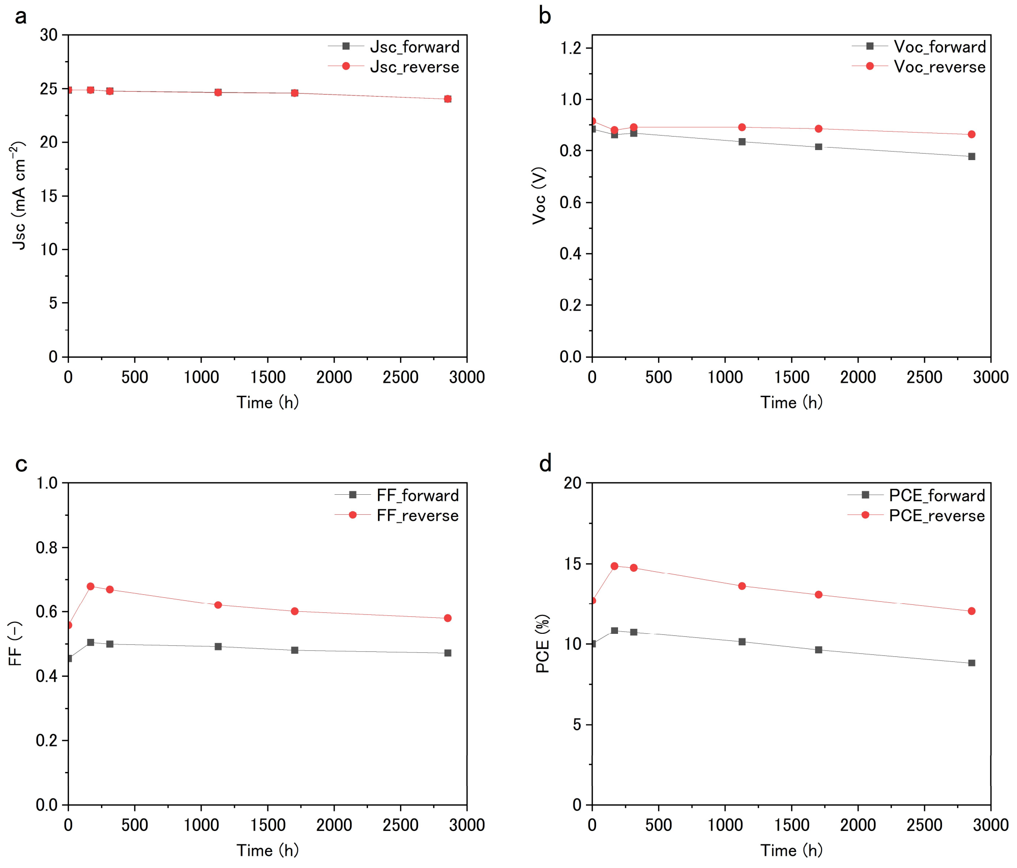

Supplementary Figure 5. Stability result of the perovskite solar cells with Li+K2. (a) J_{SC}, (b) V_{OC}, (c) *FF*, and (d) *PCE* as a function of the time with forward and reverse scan. Sample was stored at 85 °C in dark.

Supplementary Table 7. Stability result of the perovskite solar cells with Li,Co+K2. Sample was stored at 85 °C in dark.

Time (h)	Jsc (mA/sq) Forward	Jsc (mA/sq) Reverse	Voc (V) Forward	Voc (V) Reverse	FF (-) Forward	FF (-) Reverse	PCE (%) Forward	PCE (%) Reverse
0	24.82	24.74	0.823	0.897	0.467	0.564	9.5	12.5
168	24.82	24.83	0.832	0.859	0.456	0.626	9.4	13.4
288	24.81	24.76	0.837	0.869	0.437	0.580	9.1	12.5
768	24.74	24.54	0.813	0.861	0.321	0.385	6.5	8.1
1104	24.65	24.57	0.783	0.857	0.392	0.473	7.6	10.0
1776	24.51	24.45	0.775	0.841	0.351	0.418	6.7	8.6
2616	23.31	22.42	0.754	0.802	0.274	0.323	4.8	5.8

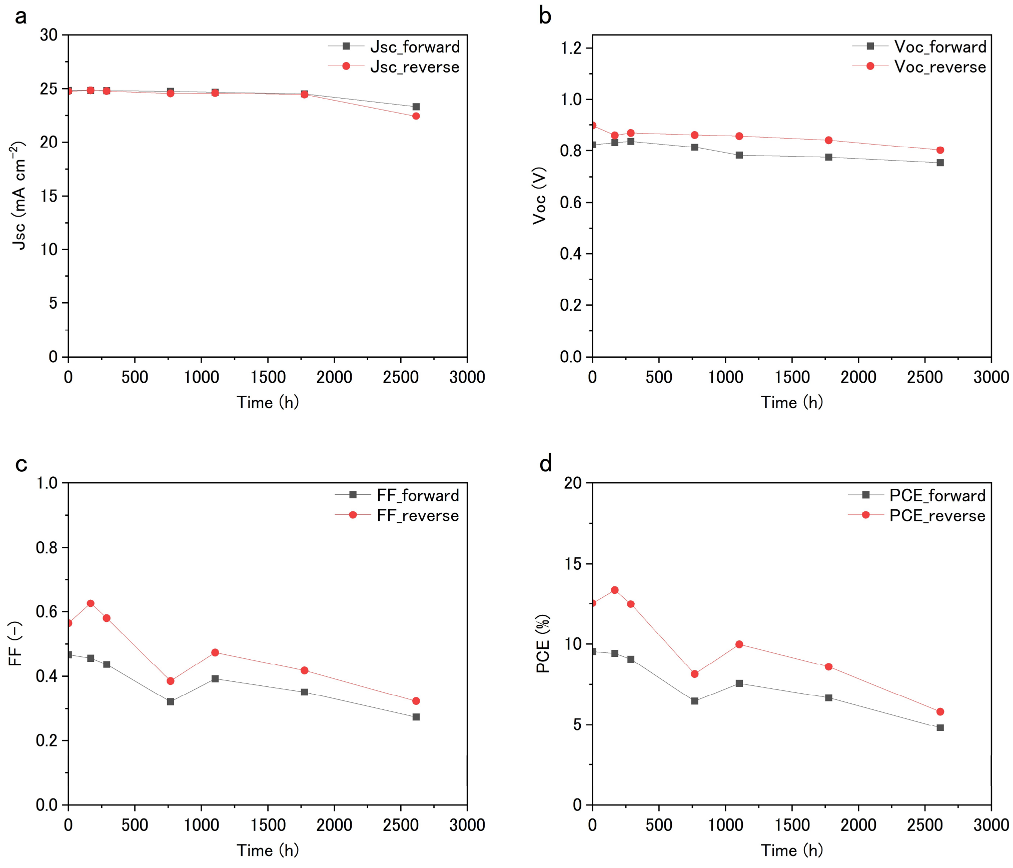

Supplementary Figure 6. Stability result of the perovskite solar cells with Li,Co+K2. (a) J_{SC}, (b) V_{OC}, (c) *FF*, and (d) *PCE* as a function of the time with forward and reverse scan. Sample was stored at 85 °C in dark.

Supplementary Table 8. Stability result of the perovskite solar cells with Li+K3. Sample was stored at 85 °C in dark.

Time (h)	Jsc (mA/sq) Forward	Jsc (mA/sq) Reverse	Voc (V) Forward	Voc (V) Reverse	FF (-) Forward	FF (-) Reverse	PCE (%) Forward	PCE (%) Reverse
0	24.73	24.69	0.817	0.883	0.511	0.626	10.3	13.6
168	24.65	24.67	0.838	0.868	0.459	0.647	9.5	13.8
312	24.55	24.59	0.853	0.899	0.470	0.629	9.8	13.9
792	23.91	23.92	0.793	0.882	0.446	0.556	8.5	11.7
1704	24.28	24.23	0.768	0.857	0.411	0.529	7.7	11.0
2856	23.70	23.66	0.727	0.817	0.378	0.485	6.5	9.4

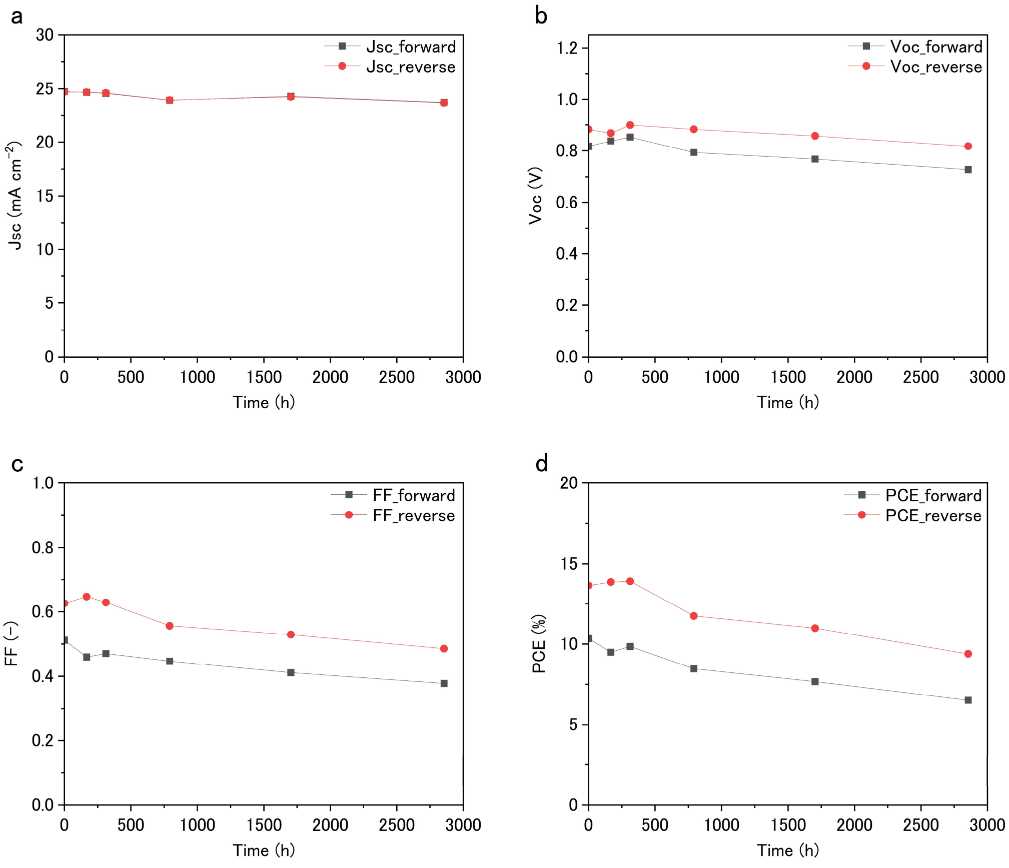

Supplementary Figure 7. Stability result of the perovskite solar cells with Li+K3. (a) J_{SC}, (b) V_{OC}, (c) *FF*, and (d) *PCE* as a function of the time with forward and reverse scan. Sample was stored at 85 °C in dark.

Supplementary Table 9. Stability result of the perovskite solar cells with Li,Co+K3. Sample was stored at 85 °C in dark.

Time (h)	Jsc (mA/sq) Forward	Jsc (mA/sq) Reverse	Voc (V) Forward	Voc (V) Reverse	FF (-) Forward	FF (-) Reverse	PCE (%) Forward	PCE (%) Reverse
0	24.60	22.53	0.765	0.828	0.248	0.303	4.7	5.7
168	24.41	21.14	0.808	0.894	0.291	0.431	5.7	8.1
288	24.56	24.08	0.844	0.913	0.402	0.519	8.3	11.4
768	24.39	23.60	0.816	0.906	0.313	0.437	6.2	9.3
1104	24.24	22.69	0.782	0.886	0.262	0.393	5.0	7.9
1776	23.79	18.93	0.708	0.826	0.168	0.288	2.8	4.5
2616	12.98	9.86	0.695	0.767	0.139	0.226	1.3	1.7

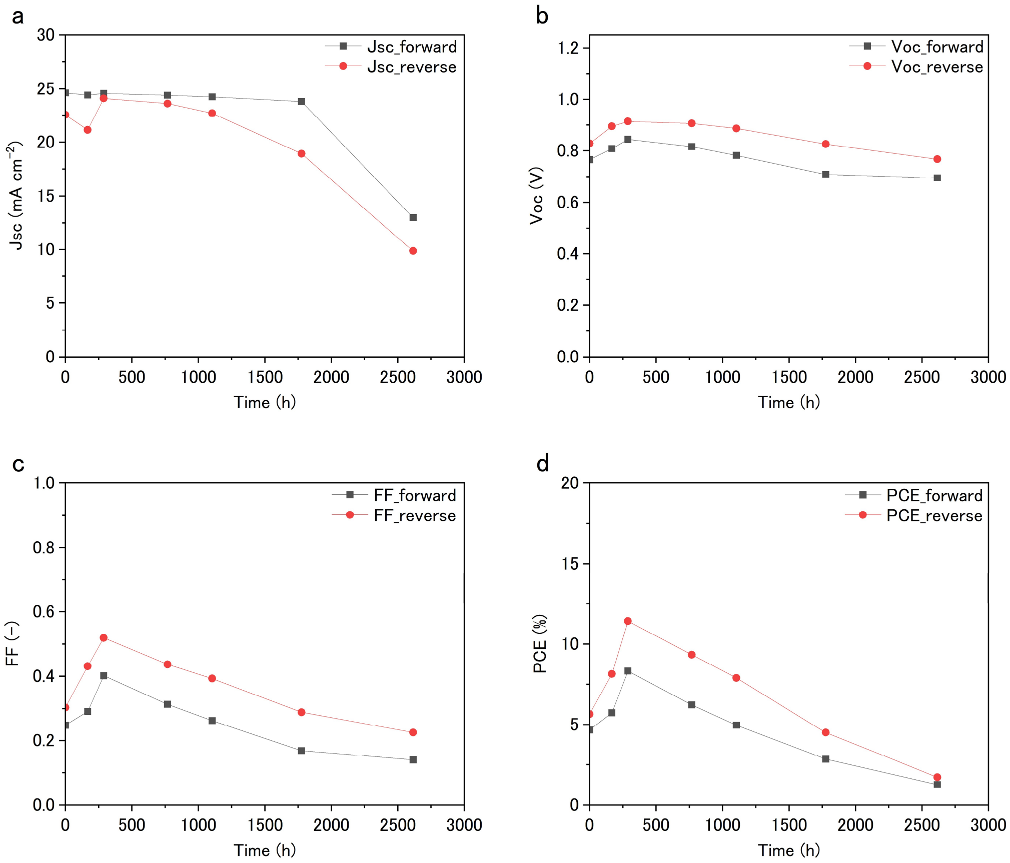

Supplementary Figure 8. Stability result of the perovskite solar cells with Li,Co+K3. (a) J_{SC}, (b) V_{OC}, (c) *FF*, and (d) *PCE* as a function of the time with forward and reverse scan. Sample was stored at 85 °C in dark.

Supplementary Table 10. Stability result of the perovskite solar cells with Li+K4. Sample was stored at 85 ℃ in dark.

Time (h)	Jsc (mA/sq) Forward	Jsc (mA/sq) Reverse	Voc (V) Forward	Voc (V) Reverse	FF (-) Forward	FF (-) Reverse	PCE (%) Forward	PCE (%) Reverse
0	24.82	24.85	0.751	0.811	0.454	0.582	8.5	11.7
168	24.87	24.92	0.816	0.838	0.409	0.565	8.3	11.8
312	24.80	24.81	0.815	0.844	0.403	0.574	8.1	12.0
792	24.68	24.63	0.781	0.850	0.425	0.553	8.2	11.6
1128	24.59	24.58	0.773	0.848	0.405	0.519	7.7	10.8
2856	24.16	24.11	0.727	0.826	0.397	0.499	7.0	9.9

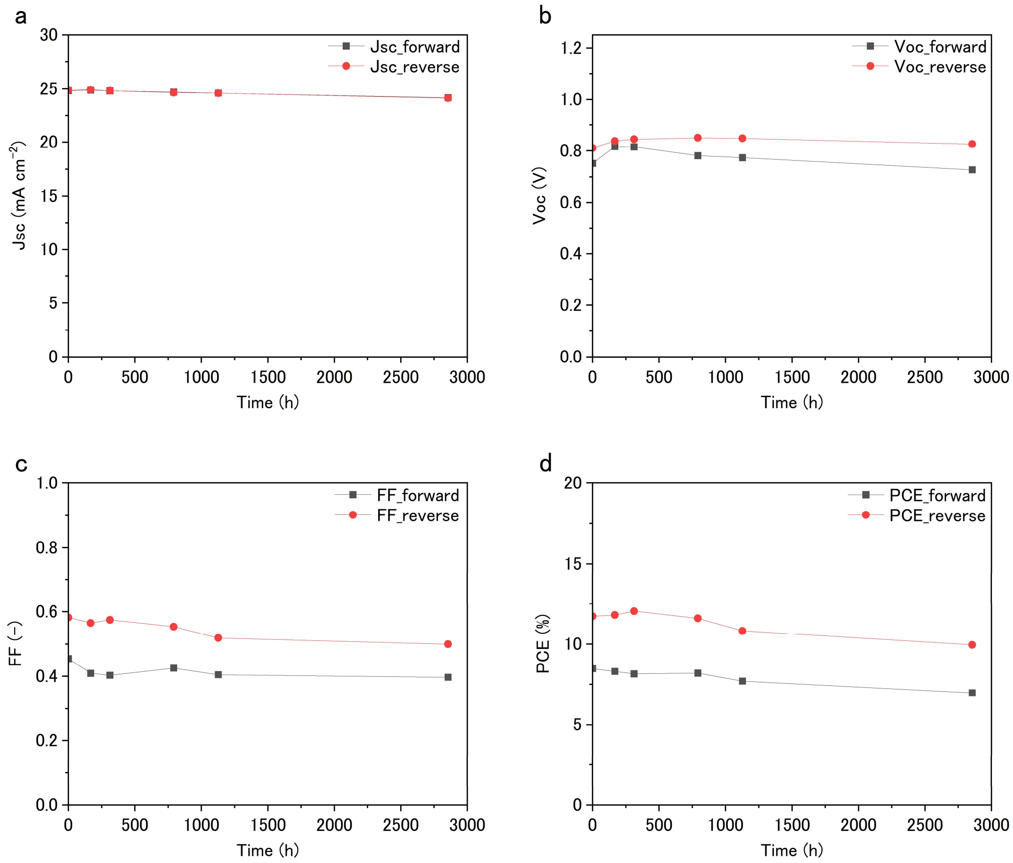

Supplementary Figure 9. Stability result of the perovskite solar cells with Li+K4. (a) J_{SC}, (b) V_{OC}, (c) *FF*, and (d) *PCE* as a function of the time with forward and reverse scan. Sample was stored at 85 °C in dark.

Supplementary Table 11. Stability result of the perovskite solar cells with Li+K5. Sample was stored at 85 °C in dark.

Time (h)	Jsc (mA/sq) Forward	Jsc (mA/sq) Reverse	Voc (V) Forward	Voc (V) Reverse	FF (-) Forward	FF (-) Reverse	PCE (%) Forward	PCE (%) Reverse
0	24.57	24.15	0.732	0.767	0.338	0.486	6.1	9.0
72	18.80	19.30	0.804	0.827	0.474	0.504	7.2	8.1
168	17.17	17.23	0.743	0.795	0.371	0.427	4.7	5.9
336	14.91	14.79	0.671	0.753	0.300	0.358	3.0	4.0
504	13.87	13.83	0.640	0.741	0.277	0.323	2.5	3.3

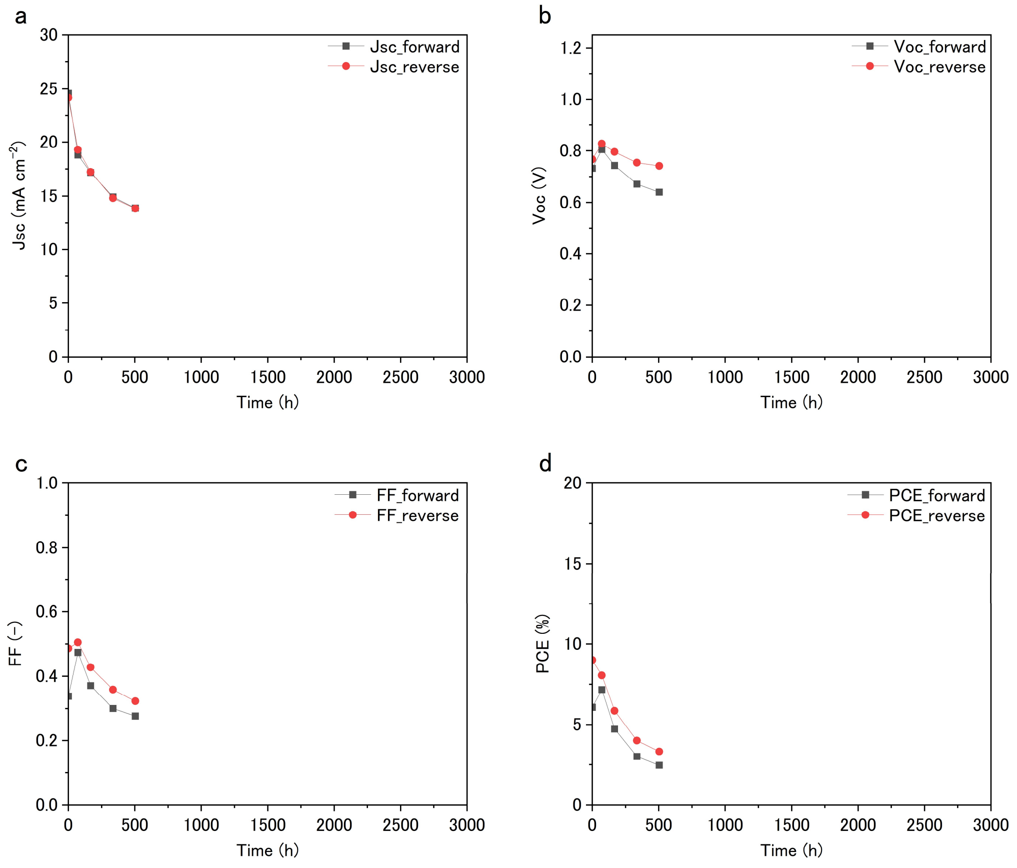

Supplementary Figure 10. Stability result of the perovskite solar cells with Li+K5. (a) J_{SC}, (b) V_{OC}, (c) FF, and (d) PCE as a function of the time with forward and reverse scan. Sample was stored at 85 °C in dark.

Supplementary Table 12. Stability result of the perovskite solar cells with Li,Co+K5. Sample was stored at 85 °C in dark.

Time (h)	Jsc (mA/sq) Forward	Jsc (mA/sq) Reverse	Voc (V) Forward	Voc (V) Reverse	FF (-) Forward	FF (-) Reverse	PCE (%) Forward	PCE (%) Reverse
0	25.53	25.48	0.817	0.850	0.440	0.573	9.2	12.4
72	21.65	18.91	0.714	0.758	0.282	0.391	4.4	5.6

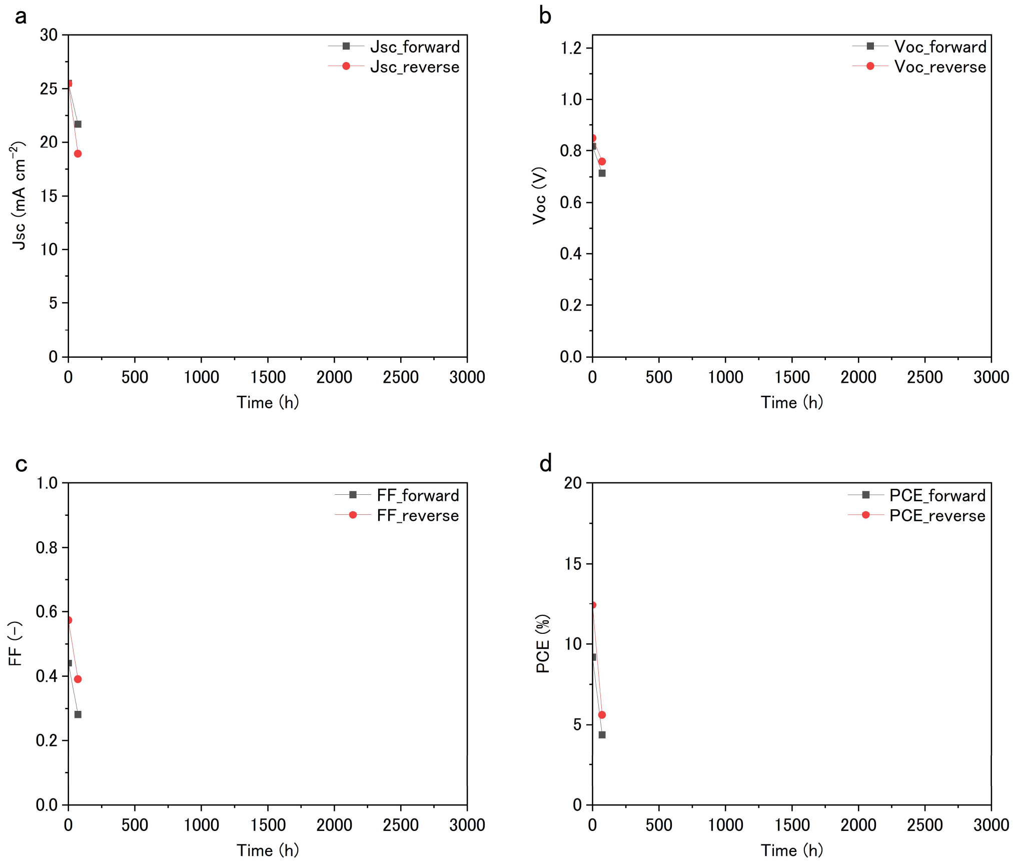

Supplementary Figure 11. Stability result of the perovskite solar cells with Li,Co+K5. (a) J_{SC}, (b) V_{OC}, (c) FF, and (d) PCE as a function of the time with forward and reverse scan. Sample was stored at 85 °C in dark.

Supplementary Table 13. Stability result of the perovskite solar cells with Li+K6. Sample was stored at 85 °C in dark.

	Jsc (mA/sq)	Jsc (mA/sq)	Voc (V)	Voc (V)	FF (-)	FF (-)	PCE (%)	PCE (%)
Time (h)	Forward	Reverse	Forward	Reverse	Forward	Reverse	Forward	Reverse
0	24.42	24.39	0.727	0.810	0.423	0.581	7.5	11.5
72	12.36	12.37	0.846	0.844	0.252	0.258	2.6	2.7

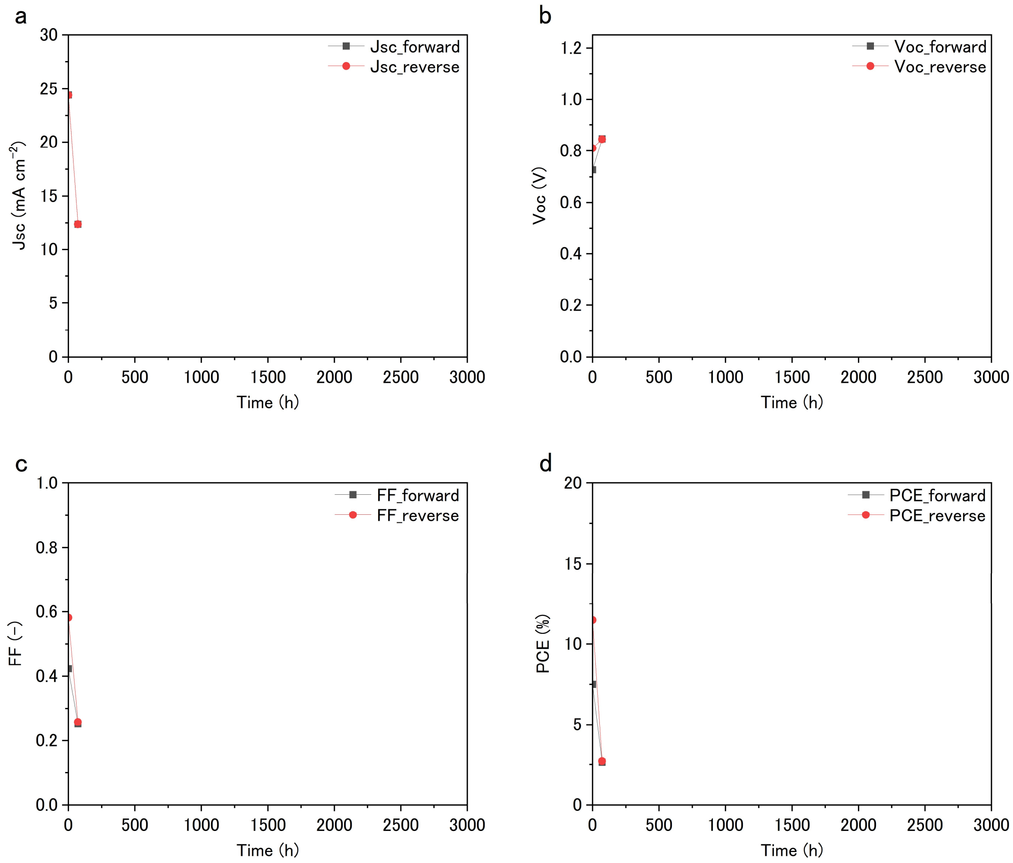

Supplementary Figure 12. Stability result of the perovskite solar cells with Li+K6. (a) J_{SC}, (b) V_{OC}, (c) *FF*, and (d) *PCE* as a function of the time with forward and reverse scan. Sample was stored at 85 °C in dark.

Supplementary Table 14. Stability result of the perovskite solar cells with Li,Co+K6. Sample was stored at 85 °C in dark.

Time (h)	Jsc (mA/sq) Forward	Jsc (mA/sq) Reverse	Voc (V) Forward	Voc (V) Reverse	FF (-) Forward	FF (-) Reverse	PCE (%) Forward	PCE (%) Reverse
0	25.48	25.45	0.896	0.955	0.450	0.600	10.3	14.6
72	24.65	24.63	0.902	0.908	0.495	0.647	11.0	14.5
168	24.74	24.74	0.890	0.907	0.535	0.690	11.8	15.5
336	24.73	24.78	0.888	0.904	0.509	0.633	11.2	14.2
504	24.75	24.73	0.884	0.899	0.462	0.564	10.1	12.5
624	24.66	24.69	0.880	0.898	0.466	0.571	10.1	12.7
1104	24.56	24.53	0.866	0.890	0.398	0.478	8.5	10.4
1440	24.22	24.18	0.834	0.888	0.469	0.575	9.5	12.4
1944	24.17	23.57	0.844	0.878	0.305	0.359	6.2	7.4
2784	17.05	15.75	0.833	0.853	0.231	0.265	3.3	3.6

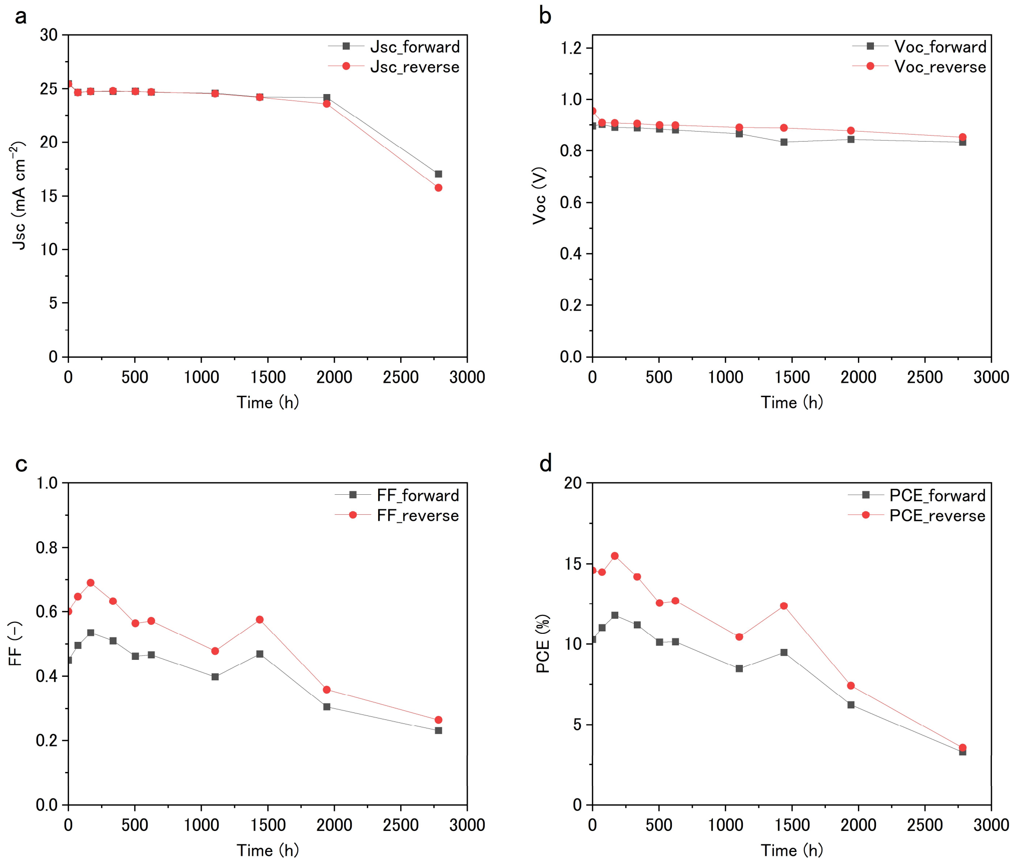

Supplementary Figure 13. Stability result of the perovskite solar cells with Li,Co+K6. (a) J_{SC}, (b) V_{OC}, (c) *FF*, and (d) *PCE* as a function of the time with forward and reverse scan. Sample was stored at 85 °C in dark.

Supplementary Table 15. Stability result of the perovskite solar cells with Li+K7. Sample was stored at 85 °C in dark.

Time (h)	Jsc (mA/sq) Forward	Jsc (mA/sq) Reverse	Voc (V) Forward	Voc (V) Reverse	FF (-) Forward	FF (-) Reverse	PCE (%) Forward	PCE (%) Reverse
0	24.77	24.70	0.774	0.810	0.329	0.424	6.3	8.5
168	24.69	24.56	0.822	0.837	0.385	0.517	7.8	10.6
792	24.37	24.30	0.799	0.845	0.374	0.458	7.3	9.4
1128	24.34	24.08	0.805	0.848	0.322	0.403	6.3	8.2
1704	24.36	24.05	0.783	0.842	0.336	0.422	6.4	8.6
2856	23.70	23.24	0.744	0.824	0.331	0.426	5.8	8.1

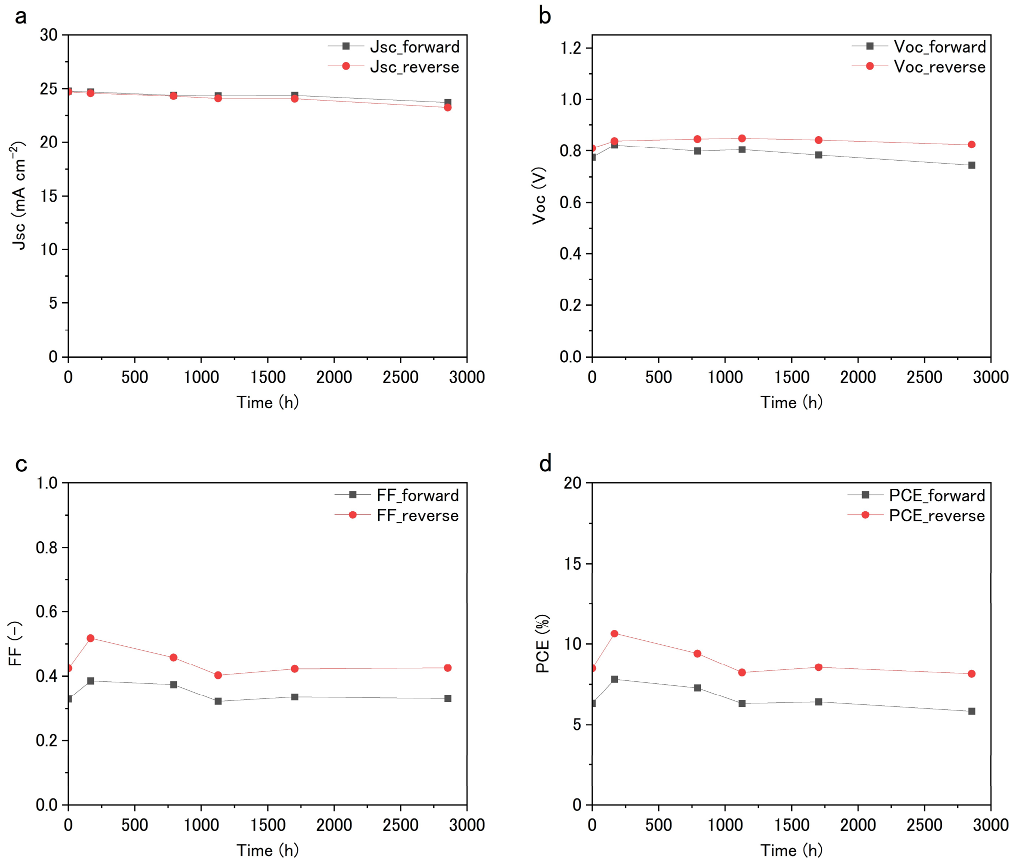

Supplementary Figure 14. Stability result of the perovskite solar cells with Li+K7. (a) J_{SC}, (b) V_{OC}, (c) *FF*, and (d) *PCE* as a function of the time with forward and reverse scan. Sample was stored at 85 °C in dark.

Supplementary Table 16. Stability result of the perovskite solar cells with Li,Co+K7. Sample was stored at 85 °C in dark.

Time (h)	Jsc (mA/sq) Forward	Jsc (mA/sq) Reverse	Voc (V) Forward	Voc (V) Reverse	FF (-) Forward	FF (-) Reverse	PCE (%) Forward	PCE (%) Reverse
0	24.48	24.51	0.786	0.825	0.431	0.506	8.3	10.2
168	24.36	23.19	0.845	0.869	0.334	0.480	6.9	9.7
288	24.34	23.34	0.839	0.867	0.342	0.476	7.0	9.6
768	24.17	23.16	0.804	0.862	0.309	0.406	6.0	8.1
1104	23.89	22.93	0.773	0.856	0.293	0.386	5.4	7.6
1776	23.22	22.33	0.745	0.841	0.274	0.349	4.7	6.5
2616	21.43	20.42	0.719	0.804	0.254	0.327	3.9	5.4

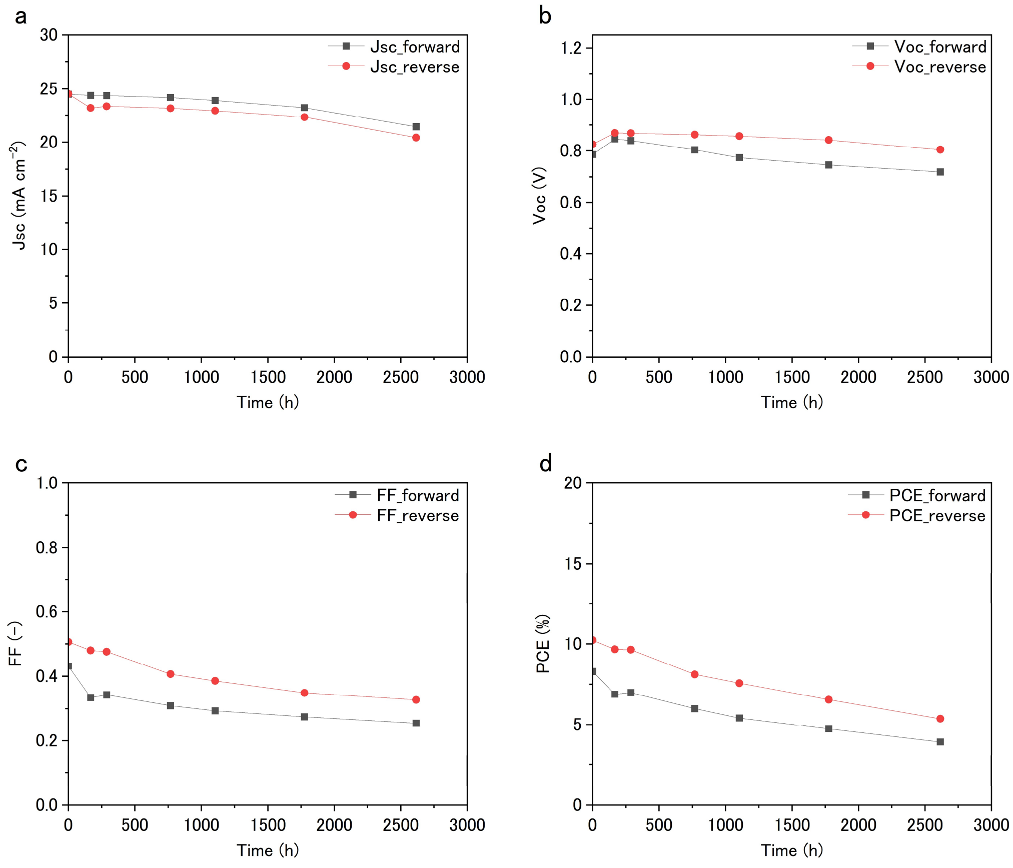

Supplementary Figure 15. Stability result of the perovskite solar cells with Li,Co+K7. (a) J_{SC}, (b) V_{OC}, (c) *FF*, and (d) *PCE* as a function of the time with forward and reverse scan. Sample was stored at 85 °C in dark.

Supplementary Table 17. Stability result of the perovskite solar cells with Li+K8. Sample was stored at 85 °C in dark.

Time (h)	Jsc (mA/sq) Forward	Jsc (mA/sq) Reverse	Voc (V) Forward	Voc (V) Reverse	FF (-) Forward	FF (-) Reverse	PCE (%) Forward	PCE (%) Reverse
0	24.94	24.88	0.763	0.819	0.397	0.496	7.6	10.1
168	24.65	24.62	0.869	0.879	0.513	0.649	11.0	14.0
312	25.07	25.11	0.851	0.875	0.490	0.639	10.5	14.0
792	24.79	24.80	0.814	0.873	0.505	0.632	10.2	13.7
1128	24.76	24.78	0.807	0.873	0.492	0.609	9.8	13.2
1704	24.66	24.66	0.796	0.874	0.490	0.599	9.6	12.9
2856	24.18	24.20	0.774	0.865	0.482	0.573	9.0	12.0

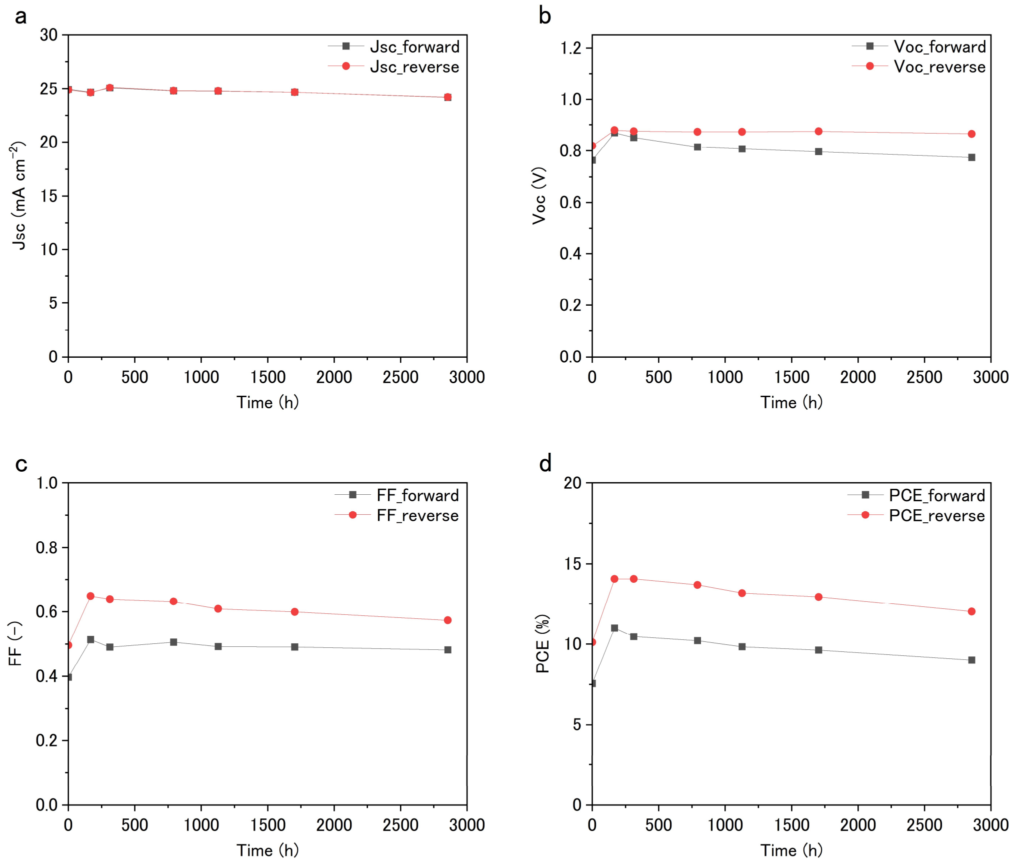

Supplementary Figure 16. Stability result of the perovskite solar cells with Li+K8. (a) J_{SC}, (b) V_{OC}, (c) *FF*, and (d) *PCE* as a function of the time with forward and reverse scan. Sample was stored at 85 °C in dark.

Supplementary Table 18. Stability result of the perovskite solar cells with Li,Co+K8. Sample was stored at 85 °C in dark.

Time (h)	Jsc (mA/sq) Forward	Jsc (mA/sq) Reverse	Voc (V) Forward	Voc (V) Reverse	FF (-) Forward	FF (-) Reverse	PCE (%) Forward	PCE (%) Reverse
0	24.81	24.78	0.848	0.880	0.535	0.636	11.3	13.9
168	24.76	24.80	0.867	0.883	0.512	0.670	11.0	14.7
288	23.25	23.24	0.845	0.878	0.500	0.659	9.8	13.4
768	24.53	24.49	0.835	0.888	0.463	0.582	9.5	12.7
1104	24.41	24.42	0.832	0.885	0.406	0.541	8.2	11.7
1776	24.36	24.22	0.775	0.852	0.422	0.540	8.0	11.1
2616	23.49	23.33	0.775	0.851	0.404	0.493	7.3	9.8

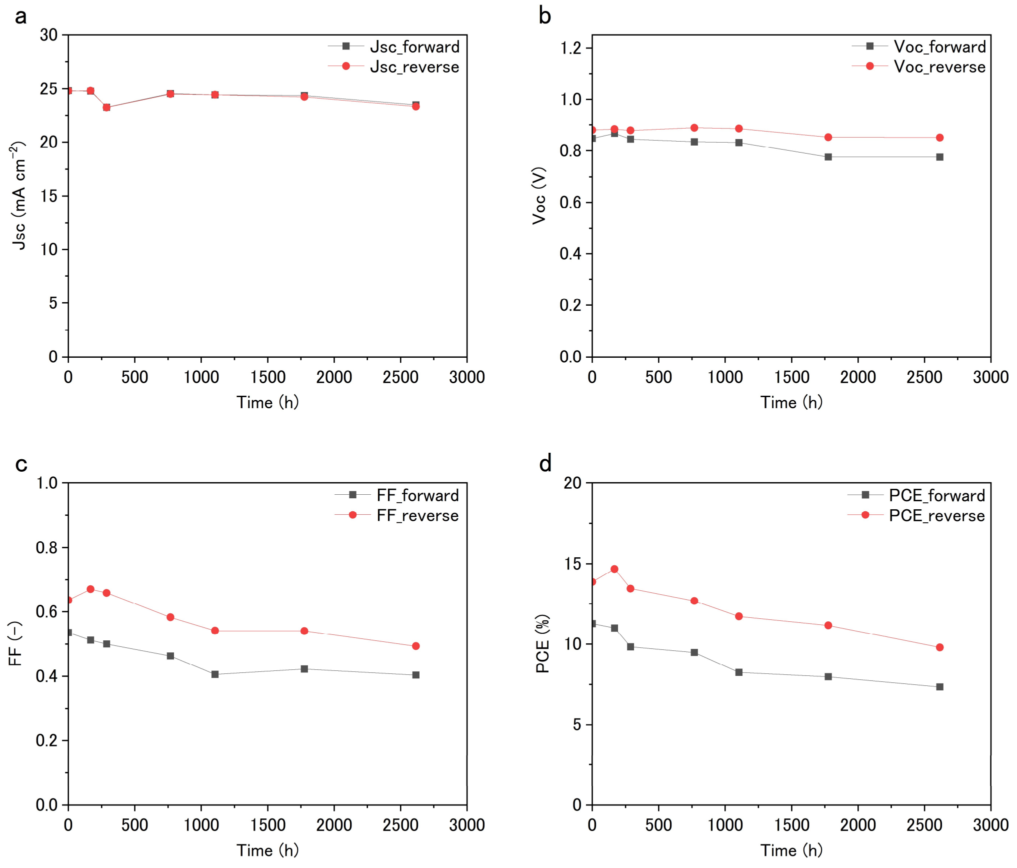

Supplementary Figure 17. Stability result of the perovskite solar cells with Li,Co+K8. (a) J_{SC}, (b) V_{OC}, (c) FF, and (d) PCE as a function of the time with forward and reverse scan. Sample was stored at 85 °C in dark.

Supplementary Table 19. Stability result of the perovskite solar cells with Li+K9. Sample was stored at 85 °C in dark.

Time (h)	Jsc (mA/sq) Forward	Jsc (mA/sq) Reverse	Voc (V) Forward	Voc (V) Reverse	FF (-) Forward	FF (-) Reverse	PCE (%) Forward	PCE (%) Reverse
0	17.31	15.18	0.649	0.708	0.148	0.224	1.7	2.4
168	16.44	16.56	0.669	0.719	0.292	0.401	3.2	4.8
312	14.72	14.67	0.534	0.636	0.278	0.388	2.2	3.6
792	12.82	12.71	0.455	0.584	0.289	0.389	1.7	2.9
1128	12.54	12.33	0.459	0.585	0.292	0.391	1.7	2.8
1704	12.46	12.15	0.454	0.555	0.234	0.336	1.3	2.3
2856	11.67	11.32	0.455	0.565	0.241	0.345	1.3	2.2

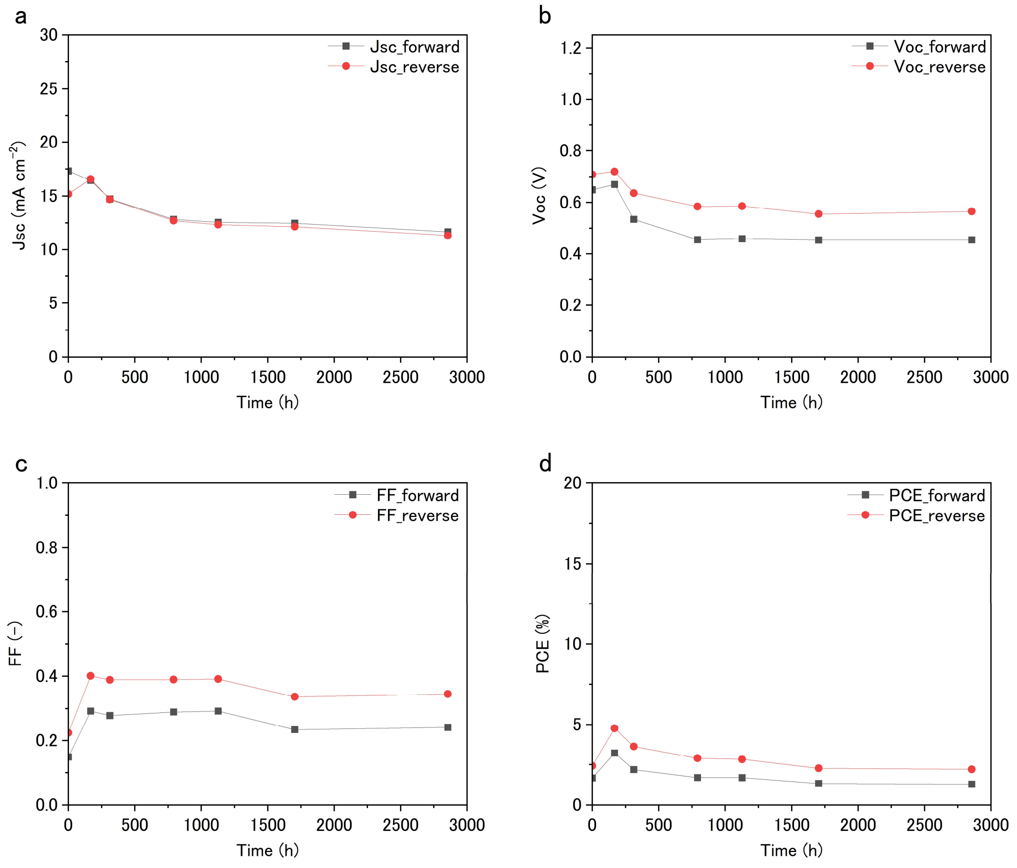

Supplementary Figure 18. Stability result of the perovskite solar cells with Li+K9. (a) J_{SC}, (b) V_{OC}, (c) FF, and (d) PCE as a function of the time with forward and reverse scan. Sample was stored at 85 °C in dark.

Supplementary Table 20. Stability result of the perovskite solar cells with Li,Co+K9. Sample was stored at 85 °C in dark.

Time (h)	Jsc (mA/sq) Forward	Jsc (mA/sq) Reverse	Voc (V) Forward	Voc (V) Reverse	FF (-) Forward	FF (-) Reverse	PCE (%) Forward	PCE (%) Reverse
0	25.03	24.32	0.996	1.049	0.369	0.475	9.2	12.1
168	24.94	24.67	0.963	0.975	0.362	0.437	8.7	10.5
288	24.49	23.02	0.942	0.946	0.277	0.337	6.4	7.3
768	17.00	14.15	0.925	0.947	0.201	0.265	3.2	3.5
1104	20.76	16.98	0.887	0.931	0.206	0.290	3.8	4.6
1776	15.49	13.09	0.849	0.889	0.208	0.276	2.7	3.2
2616	5.82	5.36	0.814	0.833	0.217	0.242	1.0	1.1

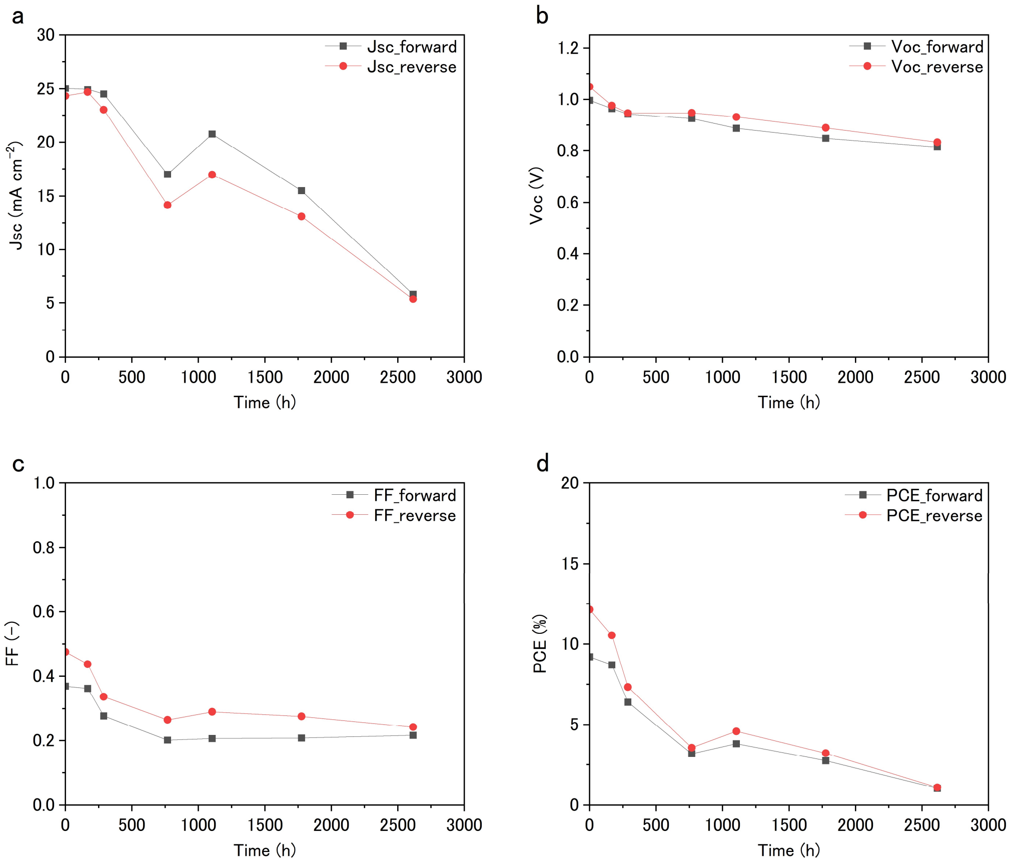

Supplementary Figure 19. Stability result of the perovskite solar cells with Li,Co+K9. (a) J_{SC}, (b) V_{OC}, (c) *FF*, and (d) *PCE* as a function of the time with forward and reverse scan. Sample was stored at 85 °C in dark.

Supplementary Table 21. Stability result of the perovskite solar cells with Li+K10. Sample was stored at 85 °C in dark.

Time (h)	Jsc (mA/sq) Forward	Jsc (mA/sq) Reverse	Voc (V) Forward	Voc (V) Reverse	FF (-) Forward	FF (-) Reverse	PCE (%) Forward	PCE (%) Reverse
0	24.65	24.61	0.747	0.805	0.325	0.470	6.0	9.3
72	17.94	17.33	0.833	0.831	0.236	0.253	3.5	3.6

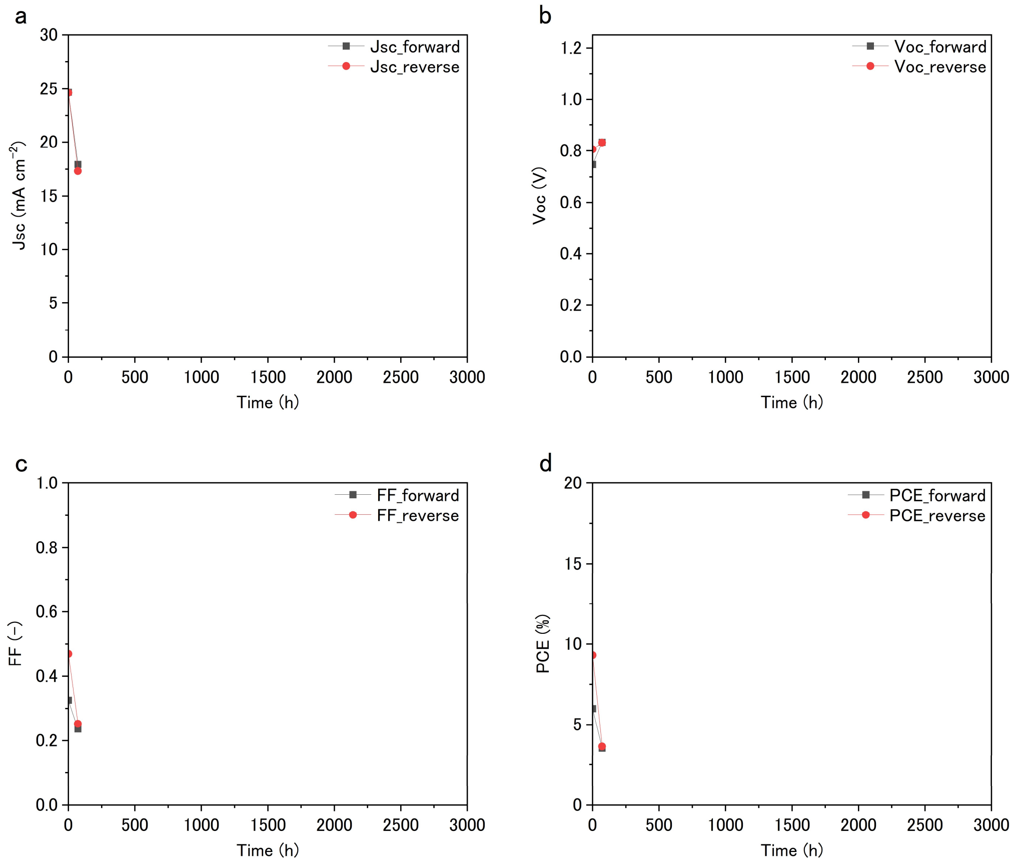

Supplementary Figure 20. Stability result of the perovskite solar cells with Li+K10. (a) J_{SC}, (b) V_{OC}, (c) *FF*, and (d) *PCE* as a function of the time with forward and reverse scan. Sample was stored at 85 °C in dark.

Supplementary Table 22. Stability result of the perovskite solar cells with Li,Co+K10. Sample was stored at 85 °C in dark.

Time (h)	Jsc (mA/sq) Forward	Jsc (mA/sq) Reverse	Voc (V) Forward	Voc (V) Reverse	FF (-) Forward	FF (-) Reverse	PCE (%) Forward	PCE (%) Reverse
0	25.26	25.23	0.934	0.948	0.453	0.670	10.7	16.0
72	24.35	24.32	0.888	0.894	0.607	0.678	13.1	14.7
168	24.47	24.45	0.892	0.905	0.632	0.713	13.8	15.8
336	24.46	24.43	0.915	0.923	0.613	0.691	13.7	15.6
504	24.31	24.26	0.925	0.931	0.605	0.676	13.6	15.3
624	24.36	24.36	0.924	0.931	0.584	0.664	13.1	15.1
1104	24.20	24.12	0.922	0.930	0.555	0.632	12.4	14.2
1440	24.17	24.13	0.922	0.930	0.541	0.627	12.0	14.1
1944	24.04	24.01	0.925	0.931	0.478	0.533	10.6	11.9
2784	22.90	22.39	0.923	0.924	0.348	0.387	7.4	8.0

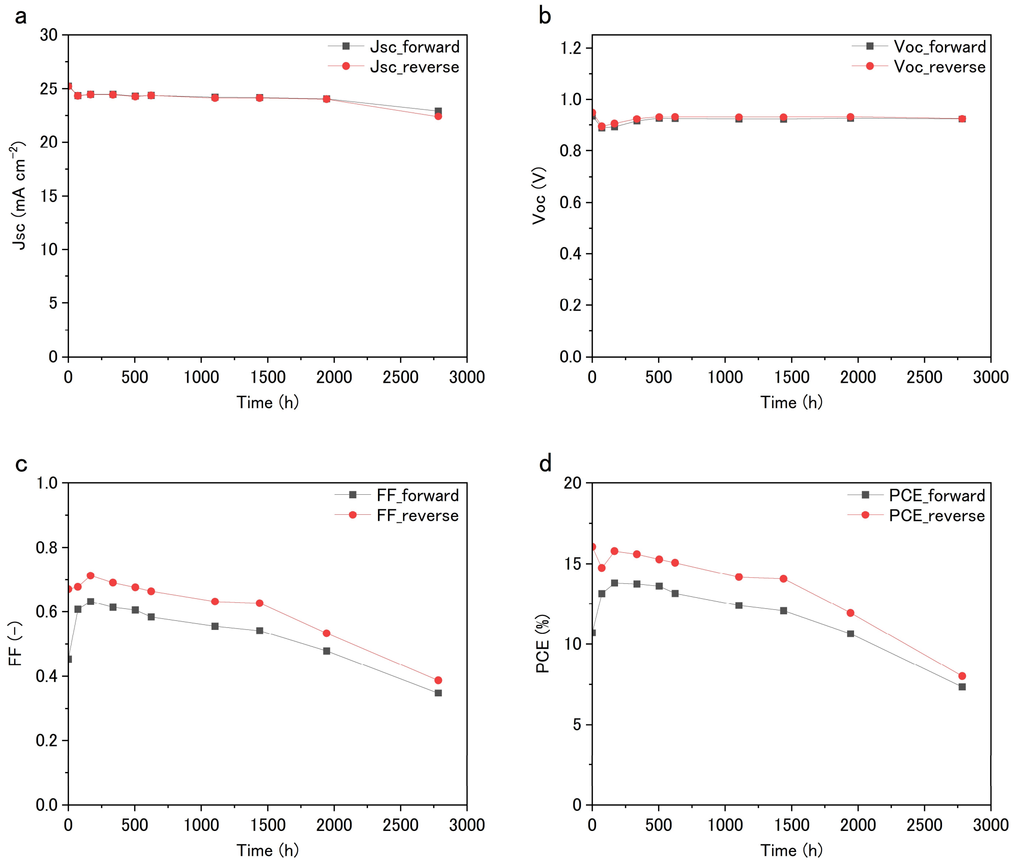

Supplementary Figure 21. Stability result of the perovskite solar cells with Li,Co+K10. (a) J_{SC}, (b) V_{OC}, (c) *FF*, and (d) *PCE* as a function of the time with forward and reverse scan. Sample was stored at 85 °C in dark.

Supplementary Table 23. Stability result of the perovskite solar cells with Li+K11. Sample was stored at 85 °C in dark.

Time (h)	Jsc (mA/sq) Forward	Jsc (mA/sq) Reverse	Voc (V) Forward	Voc (V) Reverse	FF (-) Forward	FF (-) Reverse	PCE (%) Forward	PCE (%) Reverse
0	24.04	24.01	0.733	0.802	0.519	0.592	9.2	11.4
168	22.41	20.91	0.867	0.884	0.413	0.507	8.0	9.4
312	22.09	21.51	0.826	0.872	0.404	0.518	7.4	9.7
792	21.75	21.22	0.775	0.845	0.389	0.490	6.6	8.8
1128	20.40	19.64	0.743	0.819	0.335	0.485	5.1	7.8
1704	21.39	20.70	0.705	0.806	0.368	0.464	5.6	7.7
2856	18.37	16.95	0.768	0.795	0.204	0.260	2.9	3.5

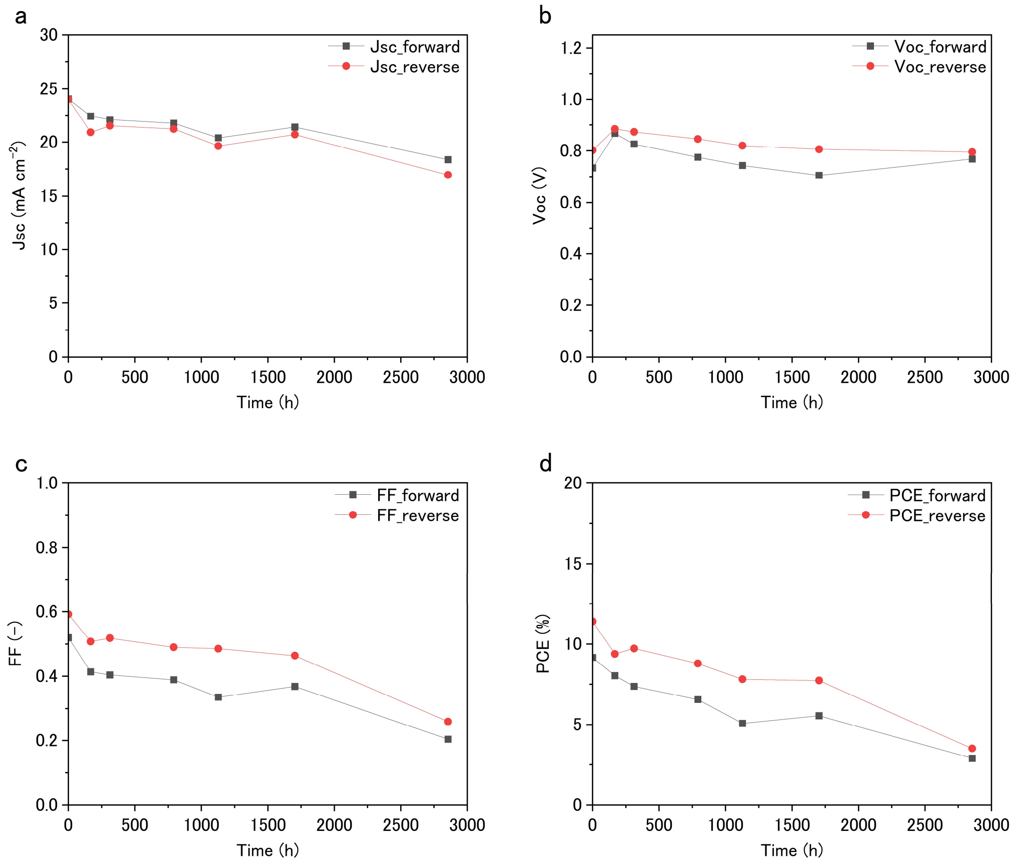

Supplementary Figure 22. Stability result of the perovskite solar cells with Li+K11. (a) J_{SC}, (b) V_{OC}, (c) *FF*, and (d) *PCE* as a function of the time with forward and reverse scan. Sample was stored at 85 °C in dark.

Supplementary Table 24. Stability result of the perovskite solar cells with Li,Co+K11. Sample was stored at 85 °C in dark.

	Jsc (mA/sq)	Jsc (mA/sq)	Voc (V)	Voc (V)	FF (-)	FF (-)	PCE (%)	PCE (%)
Time (h)	Forward	Reverse	Forward	Reverse	Forward	Reverse	Forward	Reverse
0	24.43	24.38	0.724	0.775	0.432	0.489	7.6	9.2
168	23.18	22.08	0.798	0.847	0.438	0.588	8.1	11.0
288	22.24	21.10	0.786	0.850	0.408	0.554	7.1	9.9
768	21.29	20.61	0.768	0.847	0.359	0.444	5.9	7.8
1104	19.23	17.97	0.754	0.823	0.296	0.372	4.3	5.5
1776	17.52	16.35	0.702	0.768	0.243	0.336	3.0	4.2
2616	16.74	15.56	0.707	0.782	0.272	0.350	3.2	4.3

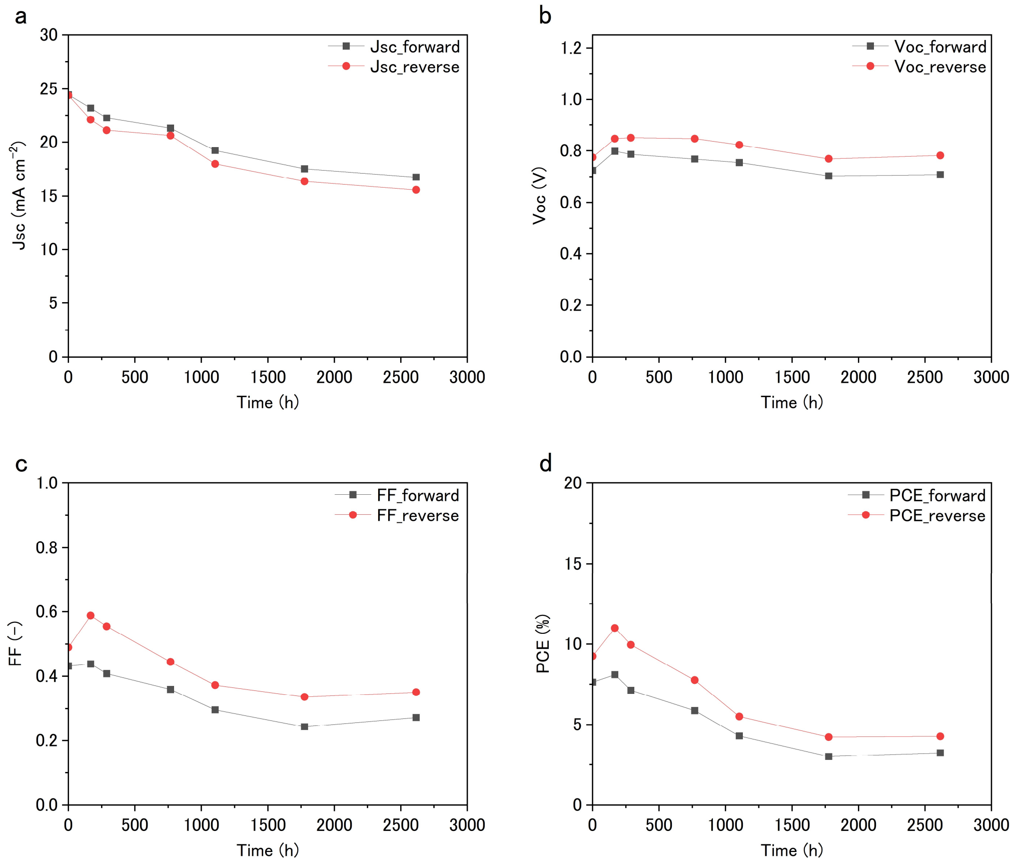

Supplementary Figure 23. Stability result of the perovskite solar cells with Li,Co+K11. (a) J_{SC}, (b) V_{OC}, (c) *FF*, and (d) *PCE* as a function of the time with forward and reverse scan. Sample was stored at 85 °C in dark.

Supplementary Table 25. Stability result of the perovskite solar cells with Li+K12. Sample was stored at 85 °C in dark.

Time (h)	Jsc (mA/sq) Forward	Jsc (mA/sq) Reverse	Voc (V) Forward	Voc (V) Reverse	FF (-) Forward	FF (-) Reverse	PCE (%) Forward	PCE (%) Reverse
0	24.40	24.41	0.754	0.807	0.449	0.549	8.3	10.8
168	24.38	24.22	0.772	0.788	0.289	0.366	5.4	7.0
312	24.39	24.32	0.774	0.794	0.327	0.439	6.2	8.5
792	24.30	24.29	0.774	0.806	0.336	0.456	6.3	8.9
1128	24.31	24.30	0.745	0.801	0.359	0.477	6.5	9.3
1704	24.03	23.97	0.755	0.815	0.341	0.441	6.2	8.6
2856	23.66	23.58	0.739	0.809	0.311	0.400	5.4	7.6

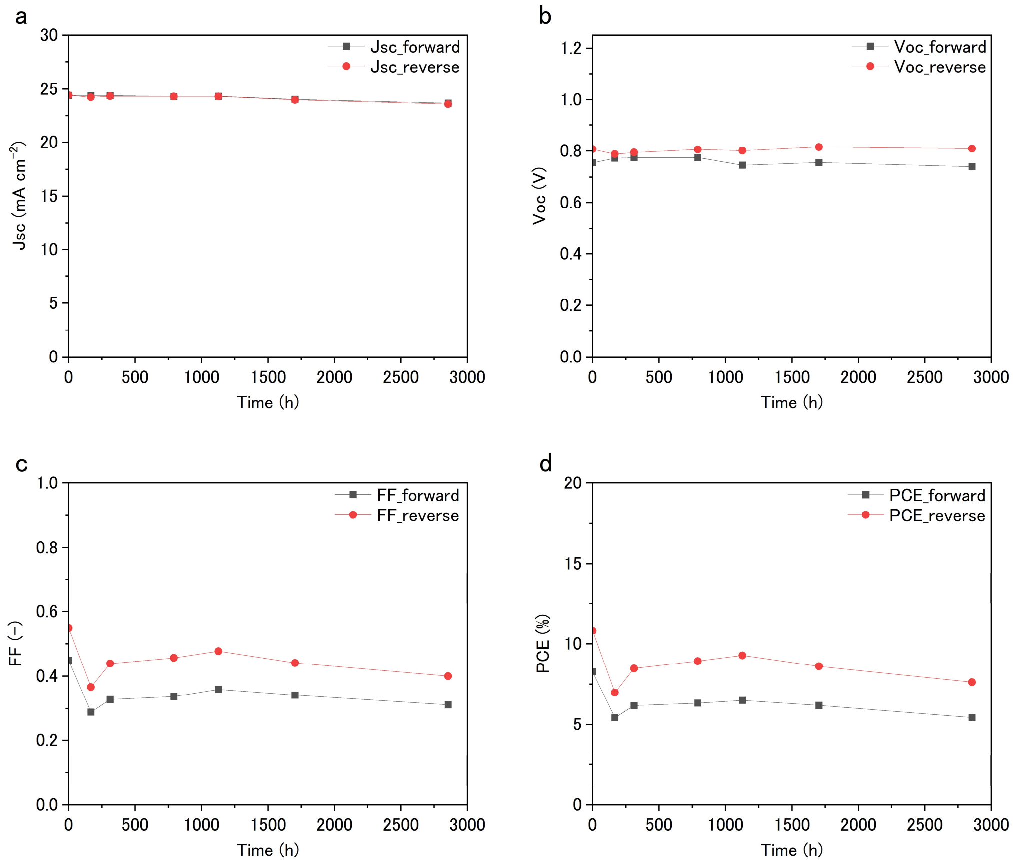

Supplementary Figure 24. Stability result of the perovskite solar cells with Li+K12. (a) J_{SC}, (b) V_{OC}, (c) *FF*, and (d) *PCE* as a function of the time with forward and reverse scan. Sample was stored at 85 °C in dark.

Supplementary Table 26. Stability result of the perovskite solar cells with Li,Co+K12. Sample was stored at 85 ℃ in dark.

Time (h)	Jsc (mA/sq) Forward	Jsc (mA/sq) Reverse	Voc (V) Forward	Voc (V) Reverse	FF (-) Forward	FF (-) Reverse	PCE (%) Forward	PCE (%) Reverse
0	24.40	24.39	0.796	0.853	0.509	0.599	9.9	12.5
168	24.39	24.36	0.855	0.863	0.492	0.612	10.3	12.9
288	23.64	23.59	0.836	0.863	0.469	0.602	9.3	12.3
768	24.28	24.29	0.791	0.860	0.447	0.533	8.6	11.1
1104	22.79	22.80	0.775	0.843	0.398	0.466	7.0	9.0
1776	24.07	24.01	0.762	0.815	0.340	0.397	6.2	7.8
2616	23.16	23.08	0.724	0.797	0.408	0.483	6.8	8.9

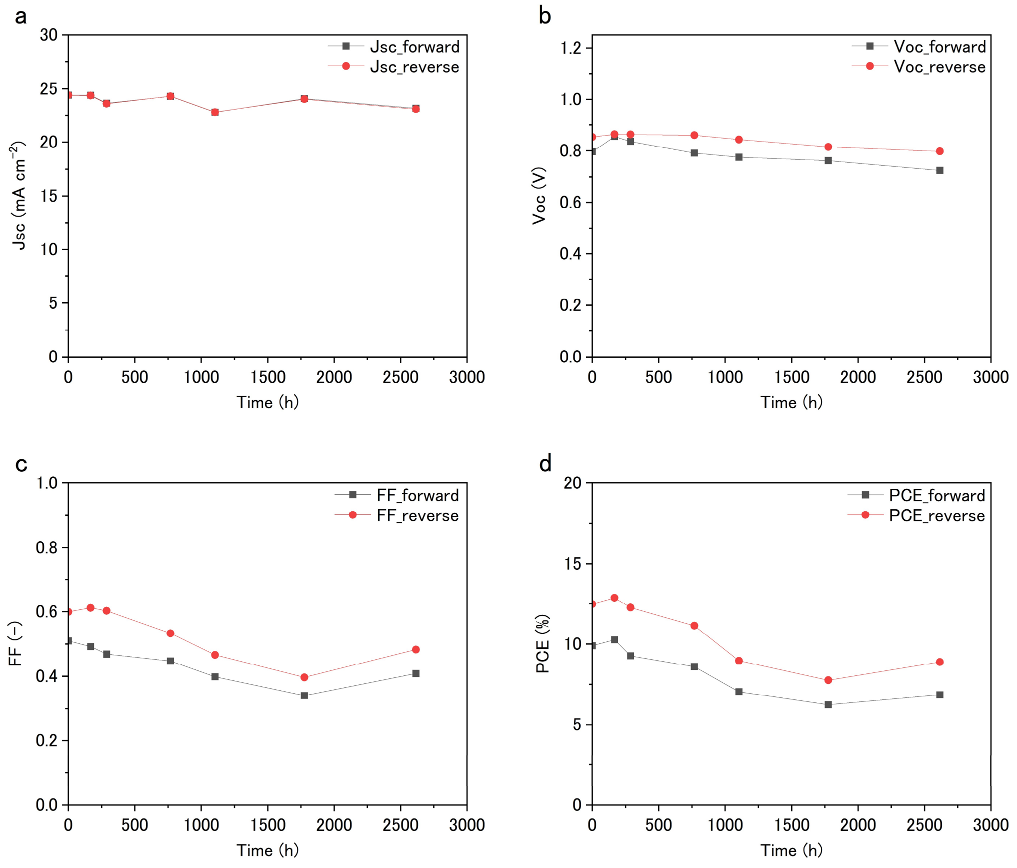

Supplementary Figure 25. Stability result of the perovskite solar cells with Li,Co+K12. (a) J_{SC}, (b) V_{OC}, (c) *FF*, and (d) *PCE* as a function of the time with forward and reverse scan. Sample was stored at 85 °C in dark.

Supplementary Table 27. Stability result of the perovskite solar cells with Li+K13. Sample was stored at 85 °C in dark.

Time (h)	Jsc (mA/sq) Forward	Jsc (mA/sq) Reverse	Voc (V) Forward	Voc (V) Reverse	FF (-) Forward	FF (-) Reverse	PCE (%) Forward	PCE (%) Reverse
0	24.71	24.70	0.787	0.832	0.479	0.588	9.3	12.1
168	24.76	24.72	0.854	0.882	0.523	0.695	11.1	15.1
312	24.61	24.65	0.856	0.889	0.522	0.682	11.0	15.0
792	24.46	24.43	0.825	0.884	0.515	0.661	10.4	14.3
1128	24.56	24.51	0.827	0.886	0.507	0.645	10.3	14.0
1704	24.38	24.38	0.817	0.884	0.474	0.583	9.4	12.6
2856	23.63	23.64	0.806	0.875	0.410	0.504	7.8	10.4

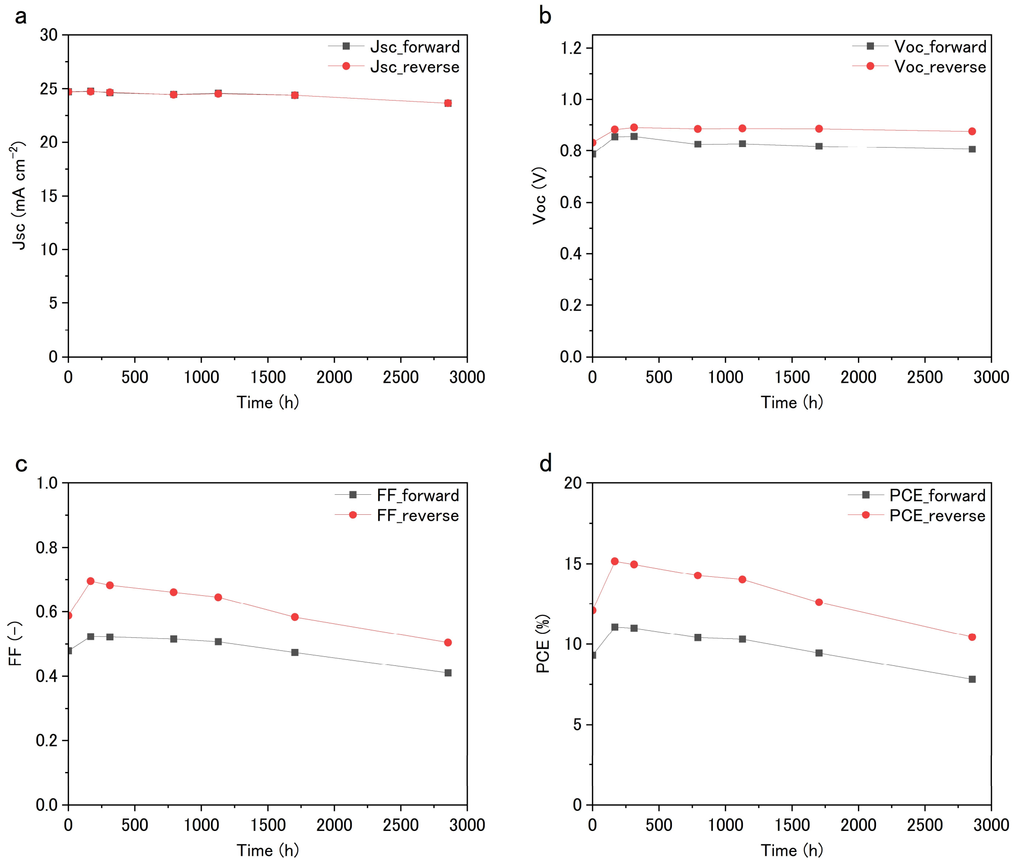

Supplementary Figure 26. Stability result of the perovskite solar cells with Li+K13. (a) J_{SC}, (b) V_{OC}, (c) *FF*, and (d) *PCE* as a function of the time with forward and reverse scan. Sample was stored at 85 °C in dark.

Supplementary Table 28. Stability result of the perovskite solar cells with Li,Co+K13. Sample was stored at 85 °C in dark.

Time (h)	Jsc (mA/sq) Forward	Jsc (mA/sq) Reverse	Voc (V) Forward	Voc (V) Reverse	FF (-) Forward	FF (-) Reverse	PCE (%) Forward	PCE (%) Reverse
0	24.70	24.53	0.825	0.900	0.411	0.482	8.4	10.6
168	24.90	23.78	0.687	0.774	0.469	0.614	8.0	11.3
288	24.42	23.11	0.616	0.689	0.458	0.577	6.9	9.2
768	24.54	24.22	0.827	0.878	0.423	0.510	8.6	10.9
1104	23.98	22.20	0.813	0.860	0.294	0.366	5.7	7.0
1776	23.03	21.55	0.775	0.848	0.270	0.345	4.8	6.3
2616	21.34	19.36	0.758	0.805	0.244	0.320	3.9	5.0

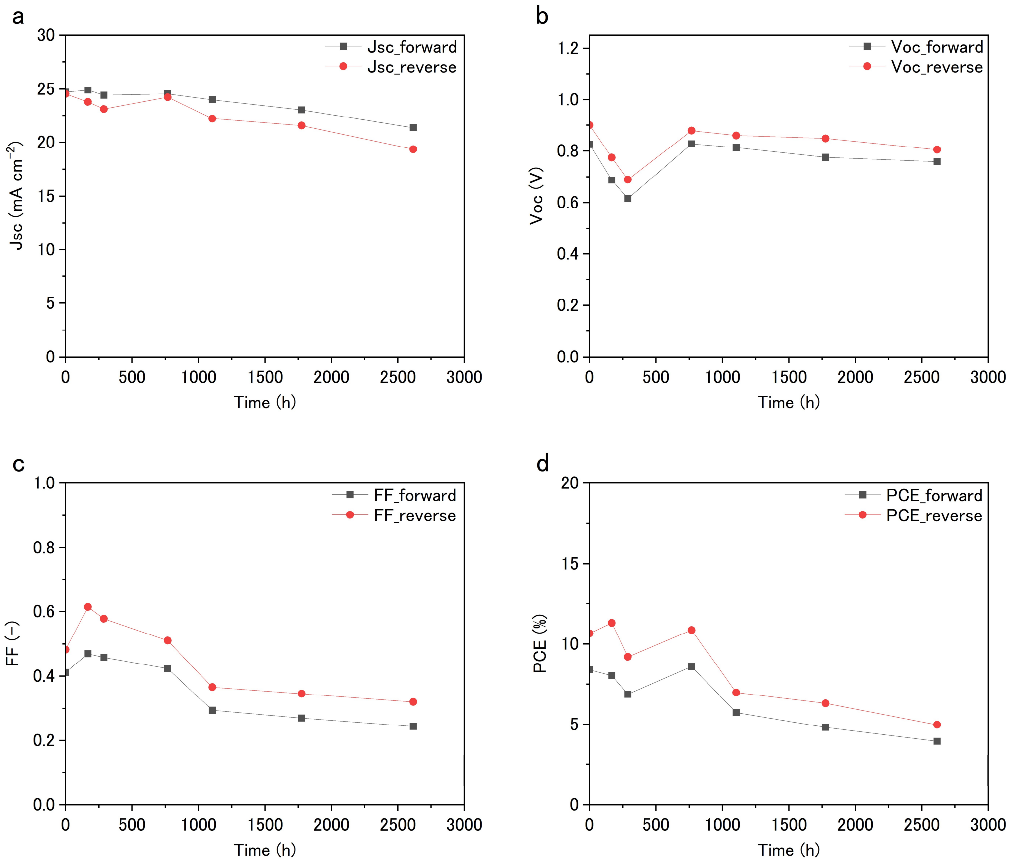

Supplementary Figure 27. Stability result of the perovskite solar cells with Li,Co+K13. (a) J_{SC}, (b) V_{OC}, (c) *FF*, and (d) *PCE* as a function of the time with forward and reverse scan. Sample was stored at 85 °C in dark.

Supplementary Table 29. Stability result of the perovskite solar cells with Li+K14. Sample was stored at 85 °C in dark.

Time (h)	Jsc (mA/sq) Forward	Jsc (mA/sq) Reverse	Voc (V) Forward	Voc (V) Reverse	FF (-) Forward	FF (-) Reverse	PCE (%) Forward	PCE (%) Reverse
0	24.74	24.69	0.804	0.873	0.529	0.673	10.5	14.5
168	24.63	24.65	0.874	0.897	0.497	0.696	10.7	15.4
312	24.48	24.49	0.875	0.909	0.497	0.674	10.6	15.0
792	24.43	24.45	0.845	0.907	0.508	0.632	10.5	14.0
1128	24.49	24.50	0.837	0.904	0.506	0.632	10.4	14.0
1704	24.46	24.46	0.825	0.895	0.463	0.567	9.3	12.4
2856	23.70	23.66	0.798	0.881	0.485	0.591	9.2	12.3

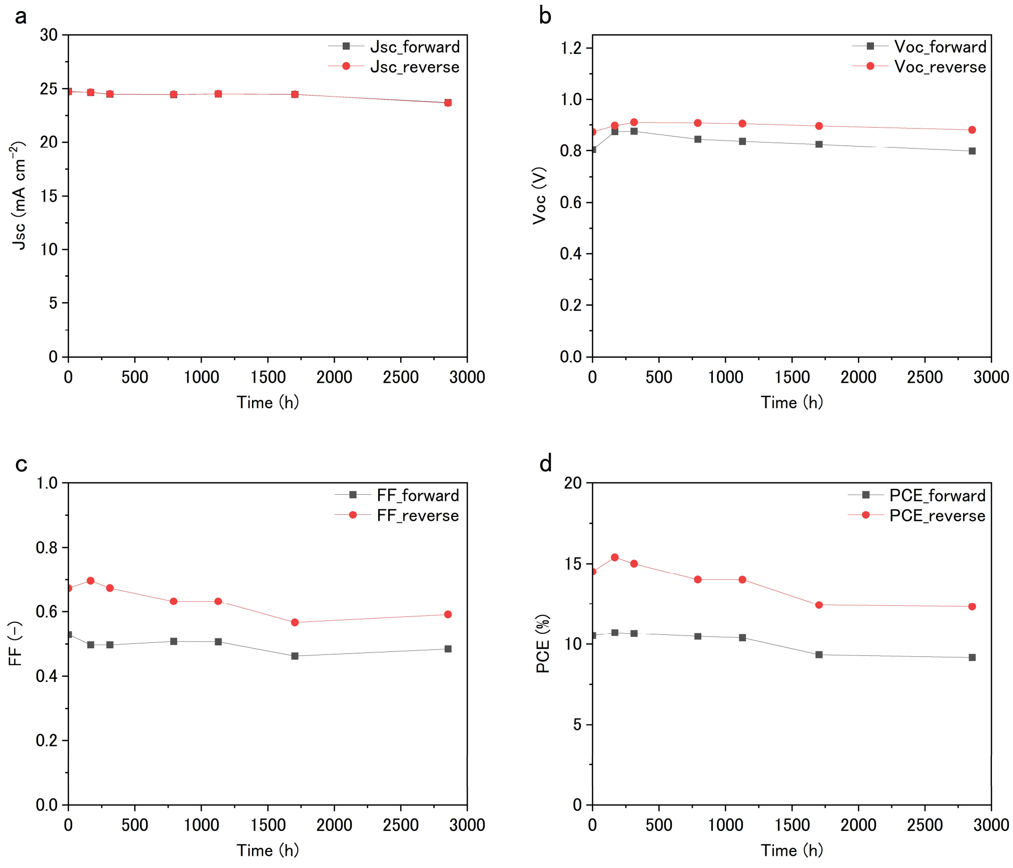

Supplementary Figure 28. Stability result of the perovskite solar cells with Li+K14. (a) J_{SC}, (b) V_{OC}, (c) *FF*, and (d) *PCE* as a function of the time with forward and reverse scan. Sample was stored at 85 °C in dark.

Supplementary Table 30. Stability result of the perovskite solar cells with Li,Co+K14. Sample was stored at 85 °C in dark.

Time (h)	Jsc (mA/sq) Forward	Jsc (mA/sq) Reverse	Voc (V) Forward	Voc (V) Reverse	FF (-) Forward	FF (-) Reverse	PCE (%) Forward	PCE (%) Reverse
0	24.77	24.81	0.897	0.947	0.549	0.633	12.2	14.9
168	24.79	24.85	0.885	0.892	0.539	0.666	11.8	14.8
288	24.81	24.83	0.863	0.881	0.510	0.667	10.9	14.6
768	22.04	22.05	0.849	0.905	0.505	0.618	9.5	12.3
1104	23.78	23.82	0.824	0.895	0.517	0.628	10.1	13.4
1776	24.47	24.53	0.805	0.888	0.517	0.614	10.2	13.4
2616	23.75	23.74	0.794	0.862	0.455	0.529	8.6	10.8

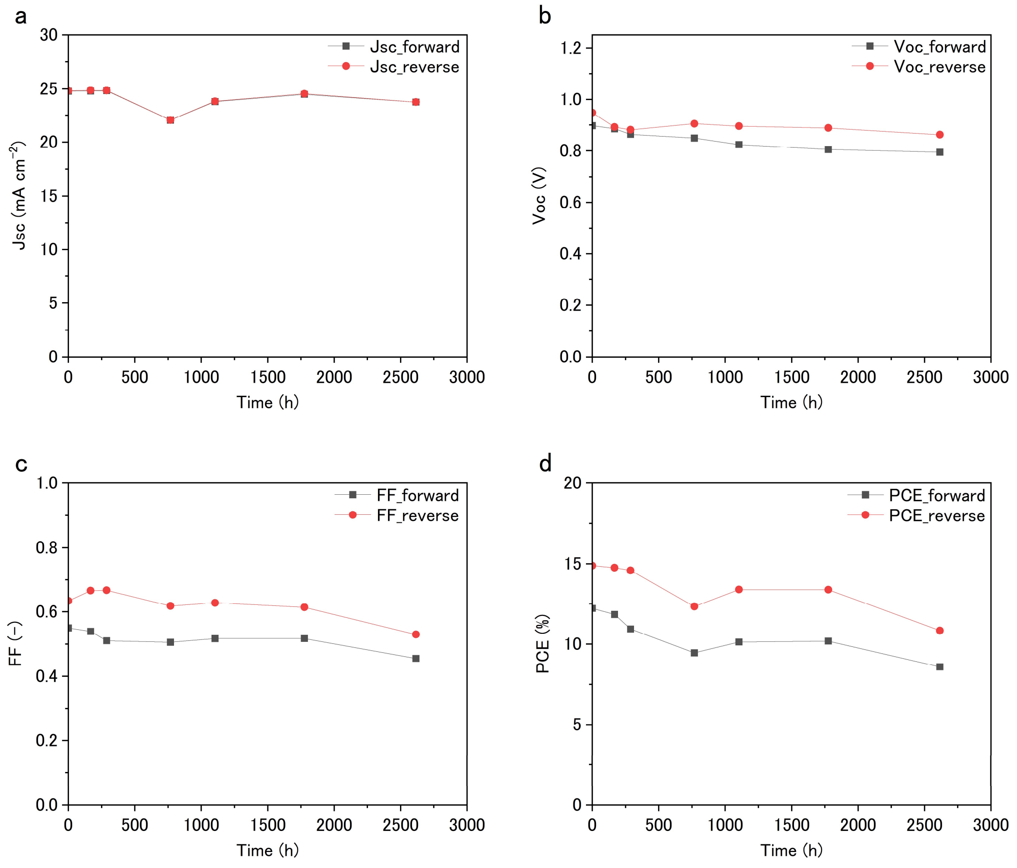

Supplementary Figure 29. Stability result of the perovskite solar cells with Li,Co+K14. (a) J_{SC}, (b) V_{OC}, (c) *FF*, and (d) *PCE* as a function of the time with forward and reverse scan. Sample was stored at 85 °C in dark.

Supplementary Table 31. Stability result of the perovskite solar cells with Li+K15. Sample was stored at 85 °C in dark.

Time (h)	Jsc (mA/sq) Forward	Jsc (mA/sq) Reverse	Voc (V) Forward	Voc (V) Reverse	FF (-) Forward	FF (-) Reverse	PCE (%) Forward	PCE (%) Reverse
0	24.65	24.60	0.732	0.787	0.362	0.529	6.5	10.2
72	24.64	24.56	0.847	0.859	0.348	0.466	7.3	9.8
168	24.82	24.84	0.836	0.861	0.426	0.588	8.8	12.6
336	24.93	24.85	0.846	0.866	0.339	0.473	7.2	10.2
504	24.92	24.94	0.838	0.865	0.457	0.609	9.6	13.1
624	24.95	24.95	0.842	0.860	0.408	0.529	8.6	11.4
1104	24.83	24.86	0.837	0.873	0.363	0.493	7.5	10.7
1440	24.63	24.65	0.842	0.874	0.396	0.493	8.2	10.6
1944	24.75	24.72	0.817	0.871	0.451	0.581	9.1	12.5
2784	23.92	23.84	0.817	0.862	0.358	0.435	7.0	8.9

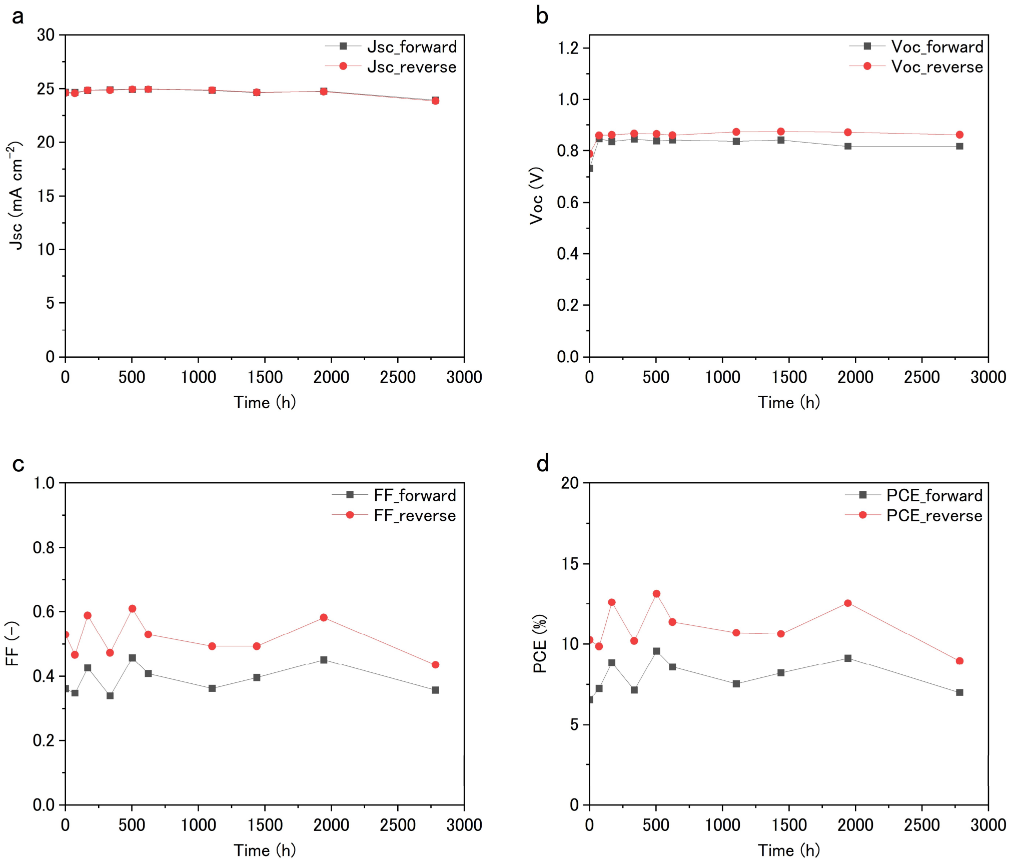

Supplementary Figure 30. Stability result of the perovskite solar cells with Li+K15. (a) J_{SC}, (b) V_{OC}, (c) *FF*, and (d) *PCE* as a function of the time with forward and reverse scan. Sample was stored at 85 °C in dark.

Supplementary Table 32. Stability result of the perovskite solar cells with Li,Co+K15. Sample was stored at 85 °C in dark.

	Jsc (mA/sq)	Jsc (mA/sq)	Voc (V)	Voc (V)	FF (-)	FF (-)	PCE (%)	PCE (%)
Time (h)	Forward	Reverse	Forward	Reverse	Forward	Reverse	Forward	Reverse
0	24.56	24.56	0.789	0.851	0.388	0.566	7.5	11.8
72	24.66	23.28	0.783	0.845	0.394	0.612	7.6	12.0
168	23.82	22.16	0.797	0.856	0.382	0.593	7.3	11.2
336	24.83	23.34	0.834	0.874	0.382	0.559	7.9	11.4
504	24.91	23.67	0.837	0.878	0.387	0.550	8.1	11.4
624	24.87	23.78	0.844	0.882	0.377	0.535	7.9	11.2
1440	24.14	23.11	0.835	0.883	0.289	0.370	5.8	7.6
1944	23.58	22.23	0.796	0.867	0.279	0.354	5.2	6.8
2784	20.20	18.33	0.786	0.839	0.232	0.293	3.7	4.5

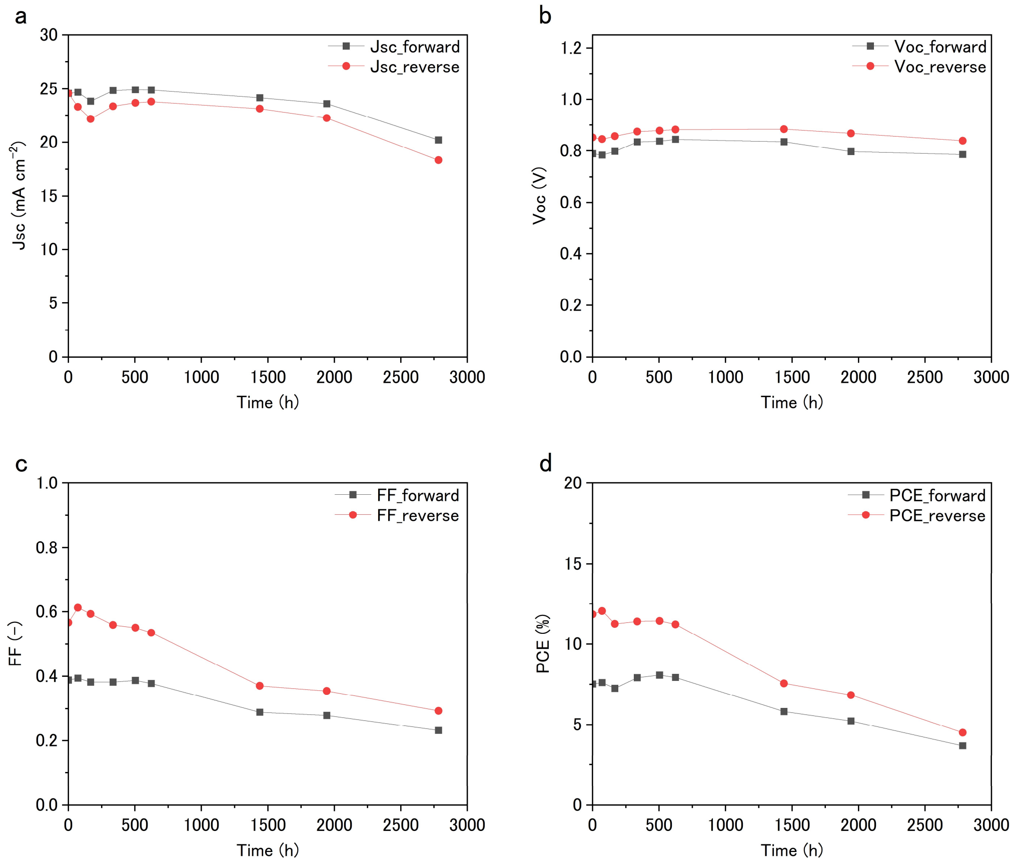

Supplementary Figure 31. Stability result of the perovskite solar cells with Li,Co+K15. (a) J_{SC}, (b) V_{OC}, (c) *FF*, and (d) *PCE* as a function of the time with forward and reverse scan. Sample was stored at 85 °C in dark.

Supplementary Table 33. Stability result of the perovskite solar cells with Li+K16. Sample was stored at 85 °C in dark.

	Jsc (mA/sq)	Jsc (mA/sq)	Voc (V)	Voc (V)	FF (-)	FF (-)	PCE (%)	PCE (%)
Time (h)	Forward	Reverse	Forward	Reverse	Forward	Reverse	Forward	Reverse
0	24.88	24.90	0.906	0.972	0.524	0.668	11.8	16.2
168	24.86	24.85	0.855	0.882	0.457	0.656	9.7	14.4
288	24.80	24.83	0.868	0.895	0.461	0.635	9.9	14.1
768	24.75	24.69	0.836	0.896	0.456	0.593	9.4	13.1
1104	24.67	24.60	0.814	0.887	0.475	0.611	9.5	13.3
1776	24.49	24.41	0.825	0.888	0.419	0.529	8.5	11.5
2616	23.65	23.47	0.782	0.854	0.386	0.468	7.1	9.4

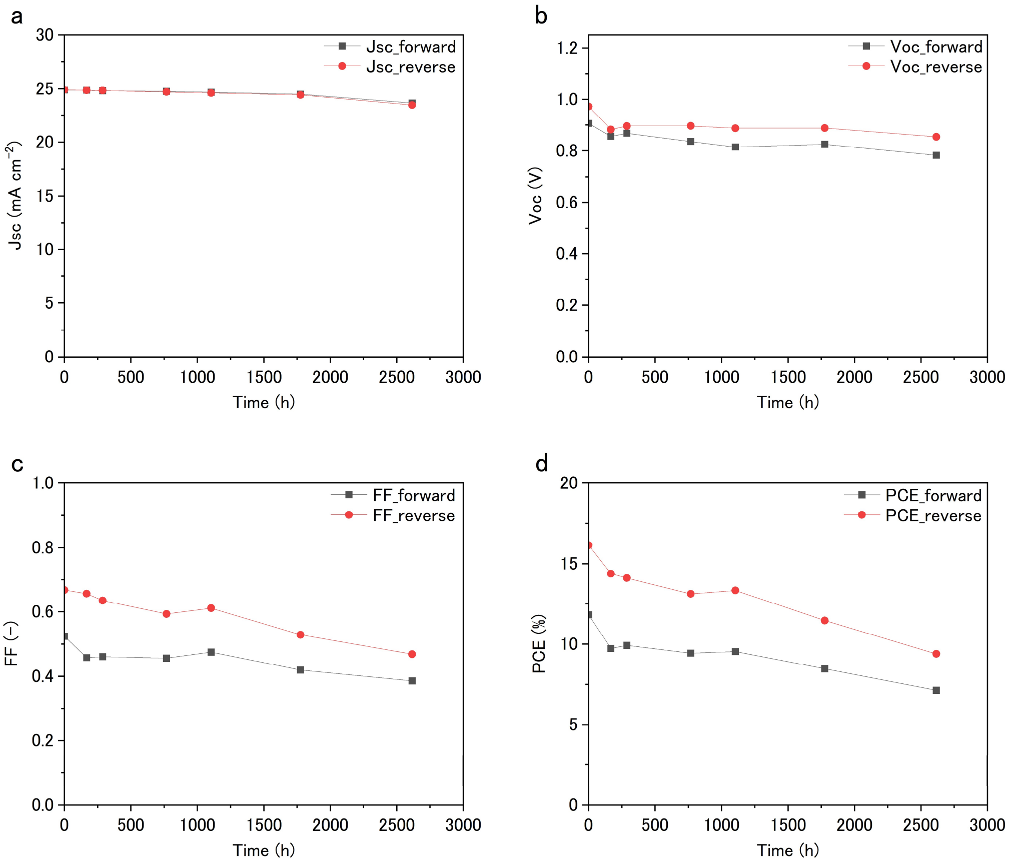

Supplementary Figure 32. Stability result of the perovskite solar cells with Li+K16. (a) J_{SC}, (b) V_{OC}, (c) *FF*, and (d) *PCE* as a function of the time with forward and reverse scan. Sample was stored at 85 °C in dark.

Supplementary Table 34. Stability result of the perovskite solar cells with Li+K17. Sample was stored at 85 °C in dark.

Time (h)	Jsc (mA/sq) Forward	Jsc (mA/sq) Reverse	Voc (V) Forward	Voc (V) Reverse	FF (-) Forward	FF (-) Reverse	PCE (%) Forward	PCE (%) Reverse
0	24.76	24.66	0.739	0.772	0.390	0.481	7.1	9.2
168	24.62	24.44	0.813	0.843	0.428	0.563	8.6	11.6
312	23.56	23.44	0.818	0.854	0.447	0.549	8.6	11.0
792	23.83	23.69	0.796	0.847	0.417	0.495	7.9	9.9
1128	23.07	22.82	0.765	0.827	0.377	0.447	6.6	8.4
1704	23.35	23.06	0.771	0.835	0.372	0.439	6.7	8.5
2856	23.04	22.74	0.757	0.831	0.392	0.472	6.8	8.9

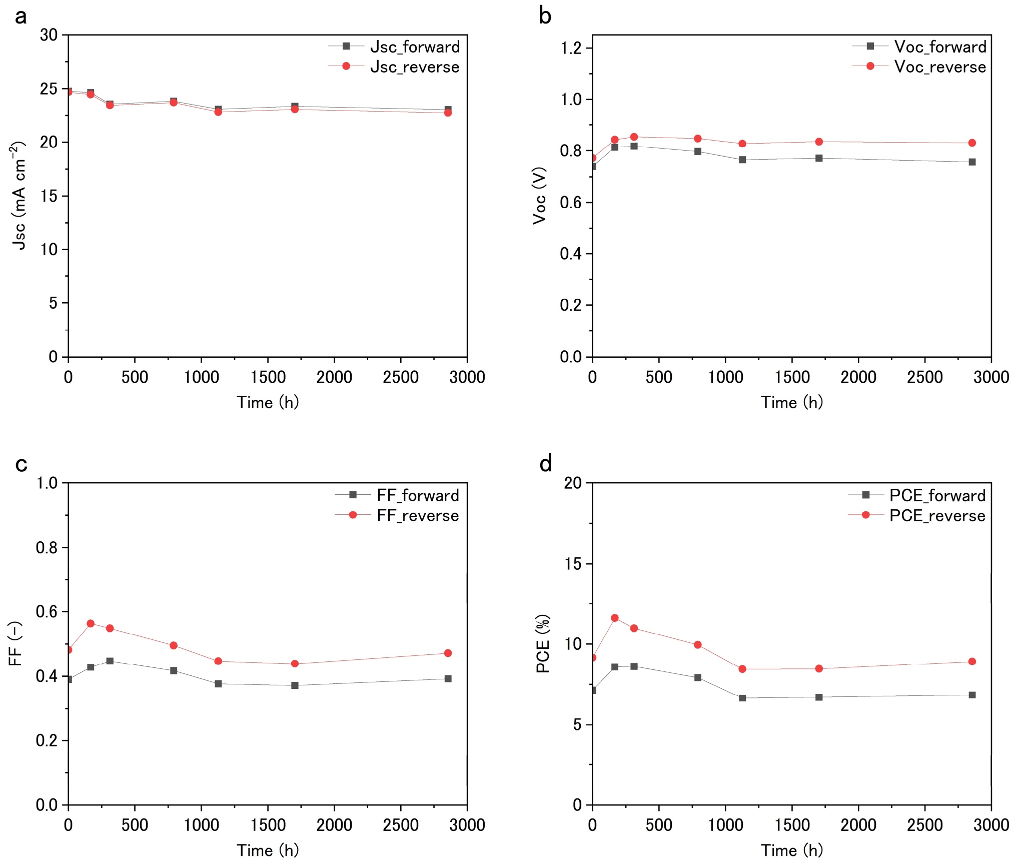

Supplementary Figure 33. Stability result of the perovskite solar cells with Li+K17. (a) J_{SC}, (b) V_{OC}, (c) *FF*, and (d) *PCE* as a function of the time with forward and reverse scan. Sample was stored at 85 °C in dark.

Supplementary Table 35. Stability result of the perovskite solar cells with Li,Co+K17. Sample was stored at 85 °C in dark.

Time (h)	Jsc (mA/sq) Forward	Jsc (mA/sq) Reverse	Voc (V) Forward	Voc (V) Reverse	FF (-) Forward	FF (-) Reverse	PCE (%) Forward	PCE (%) Reverse
0	24.35	23.86	0.797	0.870	0.399	0.513	7.7	10.6
168	21.52	18.32	0.754	0.828	0.392	0.592	6.4	9.0
288	23.97	19.43	0.754	0.819	0.350	0.550	6.3	8.7
768	18.28	14.20	0.743	0.758	0.249	0.389	3.4	4.2
1104	18.78	14.15	0.729	0.767	0.263	0.459	3.6	5.0
1776	20.65	16.57	0.751	0.814	0.221	0.359	3.4	4.8
2616	16.47	13.53	0.766	0.824	0.197	0.288	2.5	3.2

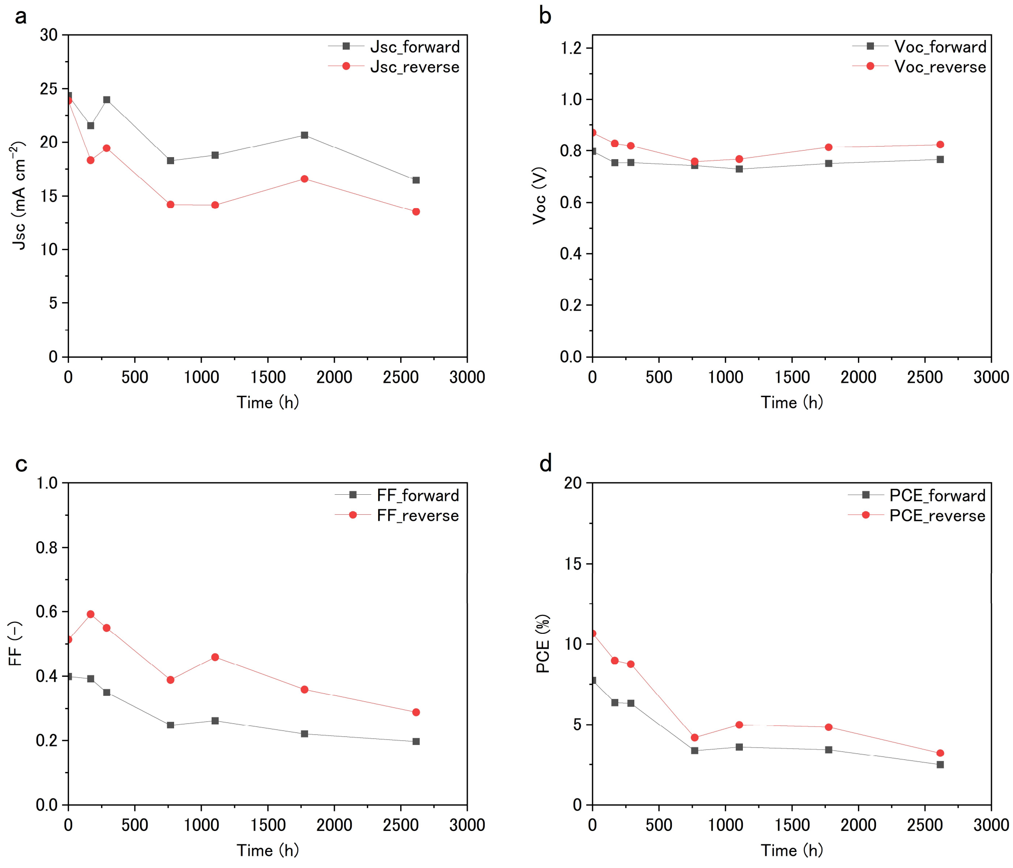

Supplementary Figure 34. Stability result of the perovskite solar cells with Li,Co+K17. (a) J_{SC}, (b) V_{OC}, (c) *FF*, and (d) *PCE* as a function of the time with forward and reverse scan. Sample was stored at 85 °C in dark.

Supplementary Table 36. Stability result of the perovskite solar cells with Li+K18. Sample was stored at 85 °C in dark.

Time (h)	Jsc (mA/sq) Forward	Jsc (mA/sq) Reverse	Voc (V) Forward	Voc (V) Reverse	FF (-) Forward	FF (-) Reverse	PCE (%) Forward	PCE (%) Reverse
0	10.99	10.61	0.955	0.955	0.282	0.298	3.0	3.0
96	1.52	1.45	1.007	1.009	0.238	0.258	0.4	0.4
408	1.55	1.48	1.007	1.010	0.238	0.259	0.4	0.4
816	0.33	0.32	0.978	0.979	0.206	0.221	0.1	0.1

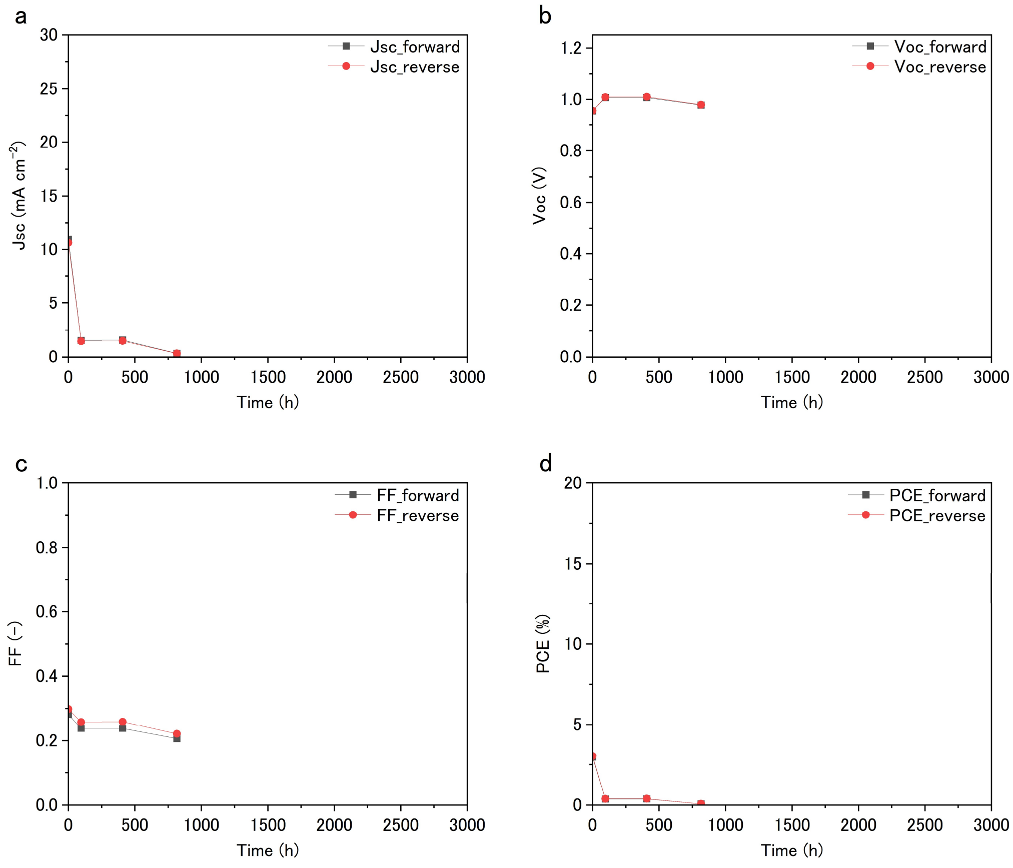

Supplementary Figure 35. Stability result of the perovskite solar cells with Li+K18. (a) J_{SC}, (b) V_{OC}, (c) FF, and (d) PCE as a function of the time with forward and reverse scan. Sample was stored at 85 °C in dark.

Supplementary Table 37. Photovoltaic properties of the perovskite solar cells with Li+K19.

	Jsc (mA/sq)	Jsc (mA/sq)	Voc (V)	Voc (V)	FF (-)	FF (-)	PCE (%)	PCE (%)
Time (h)	Forward	Reverse	Forward	Reverse	Forward	Reverse	Forward	Reverse
0	0.14	0.14	0.960	0.958	0.227	0.231	0.0	0.0

Supplementary Table 38. Photovoltaic properties of the perovskite solar cells with Li+K20.

	Jsc (mA/sq)	Jsc (mA/sq)	Voc (V)	Voc (V)	FF (-)	FF (-)	PCE (%)	PCE (%)
Time (h)	Forward	Reverse	Forward	Reverse	Forward	Reverse	Forward	Reverse
0	0.00	0.01	0.946	0.944	0.184	0.188	0.0	0.0

Supplementary Table 39. Stability result of the perovskite solar cells with Li+K21. Sample was stored at 85 ℃ in dark.

Time (h)	Jsc (mA/sq) Forward	Jsc (mA/sq) Reverse	Voc (V) Forward	Voc (V) Reverse	FF (-) Forward	FF (-) Reverse	PCE (%) Forward	PCE (%) Reverse
0	24.80	24.78	0.795	0.869	0.432	0.605	8.5	13.0
72	5.62	5.51	0.861	0.861	0.198	0.208	1.0	1.0

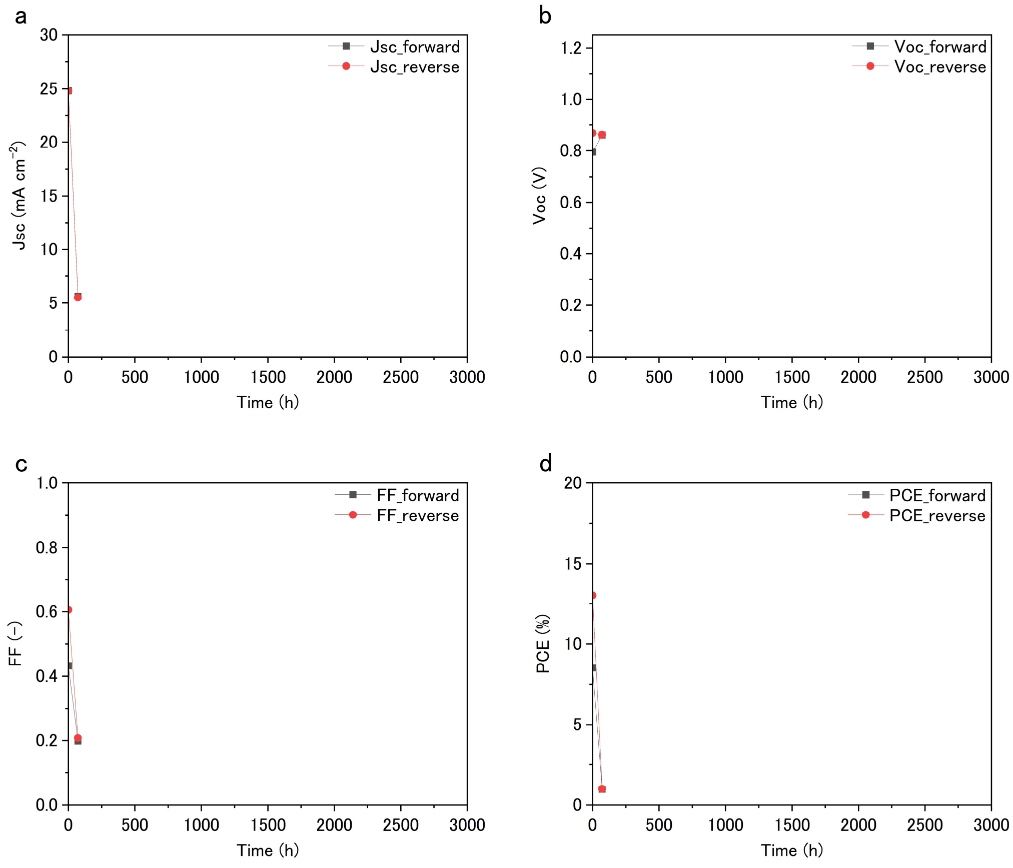

Supplementary Figure 36. Stability result of the perovskite solar cells with Li+K21. (a) J_{SC}, (b) V_{OC}, (c) *FF*, and (d) *PCE* as a function of the time with forward and reverse scan. Sample was stored at 85 °C in dark.

Supplementary Table 40. Stability result of the perovskite solar cells with Li,Co+K21. Sample was stored at 85 °C in dark.

Time (h)	Jsc (mA/sq) Forward	Jsc (mA/sq) Reverse	Voc (V) Forward	Voc (V) Reverse	FF (-) Forward	FF (-) Reverse	PCE (%) Forward	PCE (%) Reverse
0	25.48	25.49	0.917	0.984	0.416	0.532	9.7	13.3
72	2.34	2.20	0.859	0.852	0.265	0.299	0.5	0.6

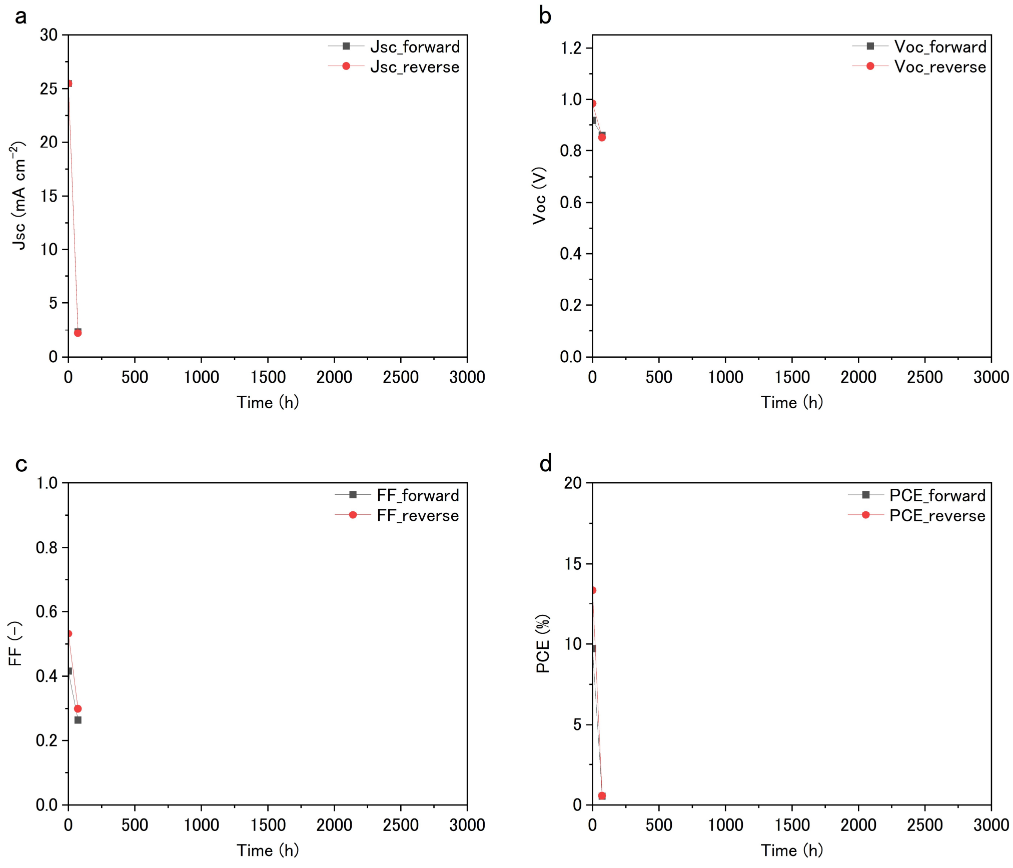

Supplementary Figure 37. Stability result of the perovskite solar cells with Li,Co+K21. (a) J_{SC}, (b) V_{OC}, (c) FF, and (d) PCE as a function of the time with forward and reverse scan. Sample was stored at 85 °C in dark.

Supplementary Table 41. Stability result of the perovskite solar cells with Li+K22. Sample was stored at 85 °C in dark.

Time (h)	Jsc (mA/sq) Forward	Jsc (mA/sq) Reverse	Voc (V) Forward	Voc (V) Reverse	FF (-) Forward	FF (-) Reverse	PCE (%) Forward	PCE (%) Reverse
0	24.63	24.63	0.795	0.828	0.440	0.481	8.6	9.8
168	24.27	20.16	0.836	0.839	0.261	0.373	5.3	6.3
312	17.64	16.07	0.849	0.838	0.275	0.256	4.1	3.5
792	6.55	5.76	0.672	0.623	0.179	0.190	0.8	0.7
1128	4.87	5.10	0.685	0.659	0.246	0.201	0.8	0.7
1704	2.01	2.10	0.659	0.629	0.278	0.219	0.4	0.3
2856	1.08	0.93	0.677	0.652	0.256	0.257	0.2	0.2

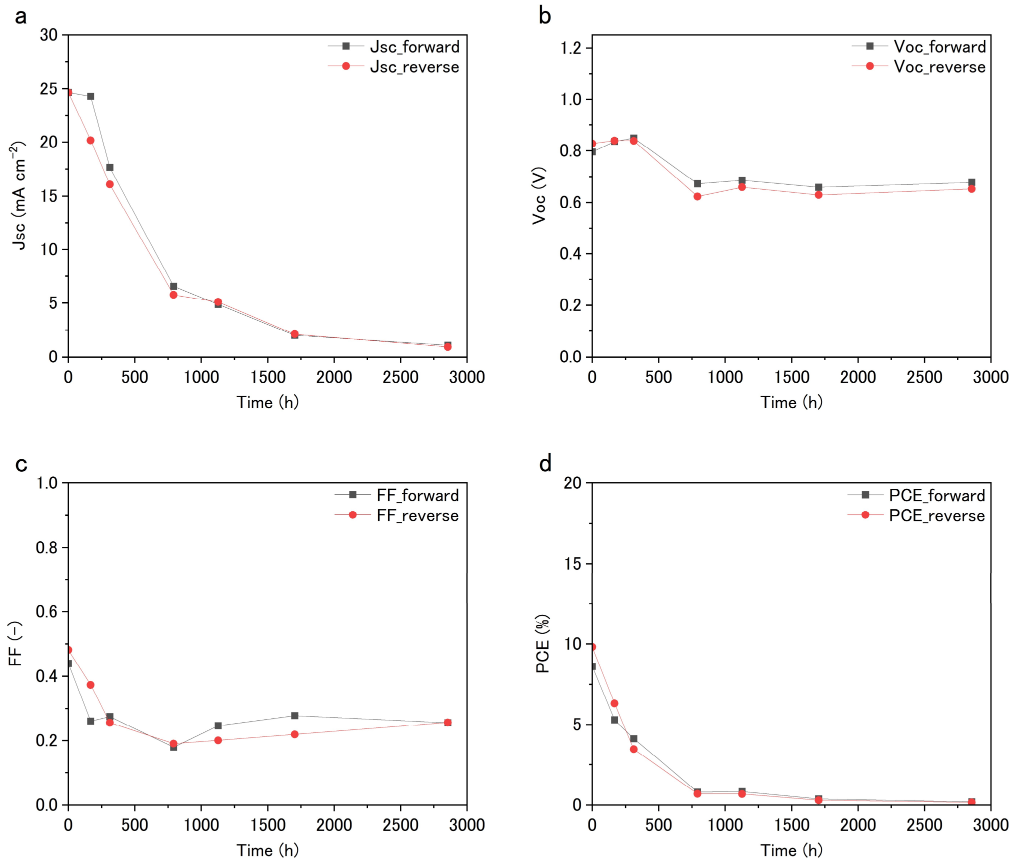

Supplementary Figure 38. Stability result of the perovskite solar cells with Li+K22. (a) J_{SC}, (b) V_{OC}, (c) FF, and (d) PCE as a function of the time with forward and reverse scan. Sample was stored at 85 °C in dark.

Supplementary Table 42. Stability result of the perovskite solar cells with Li,Co+K22. Sample was stored at 85 °C in dark.

	Jsc (mA/sq)	Jsc (mA/sq)	Voc (V)	Voc (V)	FF (-)	FF (-)	PCE (%)	PCE (%)
Time (h)	Forward	Reverse	Forward	Reverse	Forward	Reverse	Forward	Reverse
0	24.81	24.79	0.835	0.887	0.500	0.563	10.4	12.4
168	24.71	24.62	0.836	0.839	0.431	0.555	8.9	11.5
288	24.60	24.37	0.809	0.832	0.375	0.481	7.5	9.8
768	23.10	21.30	0.770	0.814	0.254	0.321	4.5	5.6
1104	23.66	22.66	0.735	0.811	0.288	0.363	5.0	6.7
1776	21.52	19.33	0.727	0.789	0.248	0.312	3.9	4.8
2616	18.26	16.18	0.713	0.770	0.224	0.282	2.9	3.5

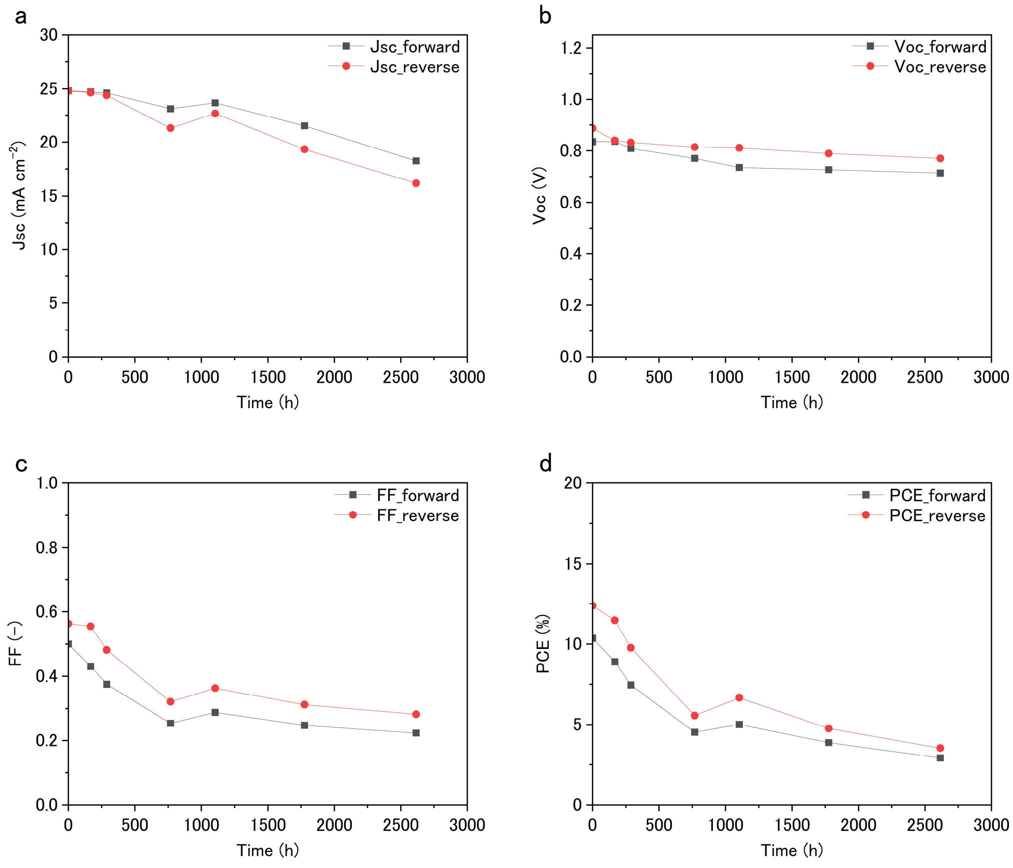

Supplementary Figure 39. Stability result of the perovskite solar cells with Li,Co+K22. (a) J_{SC}, (b) V_{OC}, (c) *FF*, and (d) *PCE* as a function of the time with forward and reverse scan. Sample was stored at 85 °C in dark.

Supplementary Table 43. Stability result of the perovskite solar cells with Li+K23. Sample was stored at 85 °C in dark.

Time (h)	Jsc (mA/sq) Forward	Jsc (mA/sq) Reverse	Voc (V) Forward	Voc (V) Reverse	FF (-) Forward	FF (-) Reverse	PCE (%) Forward	PCE (%) Reverse
0	24.81	23.91	1.076	1.096	0.394	0.508	10.5	13.3
168	11.13	6.60	1.052	1.052	0.191	0.356	2.2	2.5

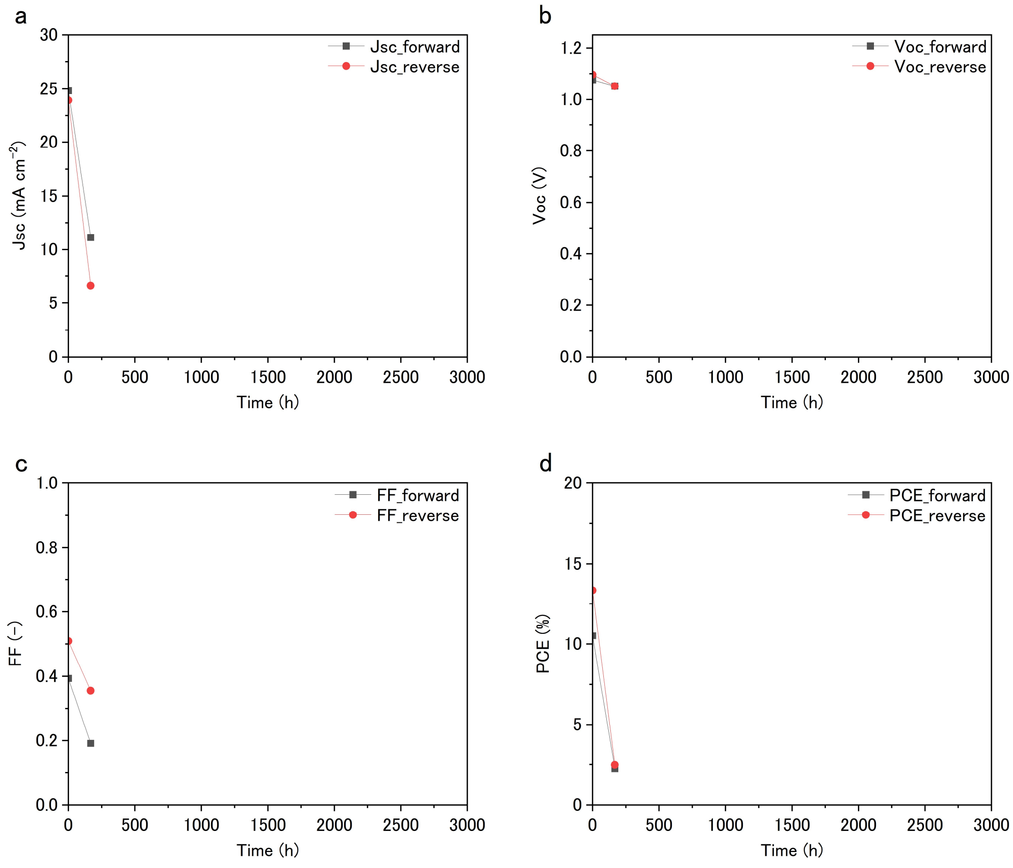

Supplementary Figure 40. Stability result of the perovskite solar cells with Li+K23. (a) J_{SC}, (b) V_{OC}, (c) FF, and (d) PCE as a function of the time with forward and reverse scan. Sample was stored at 85 °C in dark.

Supplementary Table 44. Stability result of the perovskite solar cells with Li,Co+K23. Sample was stored at 85 °C in dark.

Time (h)	Jsc (mA/sq) Forward	Jsc (mA/sq) Reverse	Voc (V) Forward	Voc (V) Reverse	FF (-) Forward	FF (-) Reverse	PCE (%) Forward	PCE (%) Reverse
0	24.81	24.73	1.021	1.042	0.447	0.559	11.3	14.4
168	2.70	1.92	0.917	0.930	0.198	0.315	0.5	0.6

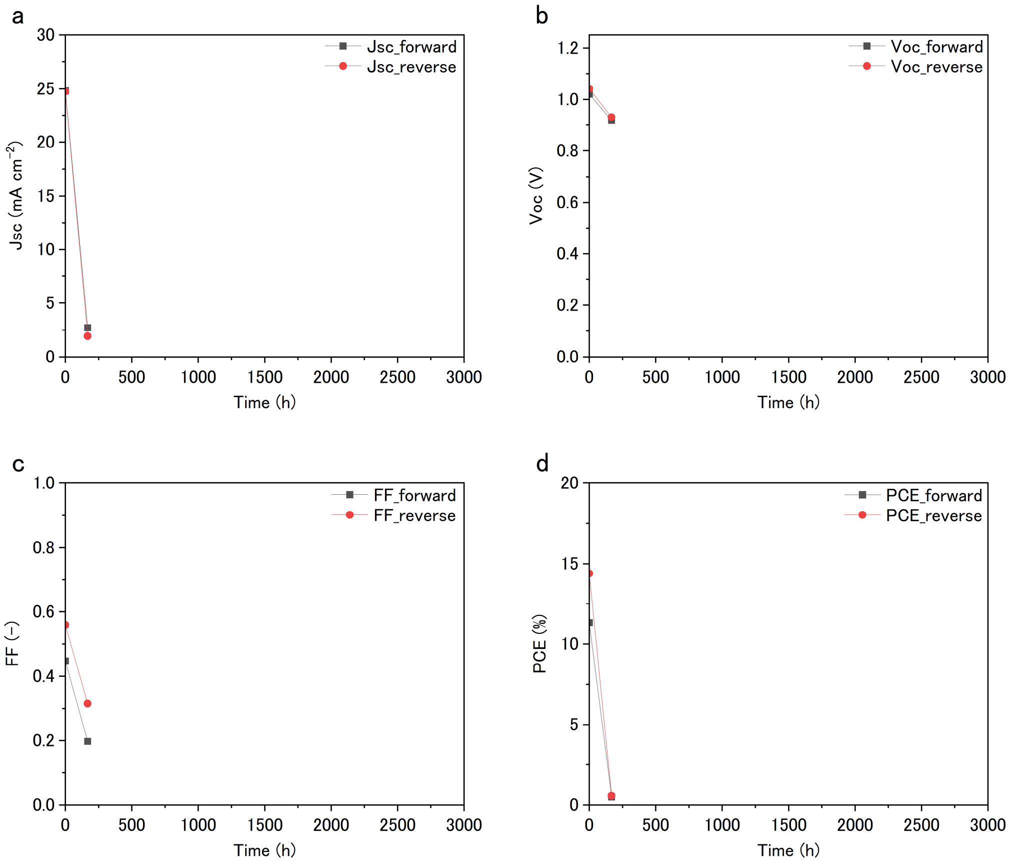

Supplementary Figure 41. Stability result of the perovskite solar cells with Li,Co+K23. (a) J_{SC}, (b) V_{OC}, (c) *FF*, and (d) *PCE* as a function of the time with forward and reverse scan. Sample was stored at 85 °C in dark.

Supplementary Table 45. Stability result of the perovskite solar cells with Li+K24. Sample was stored at 85 °C in dark.

Time (h)	Jsc (mA/sq) Forward	Jsc (mA/sq) Reverse	Voc (V) Forward	Voc (V) Reverse	FF (-) Forward	FF (-) Reverse	PCE (%) Forward	PCE (%) Reverse
0	24.15	24.10	0.818	0.867	0.437	0.491	8.6	10.3
168	5.08	5.06	0.817	0.819	0.227	0.234	0.9	1.0
336	6.09	6.08	0.819	0.823	0.233	0.241	1.2	1.2
504	4.23	4.25	0.803	0.807	0.225	0.229	0.8	0.8

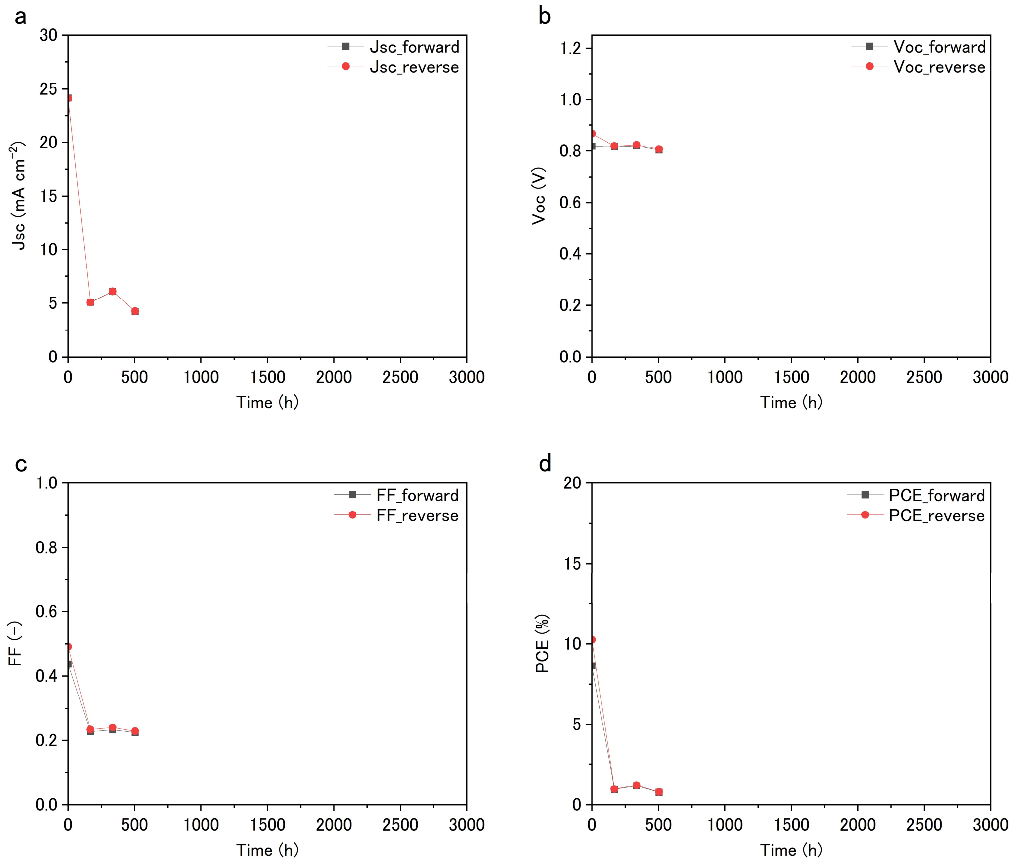

Supplementary Figure 42. Stability result of the perovskite solar cells with Li+K24. (a) J_{SC}, (b) V_{OC}, (c) *FF*, and (d) PCE as a function of the time with forward and reverse scan. Sample was stored at 85 °C in dark.

Supplementary Table 46. Stability result of the perovskite solar cells with Li,Co+K24. . Sample was stored at 85 ℃ in dark.

Time (h)	Jsc (mA/sq) Forward	Jsc (mA/sq) Reverse	Voc (V) Forward	Voc (V) Reverse	FF (-) Forward	FF (-) Reverse	PCE (%) Forward	PCE (%) Reverse
0	25.40	25.37	0.918	0.925	0.422	0.629	9.9	14.8
72	7.74	7.73	0.745	0.786	0.291	0.289	1.7	1.8

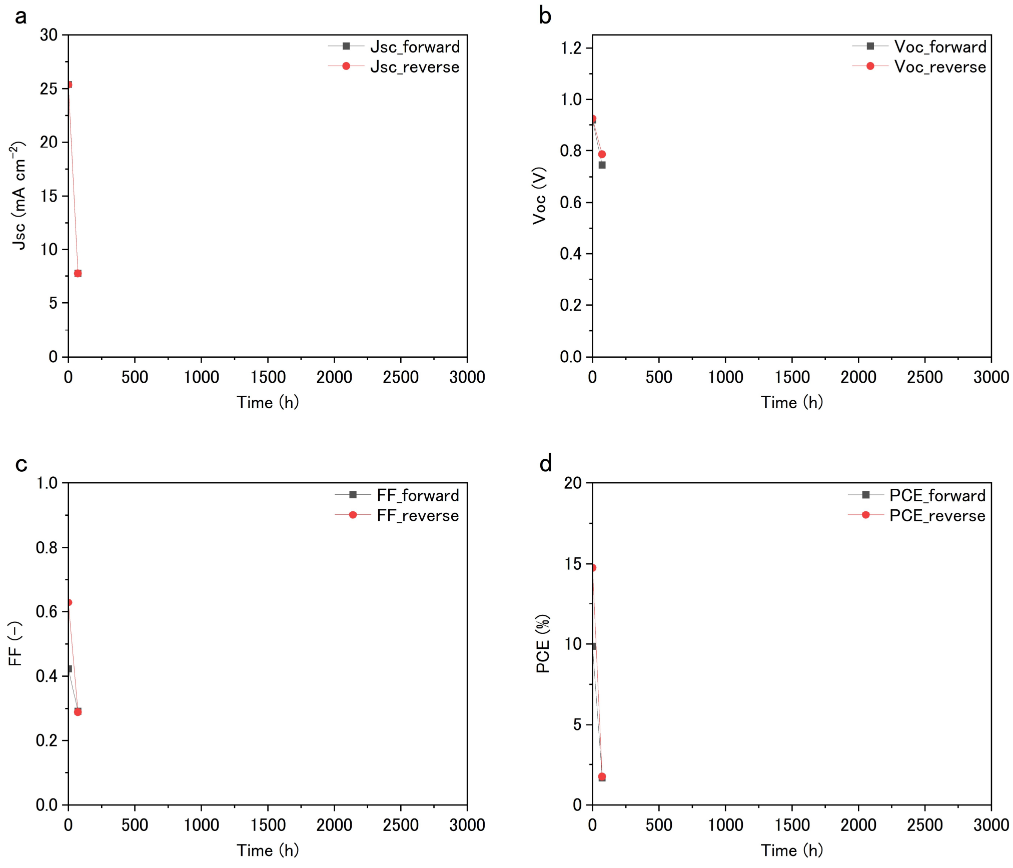

Supplementary Figure 43. Stability result of the perovskite solar cells with Li,Co+K24. (a) J_{SC}, (b) V_{OC}, (c) *FF*, and (d) *PCE* as a function of the time with forward and reverse scan. Sample was stored at 85 °C in dark.

Supplementary Table 47. Stability result of the perovskite solar cells with Li+K25. Sample was stored at 85 °C in dark.

Time (h)	Jsc (mA/sq) Forward	Jsc (mA/sq) Reverse	Voc (V) Forward	Voc (V) Reverse	FF (-) Forward	FF (-) Reverse	PCE (%) Forward	PCE (%) Reverse
0	0.03	0.01	0.815	0.957	0.074	0.513	0.0	0.0
168	0.00	0.00	0.030	0.037	0.248	0.249	0.0	0.0
312	0.00	0.00	0.029	0.055	0.255	0.250	0.0	0.0

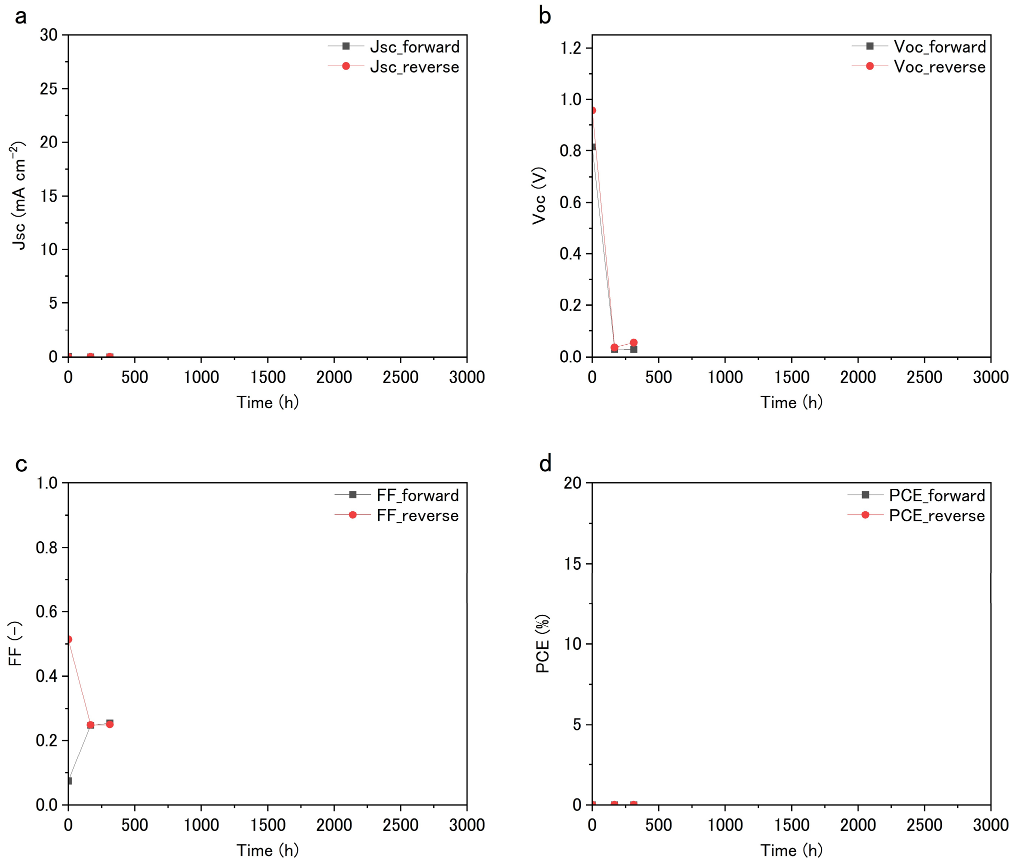

Supplementary Figure 44. Stability result of the perovskite solar cells with Li+K25. (a) J_{SC}, (b) V_{OC}, (c) FF, and (d) PCE as a function of the time with forward and reverse scan. Sample was stored at 85 °C in dark.

Supplementary Table 48. Stability result of the perovskite solar cells with Li+K26. Sample was stored at 85 °C in dark. The photovoltaic properties were too low.

Time (h)	Jsc (mA/sq) Forward	Jsc (mA/sq) Reverse	Voc (V) Forward	Voc (V) Reverse	FF (-) Forward	FF (-) Reverse	PCE (%) Forward	PCE (%) Reverse
0	-	-	-	-	-	-	-	-

Supplementary Table 49. Stability result of the perovskite solar cells with Li+K27. Sample was stored at 85 °C in dark.

Time (h)	Jsc (mA/sq) Forward	Jsc (mA/sq) Reverse	Voc (V) Forward	Voc (V) Reverse	FF (-) Forward	FF (-) Reverse	PCE (%) Forward	PCE (%) Reverse
0	24.82	24.79	0.916	0.961	0.525	0.749	11.9	17.8
72	24.74	24.77	0.900	0.918	0.607	0.762	13.5	17.3
168	24.86	24.86	0.915	0.935	0.629	0.765	14.3	17.8
336	24.72	24.72	0.915	0.946	0.609	0.757	13.8	17.7
504	24.60	24.59	0.917	0.945	0.595	0.738	13.4	17.2
624	24.77	24.77	0.915	0.942	0.561	0.699	12.7	16.3
1104	24.66	24.65	0.866	0.925	0.548	0.676	11.7	15.4
1440	24.57	24.55	0.859	0.915	0.489	0.609	10.3	13.7
1944	24.53	24.54	0.817	0.900	0.477	0.590	9.6	13.0
2784	23.85	23.62	0.805	0.880	0.401	0.469	7.7	9.7

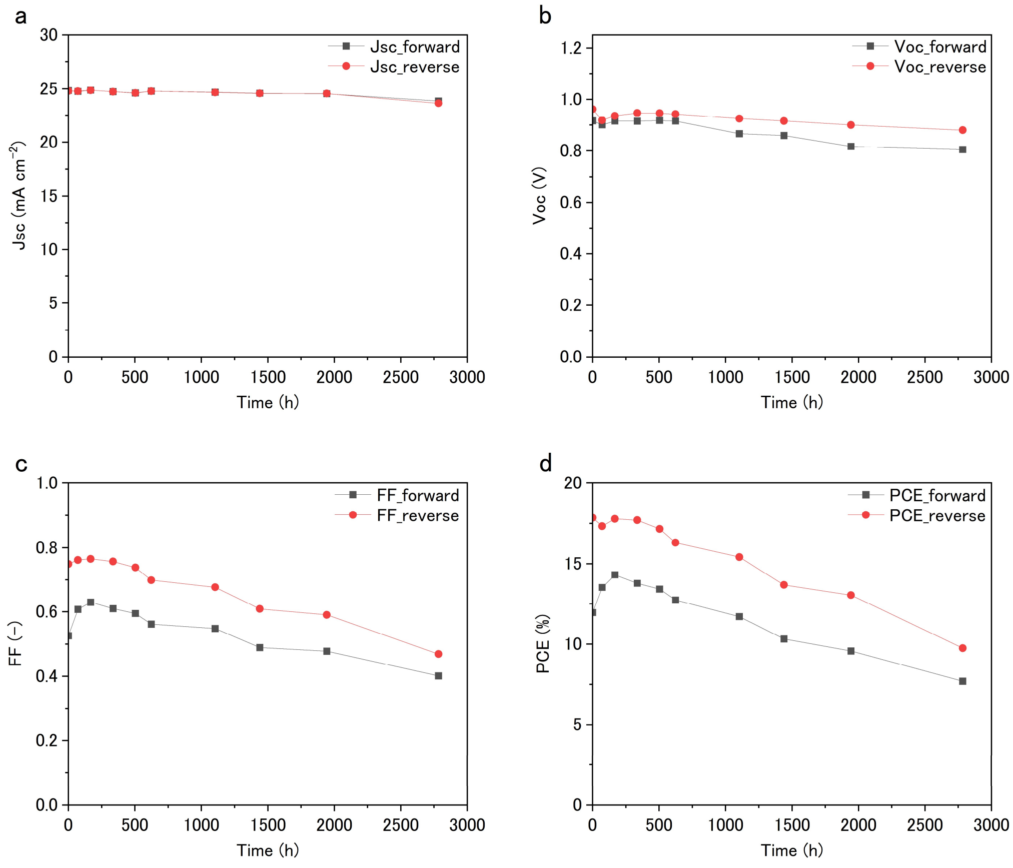

Supplementary Figure 45. Stability result of the perovskite solar cells with Li+K27. (a) J_{SC}, (b) V_{OC}, (c) *FF*, and (d) *PCE* as a function of the time with forward and reverse scan. Sample was stored at 85 °C in dark.

Supplementary Table 50. Stability result of the perovskite solar cells with Li,Co+K27. Sample was stored at 85 °C in dark.

Time (h)	Jsc (mA/sq) Forward	Jsc (mA/sq) Reverse	Voc (V) Forward	Voc (V) Reverse	FF (-) Forward	FF (-) Reverse	PCE (%) Forward	PCE (%) Reverse
0	24.52	24.53	1.025	1.054	0.495	0.745	12.5	19.3
72	2.81	1.99	0.921	0.895	0.171	0.302	0.4	0.5

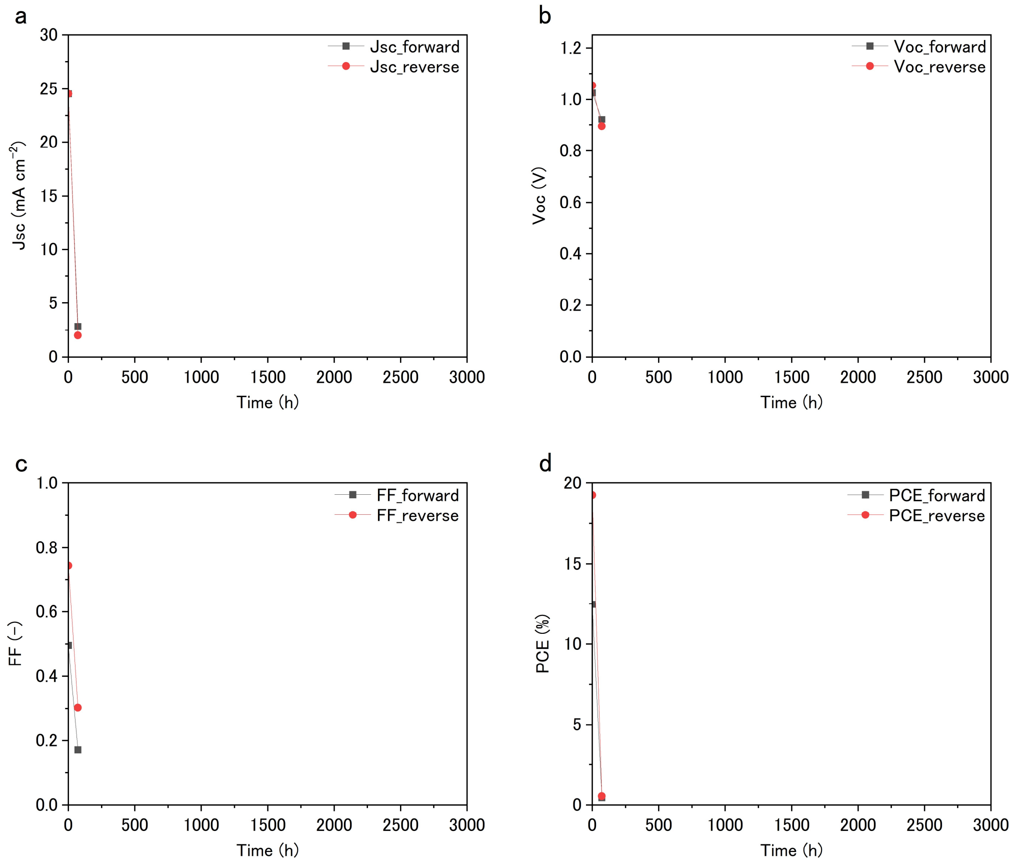

Supplementary Figure 46. Stability result of the perovskite solar cells with Li,Co+K27. (a) J_{SC}, (b) V_{OC}, (c) *FF*, and (d) *PCE* as a function of the time with forward and reverse scan. Sample was stored at 85 °C in dark.

Supplementary Table 51. Stability result of the perovskite solar cells with Li+K28. Sample was stored at 85 °C in dark.

Time (h)	Jsc (mA/sq) Forward	Jsc (mA/sq) Reverse	Voc (V) Forward	Voc (V) Reverse	FF (-) Forward	FF (-) Reverse	PCE (%) Forward	PCE (%) Reverse
0	24.95	24.96	0.827	0.900	0.467	0.621	9.6	13.9
96	24.40	24.40	0.843	0.865	0.526	0.697	10.8	14.7
408	24.90	24.89	0.886	0.905	0.483	0.648	10.7	14.6
816	24.87	24.86	0.885	0.915	0.538	0.664	11.8	15.1
1296	24.76	24.76	0.862	0.916	0.534	0.652	11.4	14.8
2424	24.14	24.15	0.846	0.911	0.538	0.638	11.0	14.0

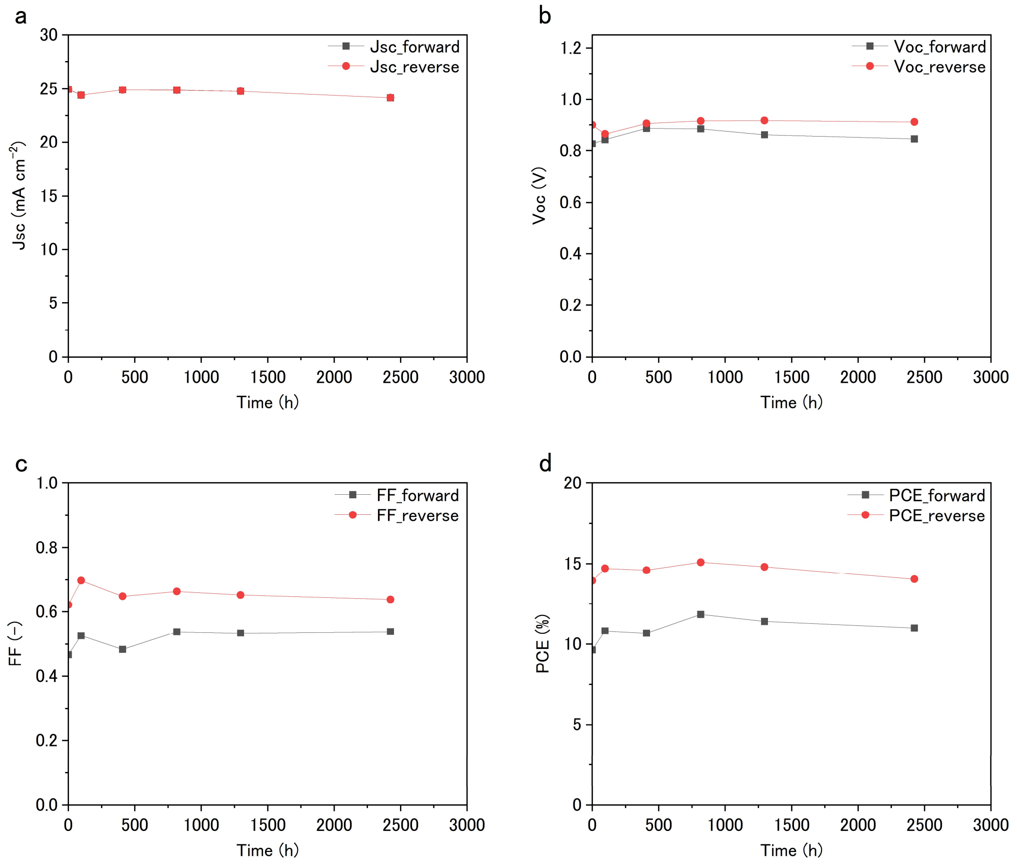

Supplementary Figure 47. Stability result of the perovskite solar cells with Li+K28. (a) J_{SC}, (b) V_{OC}, (c) *FF*, and (d) *PCE* as a function of the time with forward and reverse scan. Sample was stored at 85 °C in dark.

Supplementary Table 52. Stability result of the perovskite solar cells with Li+K29. Sample was stored at 85 ℃ in dark.

Time (h)	Jsc (mA/sq) Forward	Jsc (mA/sq) Reverse	Voc (V) Forward	Voc (V) Reverse	FF (-) Forward	FF (-) Reverse	PCE (%) Forward	PCE (%) Reverse
0	24.92	24.92	0.827	0.877	0.451	0.615	9.3	13.5
96	24.95	24.92	0.844	0.864	0.503	0.696	10.6	15.0
408	24.93	24.92	0.875	0.896	0.481	0.617	10.5	13.8
816	24.81	24.85	0.855	0.903	0.516	0.673	10.9	15.1
1296	24.76	24.76	0.836	0.904	0.498	0.653	10.3	14.6
2424	23.99	23.98	0.817	0.900	0.526	0.652	10.3	14.1

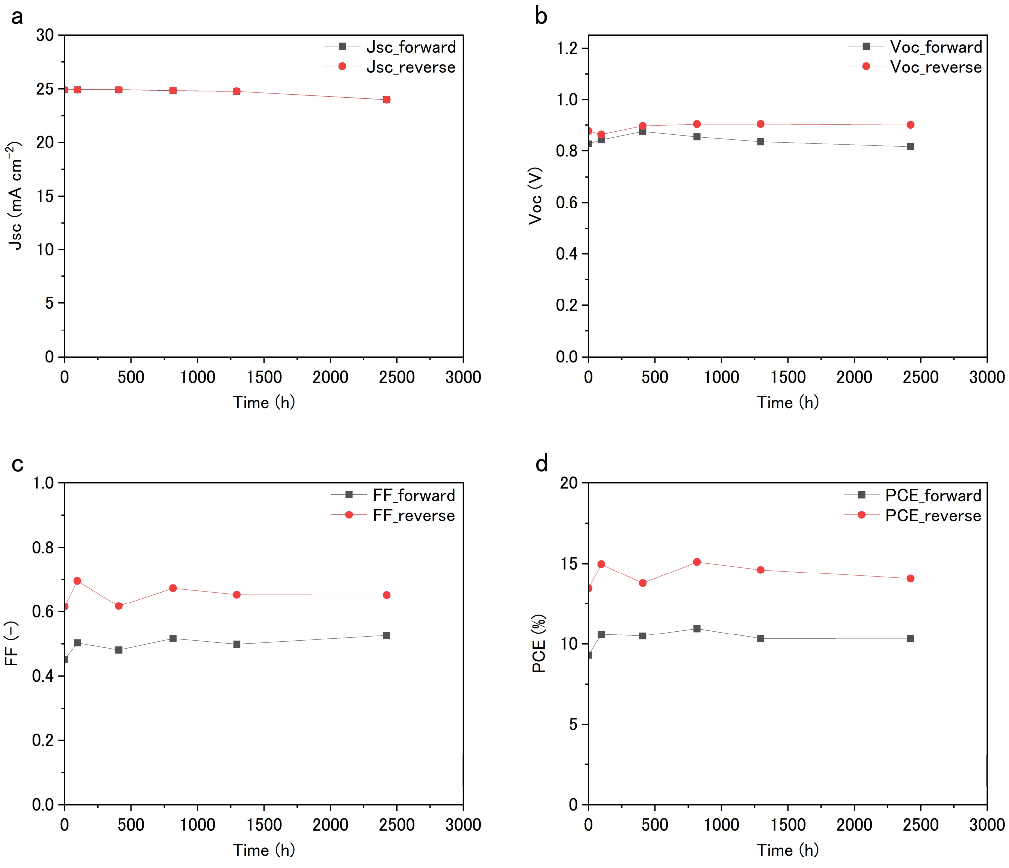

Supplementary Figure 48. Stability result of the perovskite solar cells with Li+K29. (a) J_{SC}, (b) V_{OC}, (c) FF, and (d) PCE as a function of the time with forward and reverse scan. Sample was stored at 85 °C in dark.

Supplementary Table 53. Stability result of the perovskite solar cells with Li+K30. Sample was stored at 85 °C in dark.

Time (h)	Jsc (mA/sq) Forward	Jsc (mA/sq) Reverse	Voc (V) Forward	Voc (V) Reverse	FF (-) Forward	FF (-) Reverse	PCE (%) Forward	PCE (%) Reverse
0	24.86	24.85	0.805	0.864	0.430	0.572	8.6	12.3
96	24.91	24.96	0.844	0.861	0.498	0.686	10.5	14.7
408	24.83	24.87	0.859	0.890	0.501	0.683	10.7	15.1
816	24.76	24.79	0.855	0.899	0.504	0.653	10.7	14.6
1296	24.71	24.76	0.837	0.902	0.491	0.633	10.1	14.1
2424	23.41	23.41	0.827	0.901	0.524	0.645	10.1	13.6

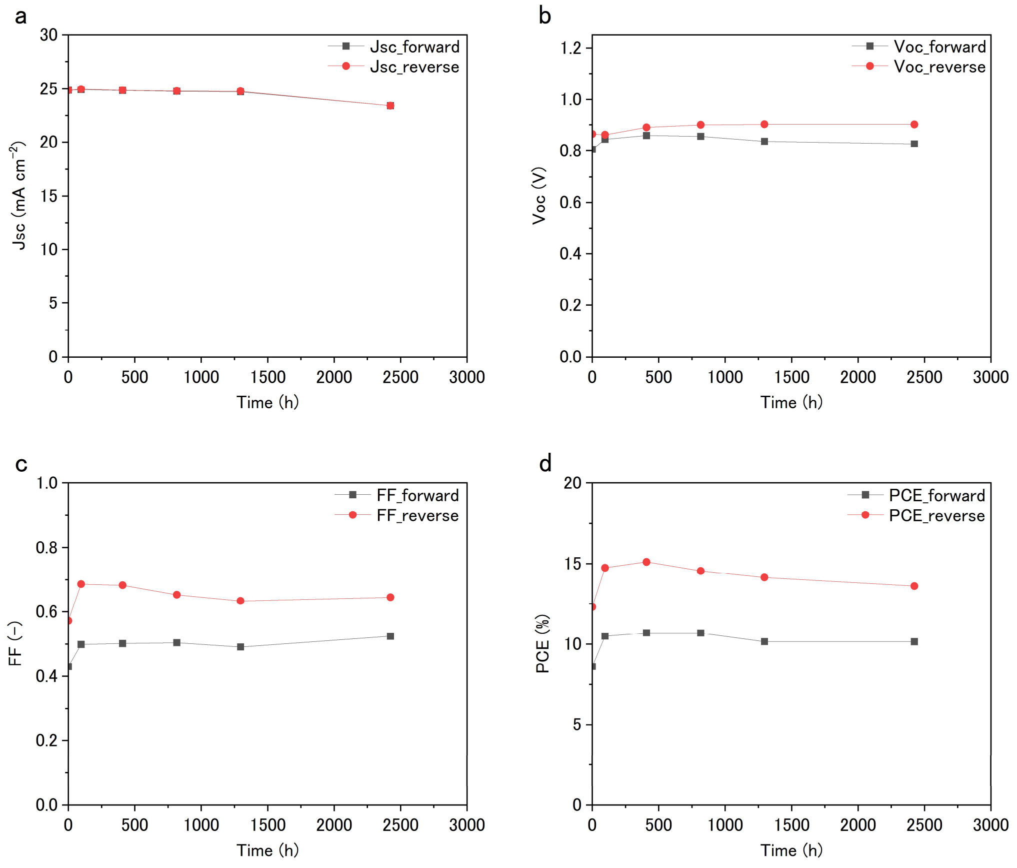

Supplementary Figure 49. Stability result of the perovskite solar cells with Li+K30. (a) J_{SC}, (b) V_{OC}, (c) FF, and (d) PCE as a function of the time with forward and reverse scan. Sample was stored at 85 °C in dark.

Supplementary Table 54. Stability result of the perovskite solar cells with Li+K31. Sample was stored at 85 °C in dark.

Time (h)	Jsc (mA/sq) Forward	Jsc (mA/sq) Reverse	Voc (V) Forward	Voc (V) Reverse	FF (-) Forward	FF (-) Reverse	PCE (%) Forward	PCE (%) Reverse
0	24.40	24.48	0.735	0.801	0.415	0.523	7.4	10.3
96	24.55	24.45	0.875	0.883	0.504	0.621	10.8	13.4
408	24.49	24.46	0.884	0.902	0.494	0.589	10.7	13.0
816	24.33	24.27	0.876	0.906	0.444	0.502	9.5	11.0
1296	24.31	24.19	0.844	0.887	0.441	0.514	9.1	11.0
2424	23.12	22.83	0.828	0.867	0.358	0.412	6.8	8.1

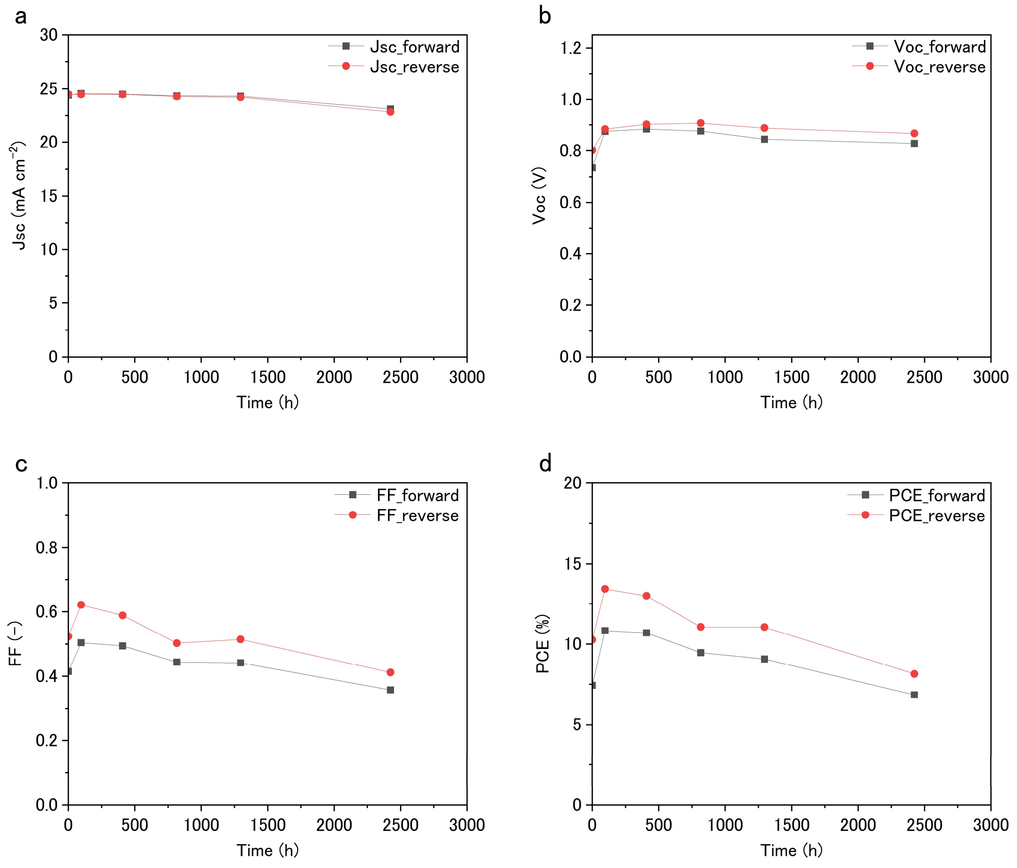

Supplementary Figure 50. Stability result of the perovskite solar cells with Li+K31. (a) J_{SC}, (b) V_{OC}, (c) *FF*, and (d) *PCE* as a function of the time with forward and reverse scan. Sample was stored at 85 °C in dark.

Supplementary Table 55. Stability result of the perovskite solar cells with Li+K32. Sample was stored at 85 °C in dark.

Time (h)	Jsc (mA/sq) Forward	Jsc (mA/sq) Reverse	Voc (V) Forward	Voc (V) Reverse	FF (-) Forward	FF (-) Reverse	PCE (%) Forward	PCE (%) Reverse
0	24.79	24.71	0.897	0.935	0.493	0.611	11.0	14.1
96	24.49	24.31	0.793	0.815	0.435	0.635	8.4	12.6
408	24.55	24.56	0.883	0.903	0.492	0.604	10.7	13.4
1296	23.34	23.35	0.894	0.933	0.473	0.547	9.9	11.9
2424	22.81	22.80	0.894	0.925	0.332	0.386	6.8	8.1

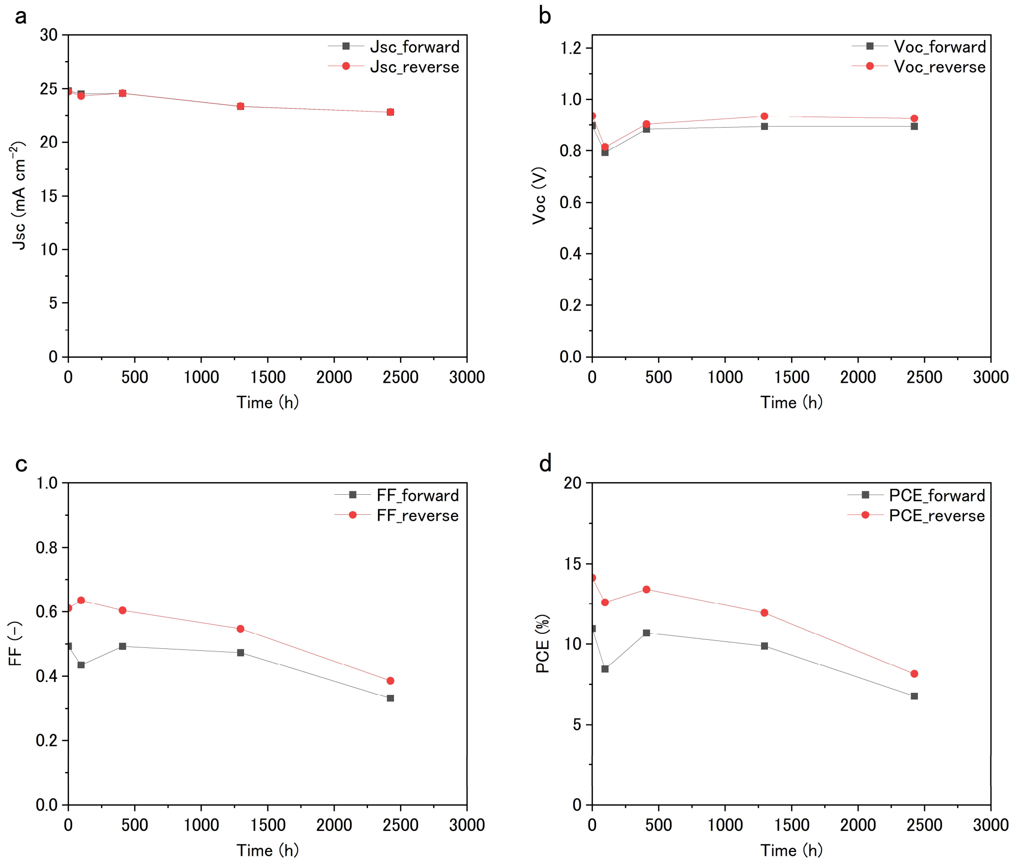

Supplementary Figure 51. Stability result of the perovskite solar cells with Li+K32. (a) J_{SC}, (b) V_{OC}, (c) FF, and (d) PCE as a function of the time with forward and reverse scan. Sample was stored at 85 °C in dark.

Supplementary Table 56. Stability result of the perovskite solar cells with Li+K33. Sample was stored at 85 °C in dark.

Time (h)	Jsc (mA/sq) Forward	Jsc (mA/sq) Reverse	Voc (V) Forward	Voc (V) Reverse	FF (-) Forward	FF (-) Reverse	PCE (%) Forward	PCE (%) Reverse
0	24.87	24.76	0.922	1.001	0.383	0.546	8.8	13.5
72	7.79	6.49	0.946	0.945	0.203	0.262	1.5	1.6

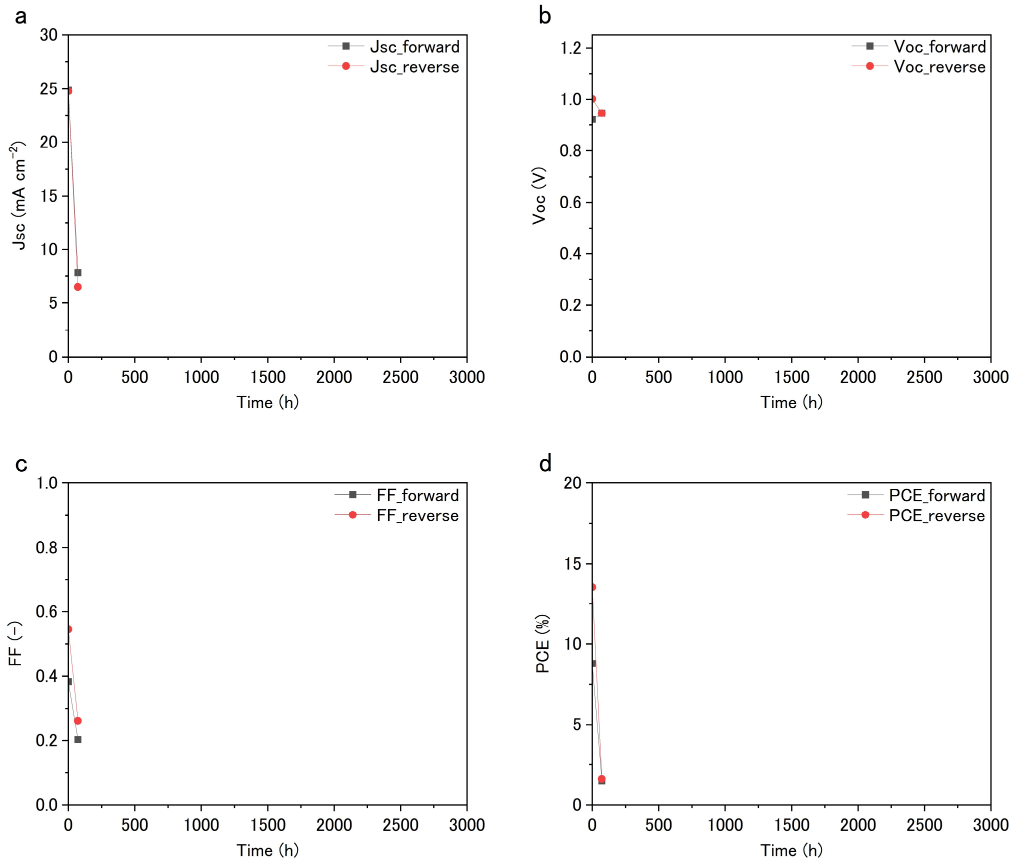

Supplementary Figure 52. Stability result of the perovskite solar cells with Li+K33. (a) J_{SC}, (b) V_{OC}, (c) *FF*, and (d) *PCE* as a function of the time with forward and reverse scan. Sample was stored at 85 °C in dark.

Supplementary Table 57. Stability result of the perovskite solar cells with Li,Co+K33. Sample was stored at 85 °C in dark.

Time (h)	Jsc (mA/sq) Forward	Jsc (mA/sq) Reverse	Voc (V) Forward	Voc (V) Reverse	FF (-) Forward	FF (-) Reverse	PCE (%) Forward	PCE (%) Reverse
0	13.36	8.86	0.765	0.886	0.115	0.278	1.2	2.2
72	2.39	2.05	0.704	0.623	0.323	0.269	0.5	0.3

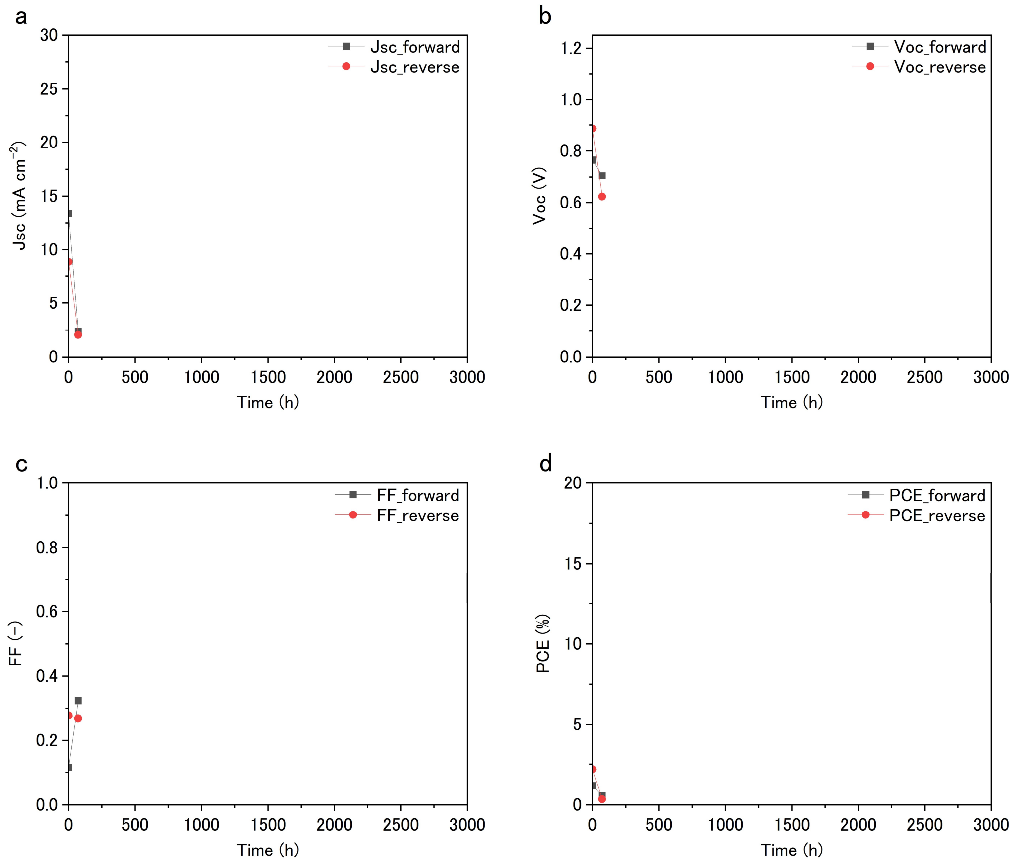

Supplementary Figure 53. Stability result of the perovskite solar cells with Li,Co+K33. (a) J_{SC}, (b) V_{OC}, (c) *FF*, and (d) *PCE* as a function of the time with forward and reverse scan. Sample was stored at 85 °C in dark.

Supplementary Table 58. Stability result of the perovskite solar cells with Li+K34. Sample was stored at 85 °C in dark.

Time (h)	Jsc (mA/sq) Forward	Jsc (mA/sq) Reverse	Voc (V) Forward	Voc (V) Reverse	FF (-) Forward	FF (-) Reverse	PCE (%) Forward	PCE (%) Reverse
0	24.85	24.87	0.999	1.032	0.524	0.646	13.0	16.6
96	24.62	24.48	0.829	0.873	0.517	0.613	10.5	13.1
408	24.51	24.24	0.826	0.841	0.460	0.531	9.3	10.8
816	22.45	22.42	0.752	0.745	0.406	0.401	6.9	6.7
1296	17.78	20.09	0.707	0.689	0.396	0.288	5.0	4.0
2424	4.53	6.27	0.661	0.658	0.319	0.176	1.0	0.7

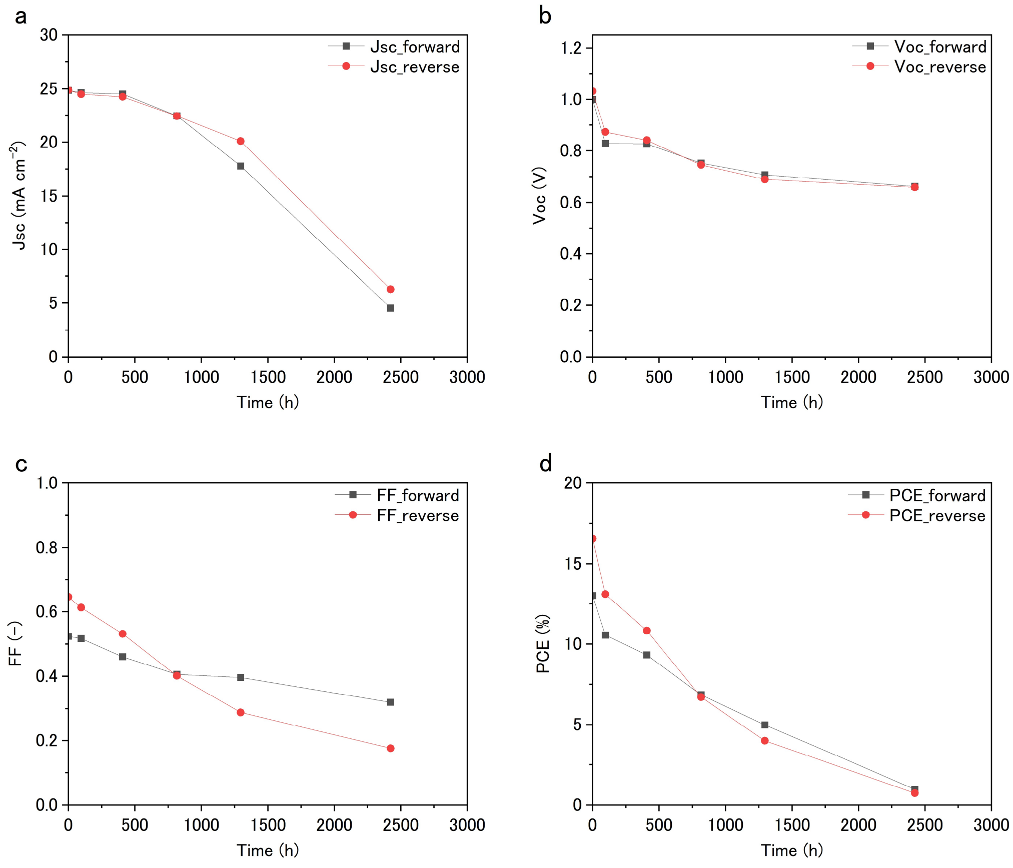

Supplementary Figure 54. Stability result of the perovskite solar cells with Li+K34. (a) J_{SC}, (b) V_{OC}, (c) FF, and (d) PCE as a function of the time with forward and reverse scan. Sample was stored at 85 °C in dark.

Supplementary Table 59. Stability result of the perovskite solar cells with Li+K35. Sample was stored at 85 °C in dark.

Time (h)	Jsc (mA/sq) Forward	Jsc (mA/sq) Reverse	Voc (V) Forward	Voc (V) Reverse	FF (-) Forward	FF (-) Reverse	PCE (%) Forward	PCE (%) Reverse
0	24.70	24.73	0.854	0.923	0.497	0.683	10.5	15.6
72	24.71	24.40	0.854	0.853	0.287	0.381	6.1	7.9

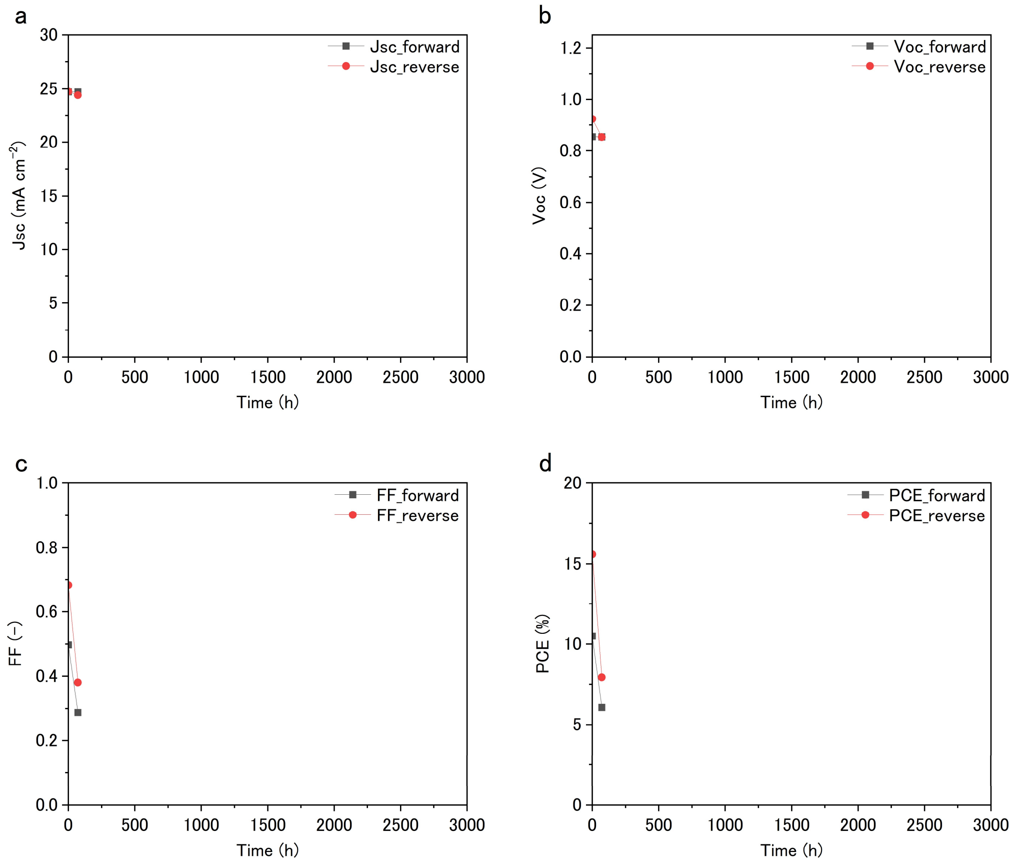

Supplementary Figure 55. Stability result of the perovskite solar cells with Li+K35. (a) J_{SC}, (b) V_{OC}, (c) FF, and (d) PCE as a function of the time with forward and reverse scan. Sample was stored at 85 °C in dark.

Supplementary Table 60. Stability result of the perovskite solar cells with Li,Co+K35. Sample was stored at 85 °C in dark.

Time (h)	Jsc (mA/sq) Forward	Jsc (mA/sq) Reverse	Voc (V) Forward	Voc (V) Reverse	FF (-) Forward	FF (-) Reverse	PCE (%) Forward	PCE (%) Reverse
0	25.23	25.12	0.875	0.928	0.381	0.541	8.4	12.6
72	13.36	11.12	0.819	0.825	0.224	0.292	2.5	2.7

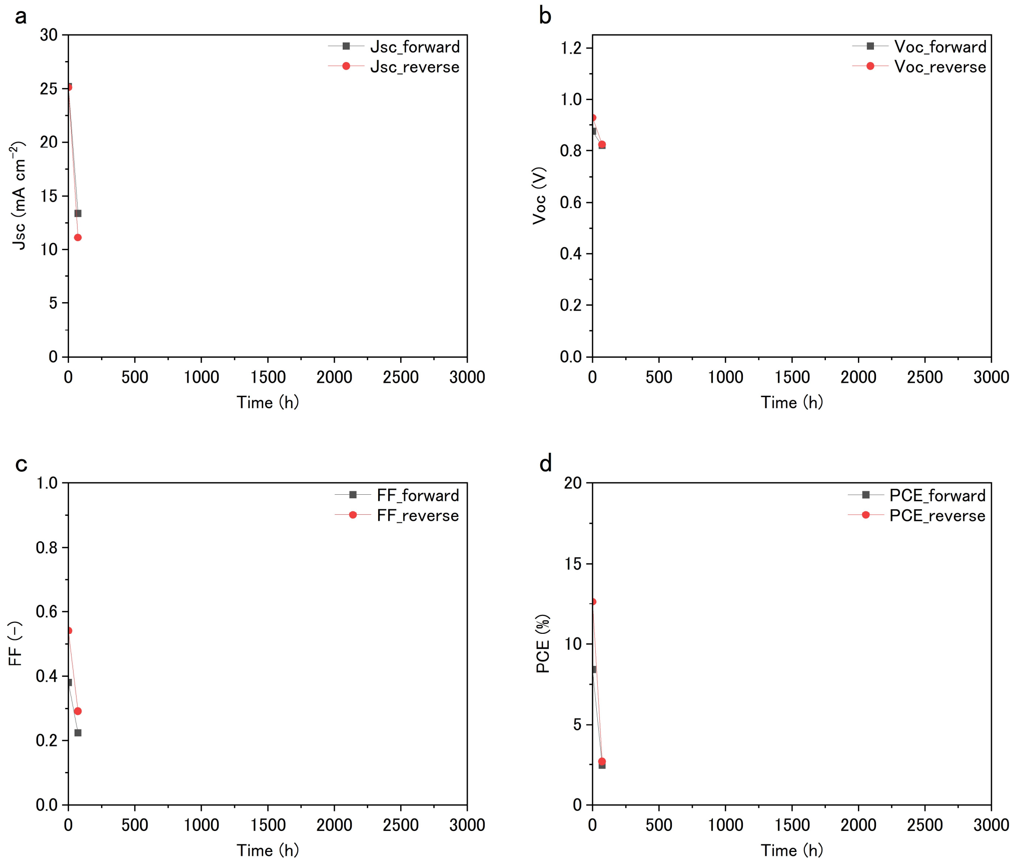

Supplementary Figure 56. Stability result of the perovskite solar cells with Li,Co+K35. (a) J_{SC}, (b) V_{OC}, (c) *FF*, and (d) *PCE* as a function of the time with forward and reverse scan. Sample was stored at 85 °C in dark.

Supplementary Table 61. Stability result of the perovskite solar cells with Li+K36. Sample was stored at 85 °C in dark.

Time (h)	Jsc (mA/sq) Forward	Jsc (mA/sq) Reverse	Voc (V) Forward	Voc (V) Reverse	FF (-) Forward	FF (-) Reverse	PCE (%) Forward	PCE (%) Reverse
0	24.69	24.63	0.813	0.891	0.427	0.609	8.6	13.4
72	2.16	2.09	0.860	0.860	0.194	0.209	0.4	0.4

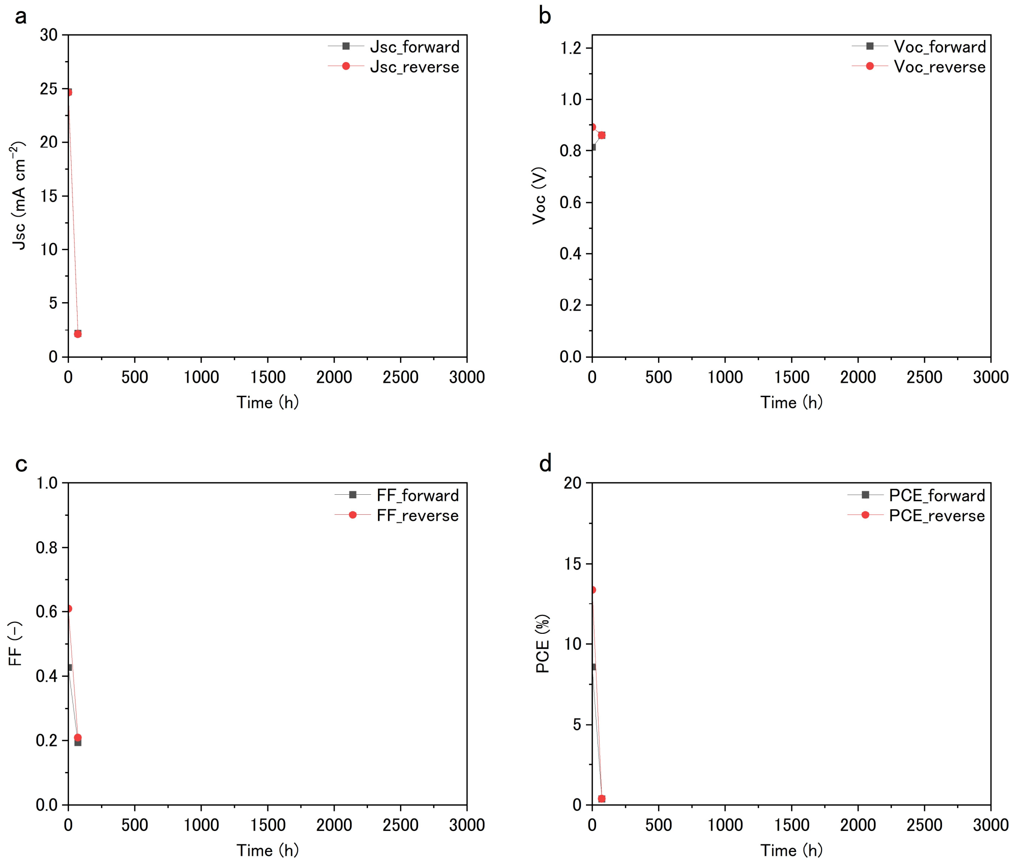

Supplementary Figure 57. Stability result of the perovskite solar cells with Li+K36. (a) J_{SC}, (b) V_{OC}, (c) *FF*, and (d) *PCE* as a function of the time with forward and reverse scan. Sample was stored at 85 °C in dark.

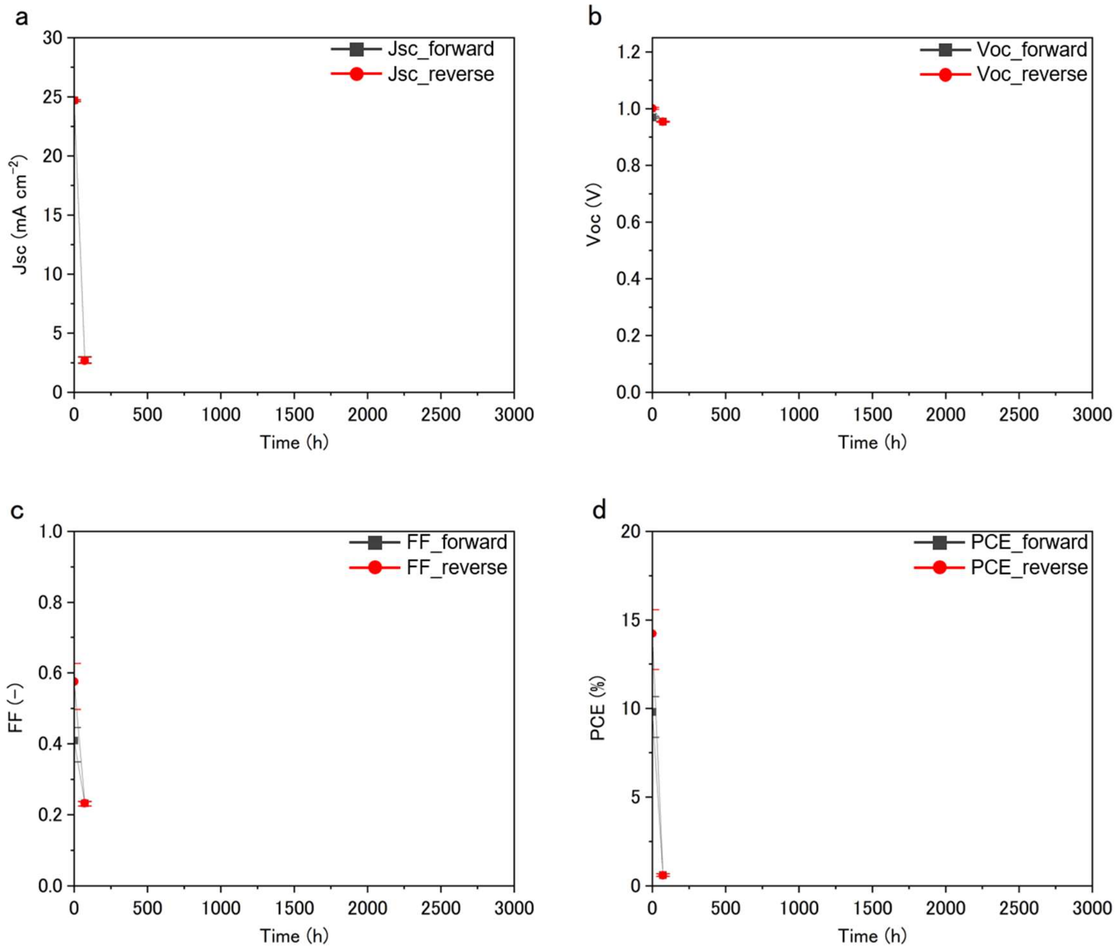

Supplementary Figure 58. Statistics stability result of the perovskite solar cells with Li+tBP. (a) J_{SC}, (b) V_{OC}, (c) *FF*, and (d) *PCE* as a function of the time with forward and reverse scan. Sample was stored at 85 ℃ in dark. Number of samples was 3. Plots shows maximum, minimum, and average of each photovoltaic parameter.

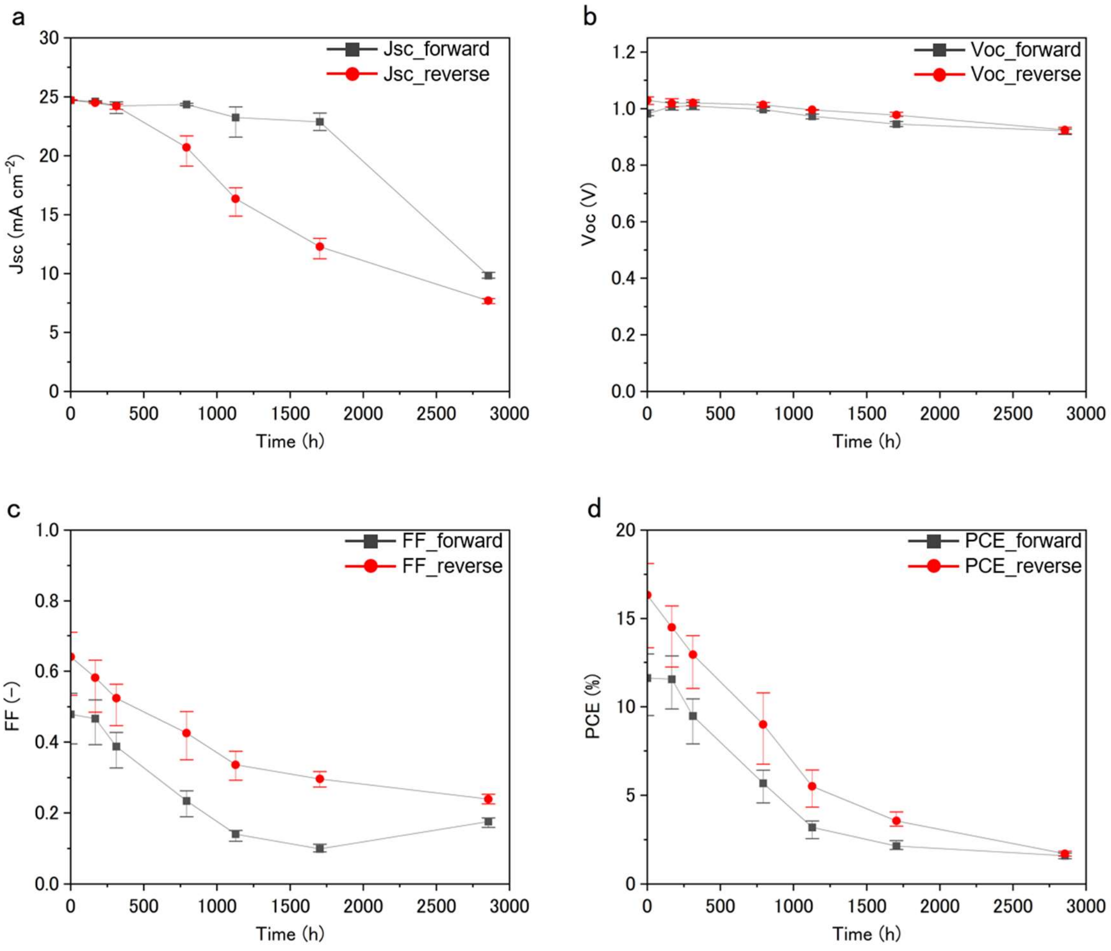

Supplementary Figure 59. Statistics stability result of the perovskite solar cells with Li,Co+tBP. (a) J_{SC}, (b) V_{OC}, (c) FF, and (d) PCE as a function of the time with forward and reverse scan. Sample was stored at 85 ℃ in dark. Number of samples was 3. Plots shows maximum, minimum, and average of each photovoltaic parameter.

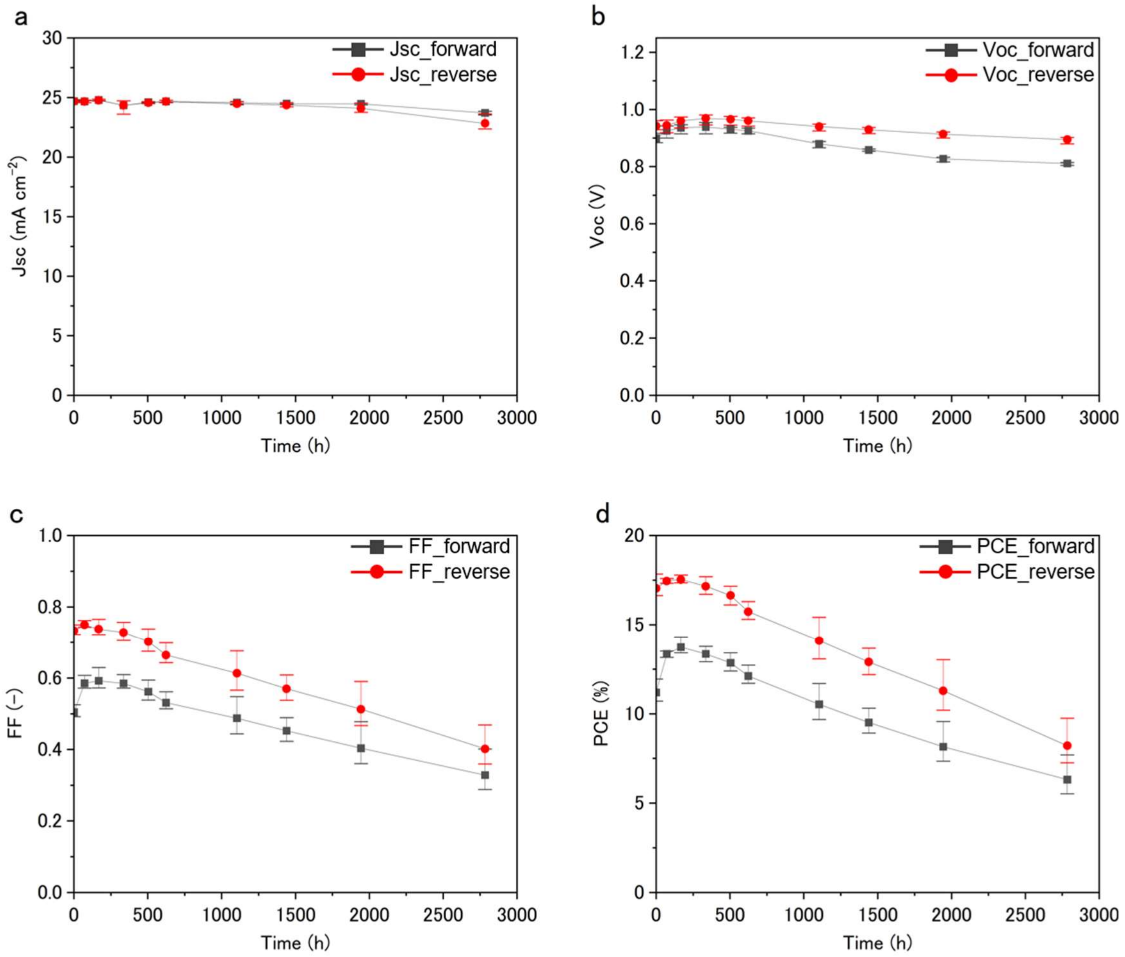

Supplementary Figure 60. Statistics stability result of the perovskite solar cells with Li+K27. (a) J_{SC}, (b) V_{OC}, (c) *FF*, and (d) *PCE* as a function of the time with forward and reverse scan. Sample was stored at 85 °C in dark. Number of samples was 3. Plots shows maximum, minimum, and average of each photovoltaic parameter.

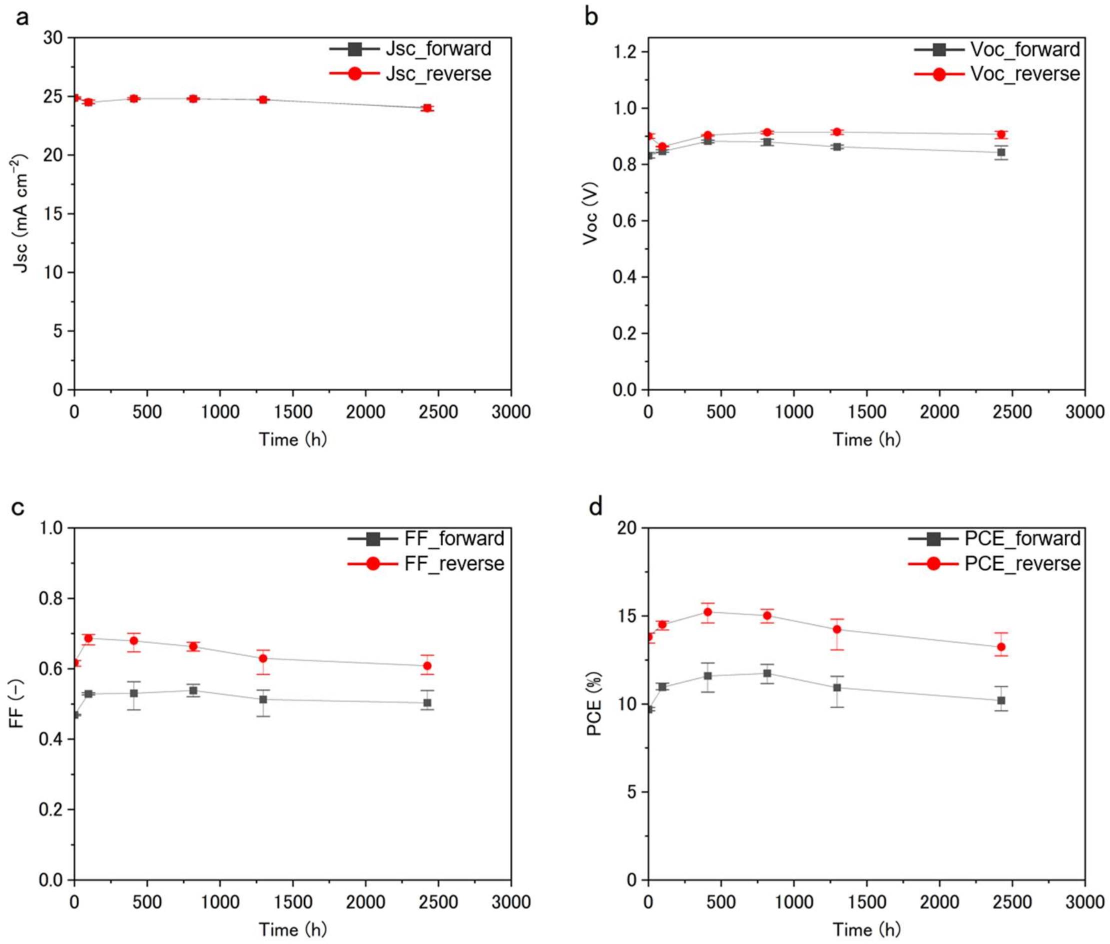

Supplementary Figure 61. Statistics stability result of the perovskite solar cells with Li+K28. (a) J_{SC}, (b) V_{OC}, (c) *FF*, and (d) *PCE* as a function of the time with forward and reverse scan. Sample was stored at 85 °C in dark. Number of samples was 3. Plots shows maximum, minimum, and average of each photovoltaic parameter.

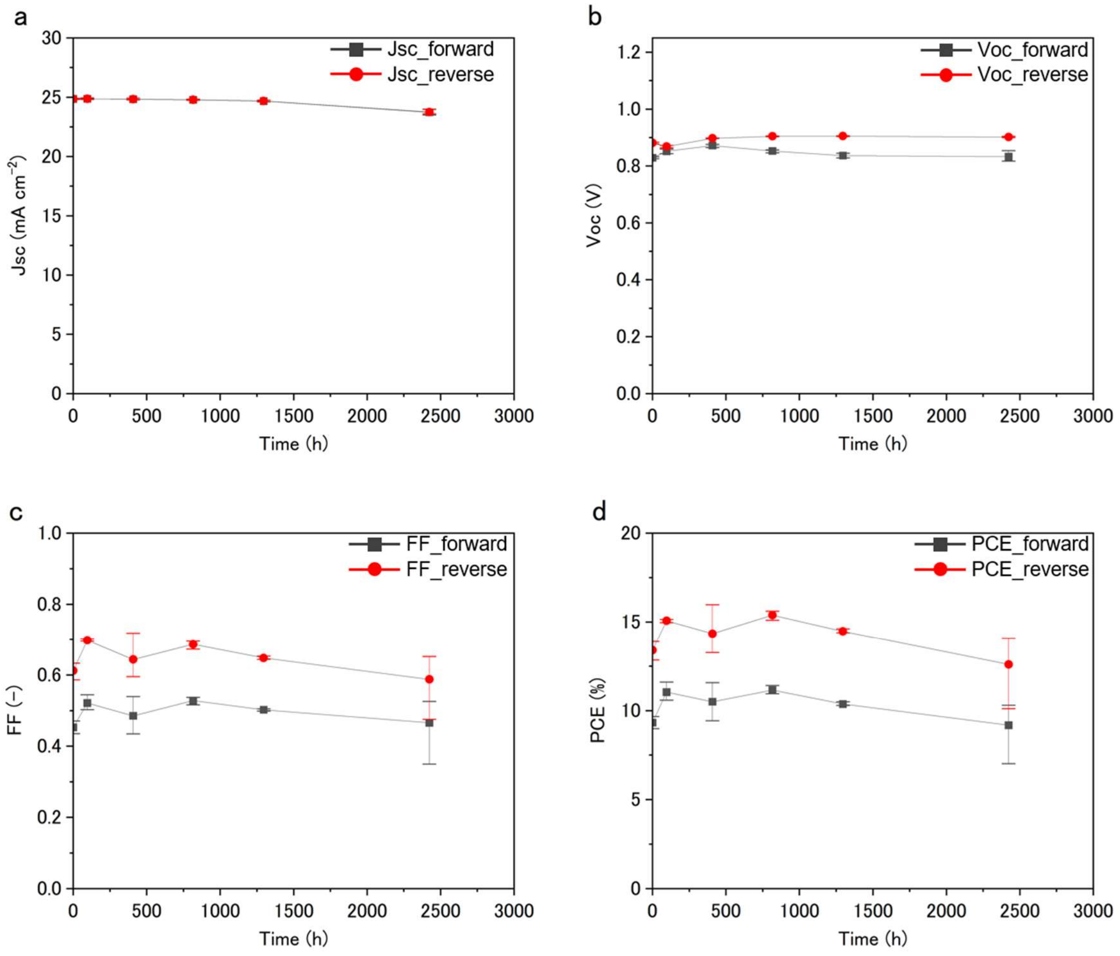

Supplementary Figure 62. Statistics stability result of the perovskite solar cells with Li+K29. (a) J_{SC}, (b) V_{OC}, (c) FF, and (d) PCE as a function of the time with forward and reverse scan. Sample was stored at 85 °C in dark. Number of samples was 3. Plots shows maximum, minimum, and average of each photovoltaic parameter.

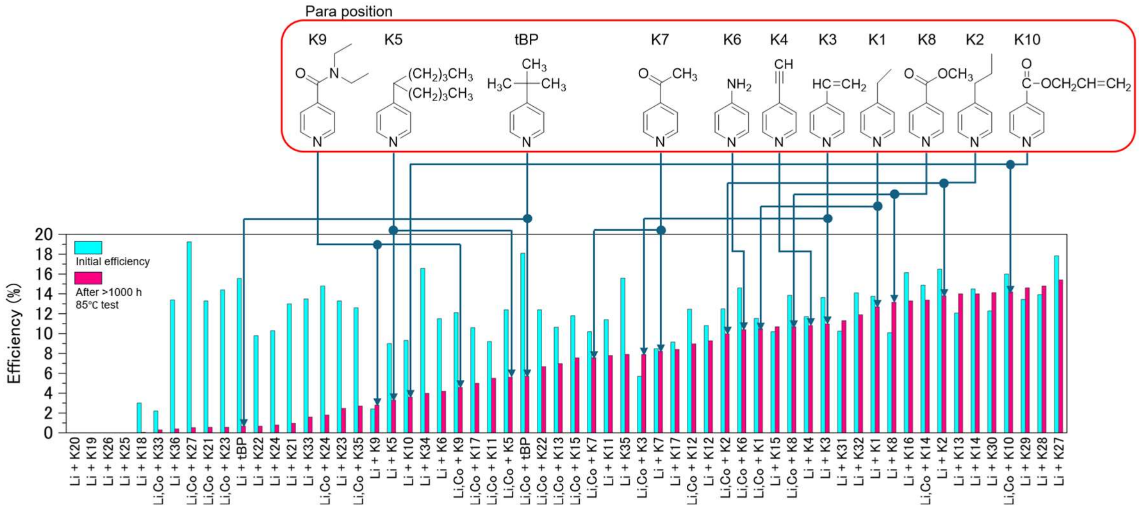

Supplementary Figure 63. Summarized 85 ℃ stability results of perovskite solar cells with pyridine derivatives substituted with para position. This substitution does not include phenyl group.

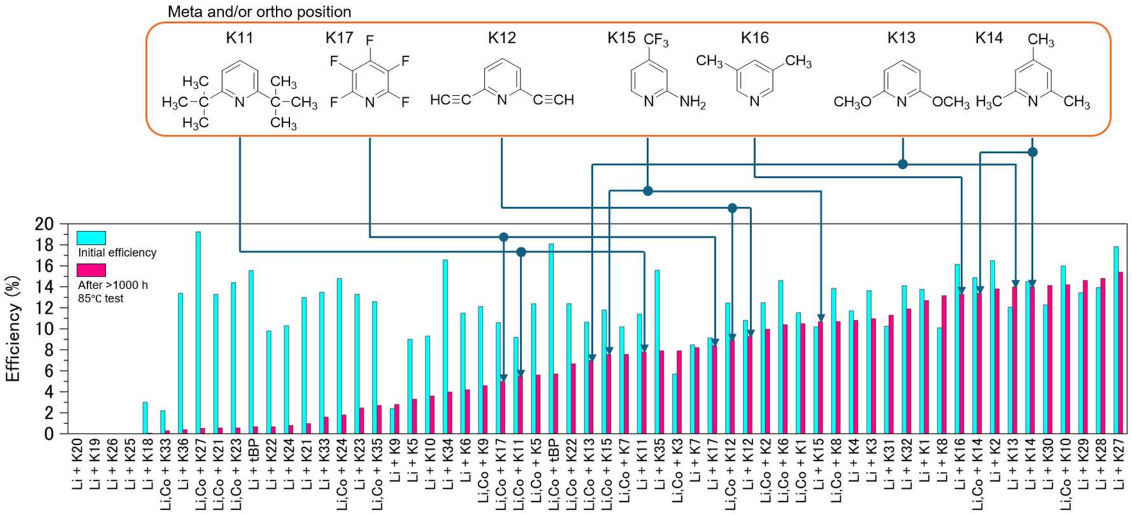

Supplementary Figure 64. Summarized 85 ℃ stability results of perovskite solar cells with pyridine derivatives substituted with meta and/or ortho position. This substitution does not include

phenyl group.

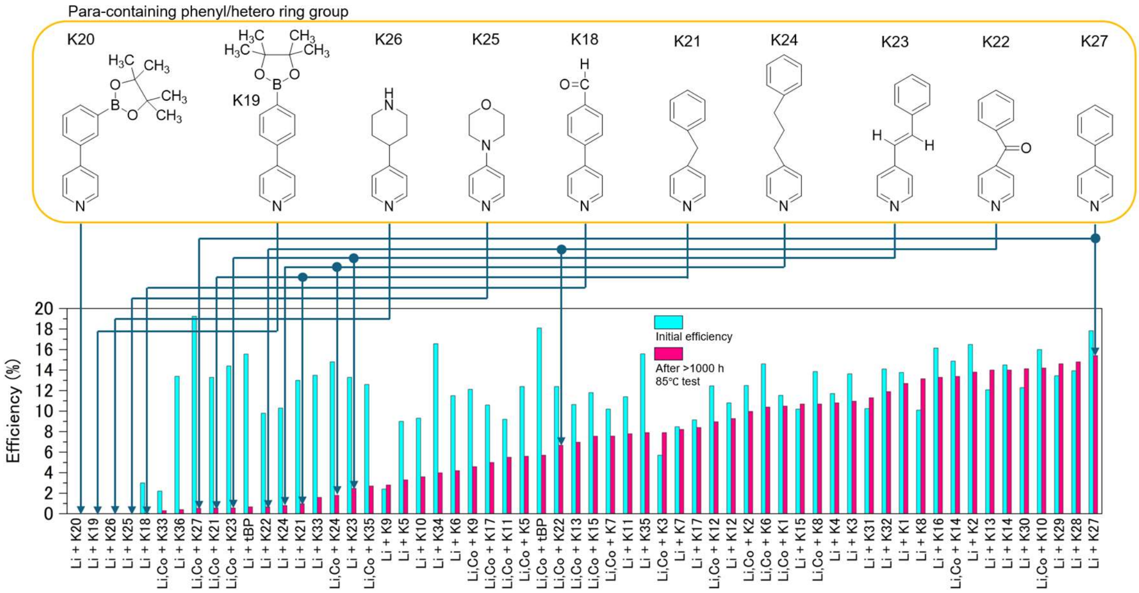

Supplementary Figure 65. Summarized 85 °C stability results of perovskite solar cells with pyridine derivatives substituted with para-containing phenyl/hetero ring group.

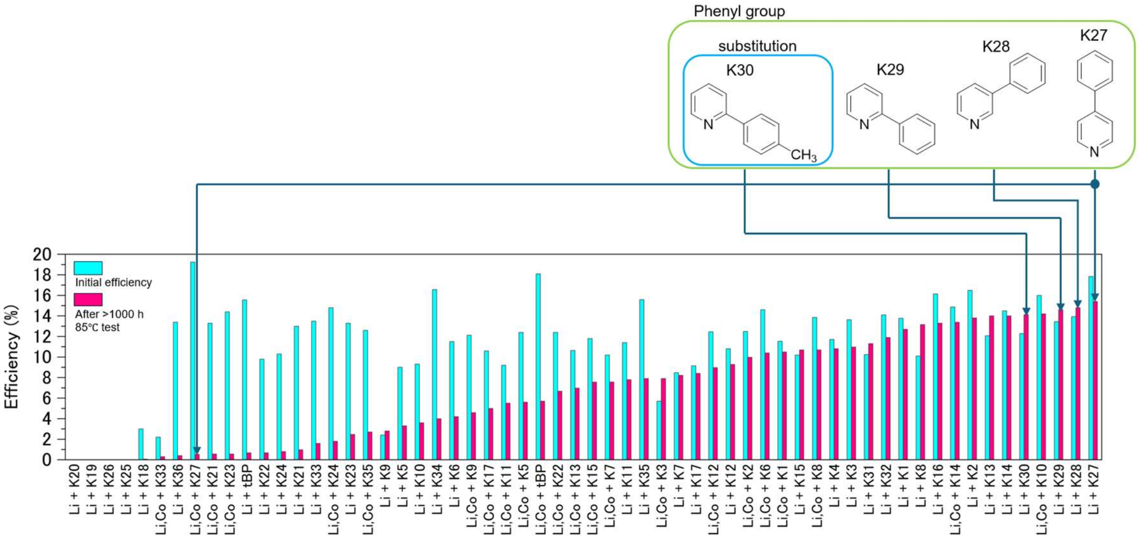

Supplementary Figure 66. Summarized 85 °C stability results of perovskite solar cells using

pyridine derivatives with phenyl group of para, meta, ortho, and substitution.

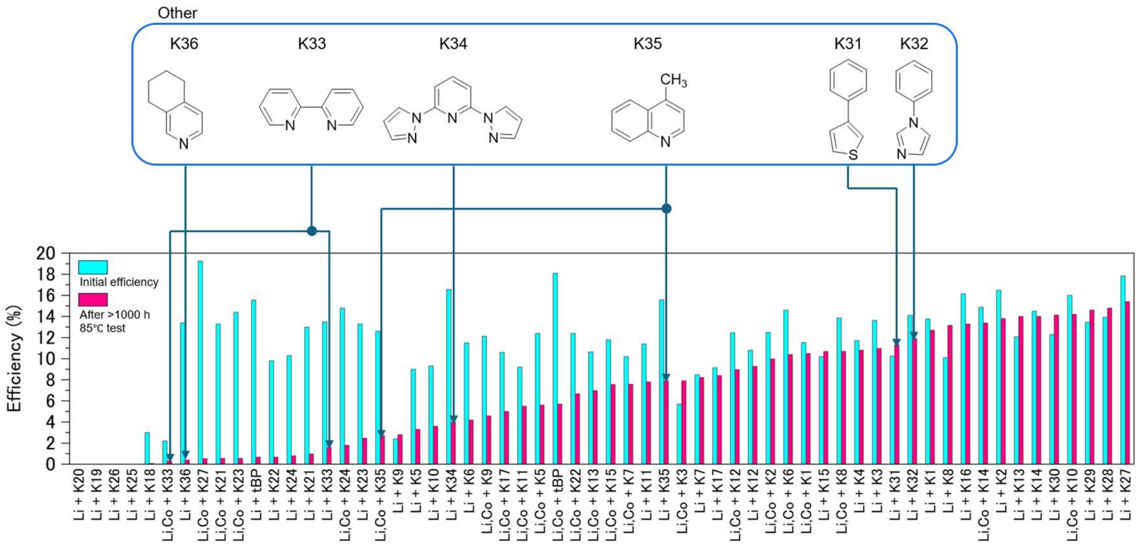

Supplementary Figure 67. Summarized 85 ℃ stability results of perovskite solar cells with other additives including thiophene, imidazole, and unique structures such as bipyridyl.

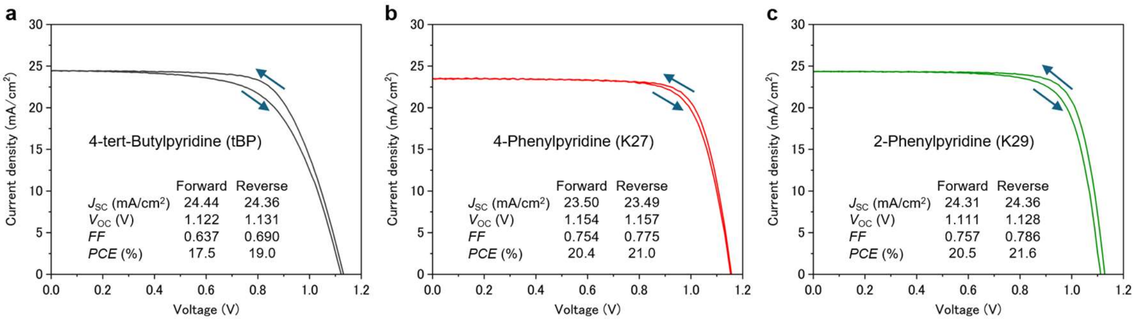

Supplementary Figure 68. Photovoltaic performances of the perovskite solar cells with each additive. *I-V* scans of the perovskite solar cells with (a) 4-*tert*-butylpyridine (Li+tBP), (b) 4-phenylpyridine (Li+K27), and (c) 2-phenylpyridine (Li+K29). OAI passivation was introduced to these devices.

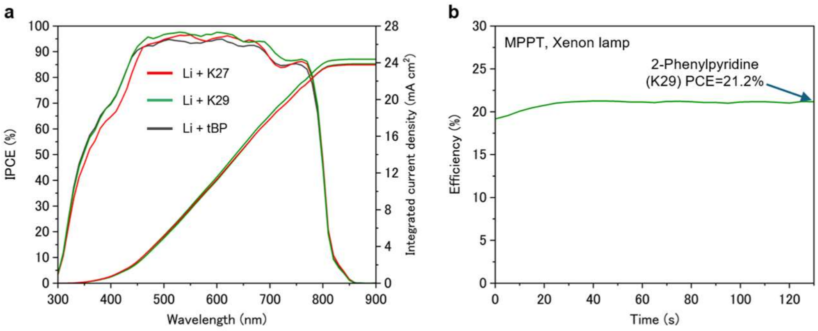

Supplementary Figure 69. (a) IPCE of perovskite solar cells with 4-*tert*-butylpyridine (tBP), 4-phenylpyridine (K27), and 2-phenylpyridine (K29). (b) MPP tracking of device with 2-phenylpyridine (K29). OAI passivation was introduced to these devices.

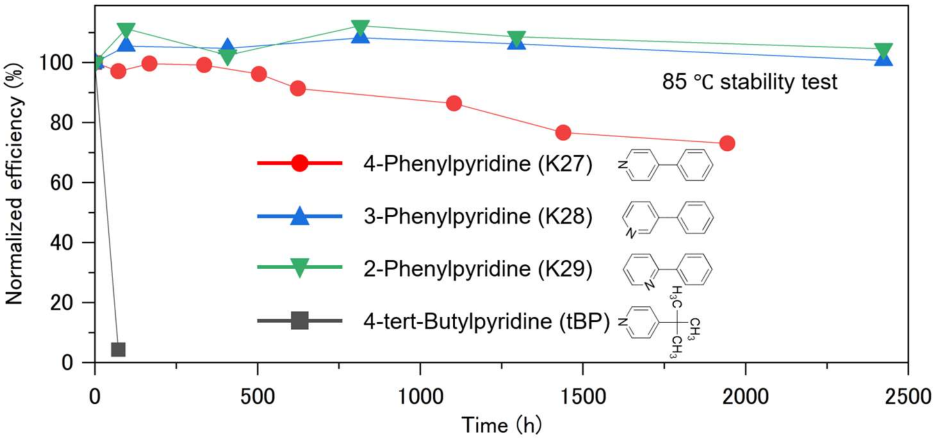

Supplementary Figure 70. Normalized 85 ℃ thermal stability result with 4-phenylpyridine (Li+K27), 3-phenylpyridine (Li+K28), 2-phenylpyridine (Li+K29), and 4-tert-butylpyridine (Li+tBP).

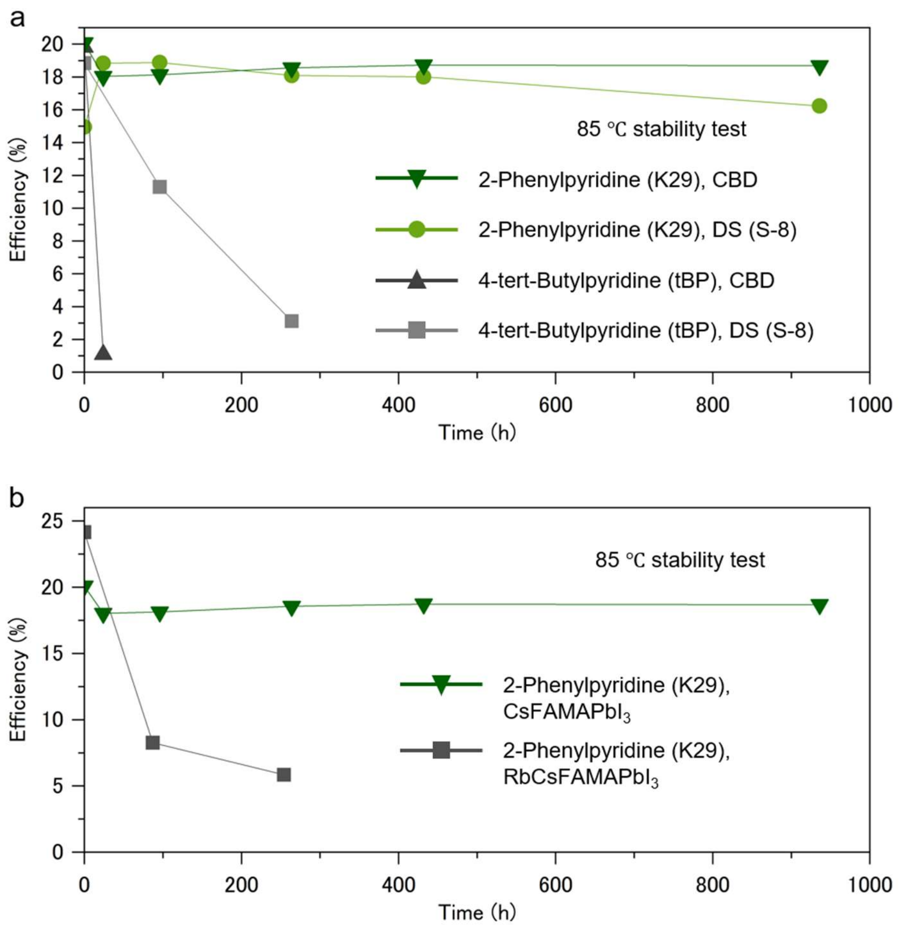

Supplementary Figure 71. Stability comparison of perovskite solar cells. (a) dispersed-solution-SnO_2 (SnO_2_DS) vs chemical bath deposition (SnO_2_CBD). (b) $CsFAPbI_3$ vs $RbCsFAPbI_3$. MACl concentration increased to 20% from 10% to achieve higher efficiency.

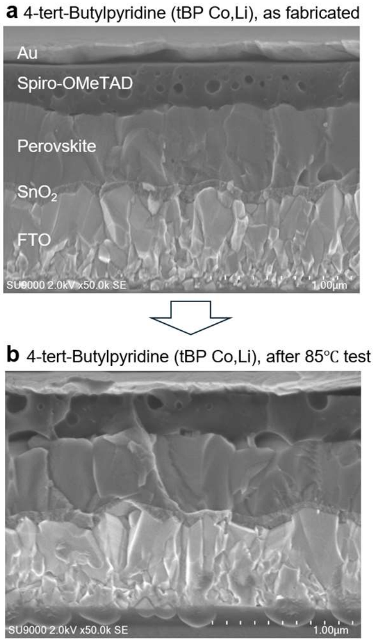

Supplementary Figure 72. SEM images of perovskite solar cells with 4-*tert*-butylpyridine (tBP) (a) before and (b) after 400 hours of 85 ℃ tests. Spiro-OMeTAD layer contains 4-*tert*-butylpyridine (tBP), Co(III) TFSI (FK 209), and LiTFSI (Co,Li+tBP).

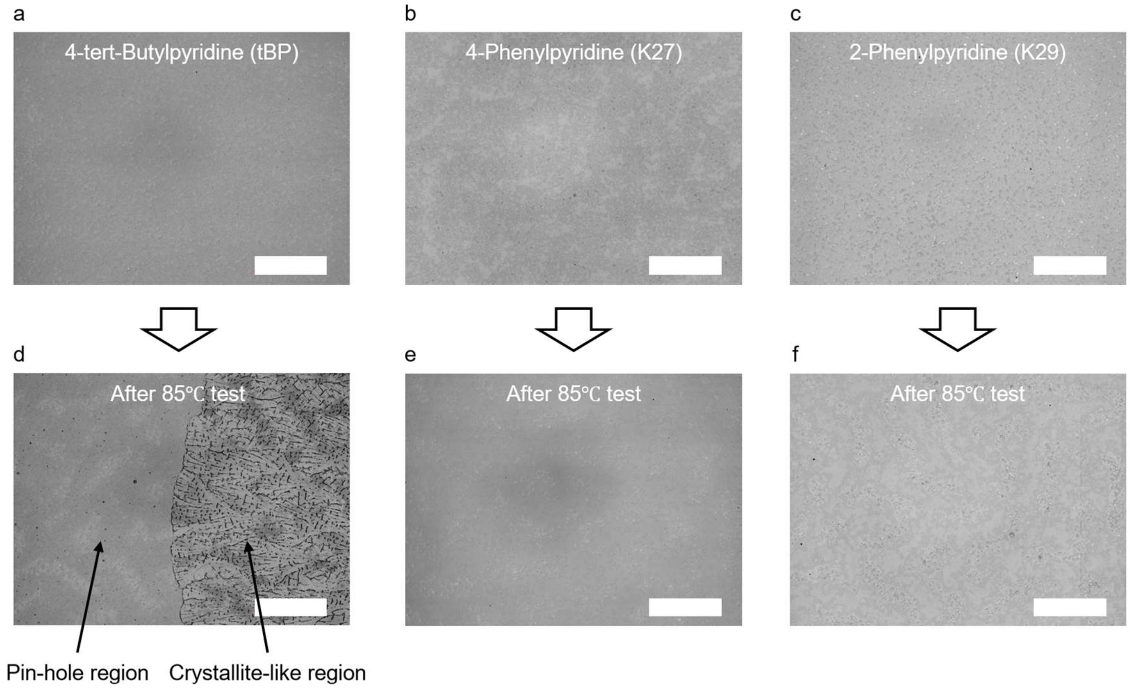

Supplementary Figure 73. Optical microscope images of the HTLs before and after thermal test. Additives with (a) 4-tert-butylpyridine, (b) 4-phenylpyridine, (c) 2-phenylpyridine and (d-f) corresponding additives after 85 °C test for 100 hours. Sample structure was FTO glass/SnO_2/Perovskite/Sprio-OMeTAD/Au. Spiro-OMeTAD layer contains LiTFSI and each additives same as device fabrication. Au layer was pealed out by tape just before measurement. Scale bar corresponds to 30 μm.

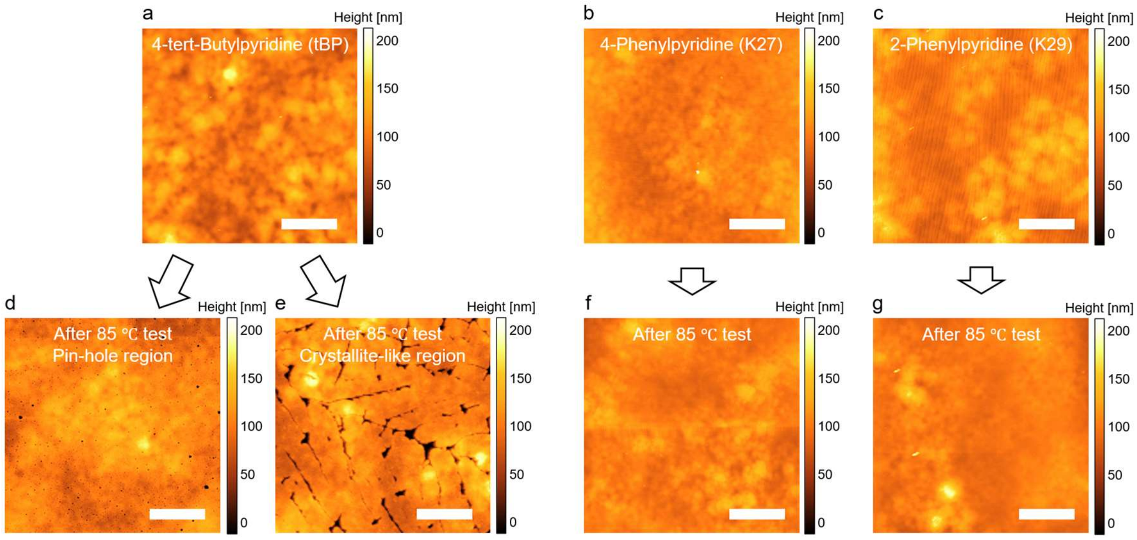

Supplementary Figure 74. AFM images of the HTLs before and after thermal aging. Additives with (a) 4-tert-butylpyridine, (b) 4-phenylpyridine, (c) 2-phenylpyridine, and (d-g) corresponding additives after 85 °C test for 100 hours. Sample structure was FTO glass/SnO_2/Perovskite/Sprio-OMeTAD/Au. Spiro-OMeTAD layer contains LiTFSI and each additives same as device fabrication. Au layer was pealed out by tape just before measurement. Scale bar corresponds to 5 μm.

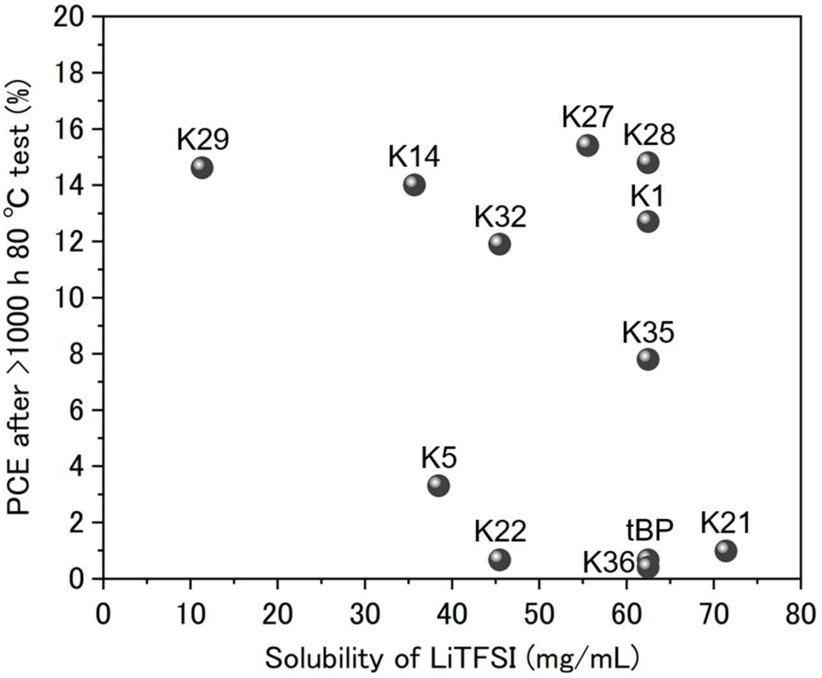

Supplementary Figure 75. PCE after 1000 hours *vs* solubility of LiTFSI with each additive. Lithium salts were dissolved in Spiro-OMeTAD solutions containing each additive. Concentration of the Spiro-OMeTAD and each additive were same as device fabrication.

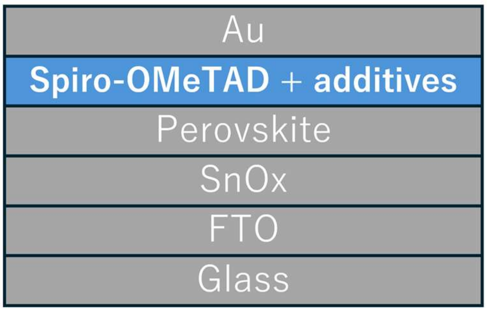

Supplementary Figure 76. Sample structure for XRD measurement. Each layer was deposited using the same recipe for device fabrication. Au layer was pealed out by using tape just before XRD measurement.

Supplementary Table 62. Fitting parameters for time-resolved PL spectra.

Structure	A1	τ_a(ns)	A2	τ_b (ns)
Perovskite only	0.246	7.96	0.772	5806.1
Perovskite/Spiro-OMeTAD with 4-*tert*-butylpyridine (tBP)	0.509	1.44	0.548	43.7
Perovskite/Spiro-OMeTAD with 4-phenylpyridine (K27)	1.634	1.13	0.429	18.5
Perovskite/Spiro-OMeTAD with 2-phenylpyridine (K29)	0.561	1.71	0.535	13.2

†The equation for the fitting is $y=y_0+A_1\times\exp(-(x-x_0)/\tau_a)+A_2\times\exp(-(x-x_0)/\tau_b)$.

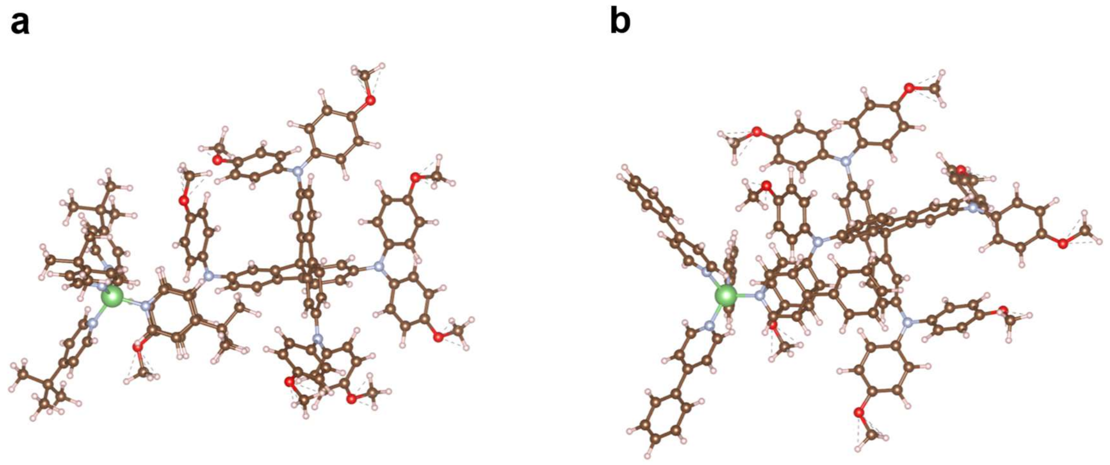

Supplementary Figure 77 DFT calculated chemical structure a, 4-*tert*-Butylpyridine (tBP), lithium and Spiro-OMeTAD. b, 4-Phenylpyridine (K27), lithium and Spiro-OMeTAD.

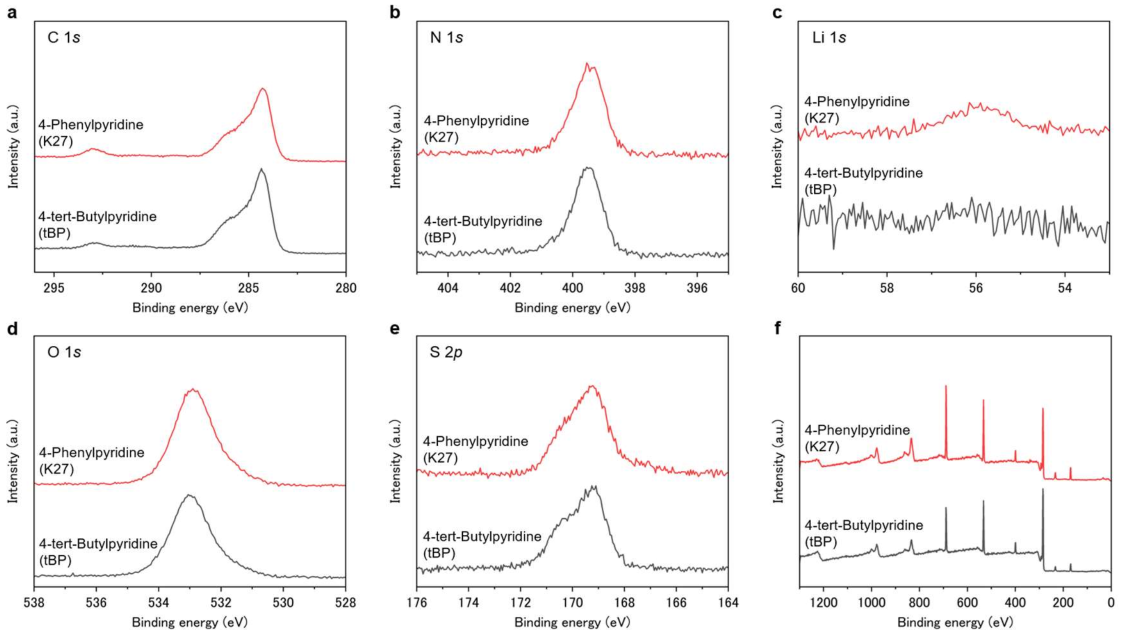

Supplementary Figure 78. XPS spectra of Spiro-OMeTAD with LiTFSI+4-*tert*-butylpyridine (Li+tBP) and LiTFSI+4-phenylpyridine (Li+K27). (a) C 1*s*, (b) N 1*s*, (c) Li 1*s*, (d) O 1*s*, (e) S 2*p*, and (f) survey scan.

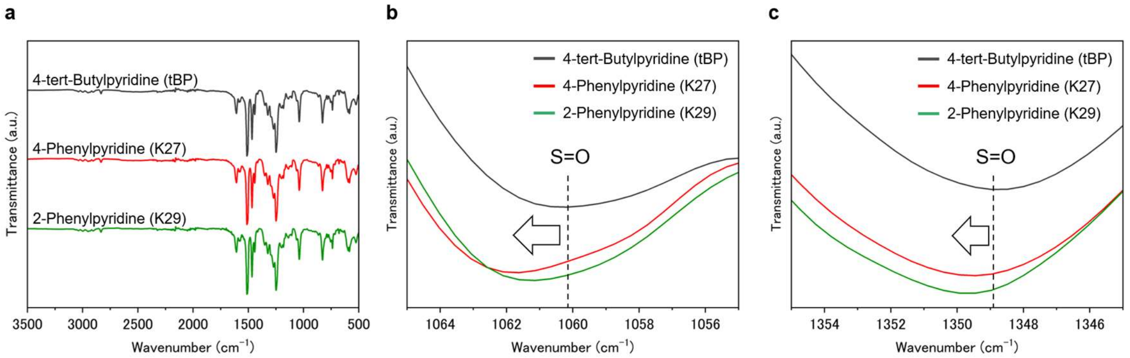

Supplementary Figure 79. FTIR spectra of Spiro-OMeTAD with LiTFSI and different dopants: (a) full spectrum and expanded views of (b) 1055–1066 cm^{-1} and (c) 1345–1355 cm^{-1}.

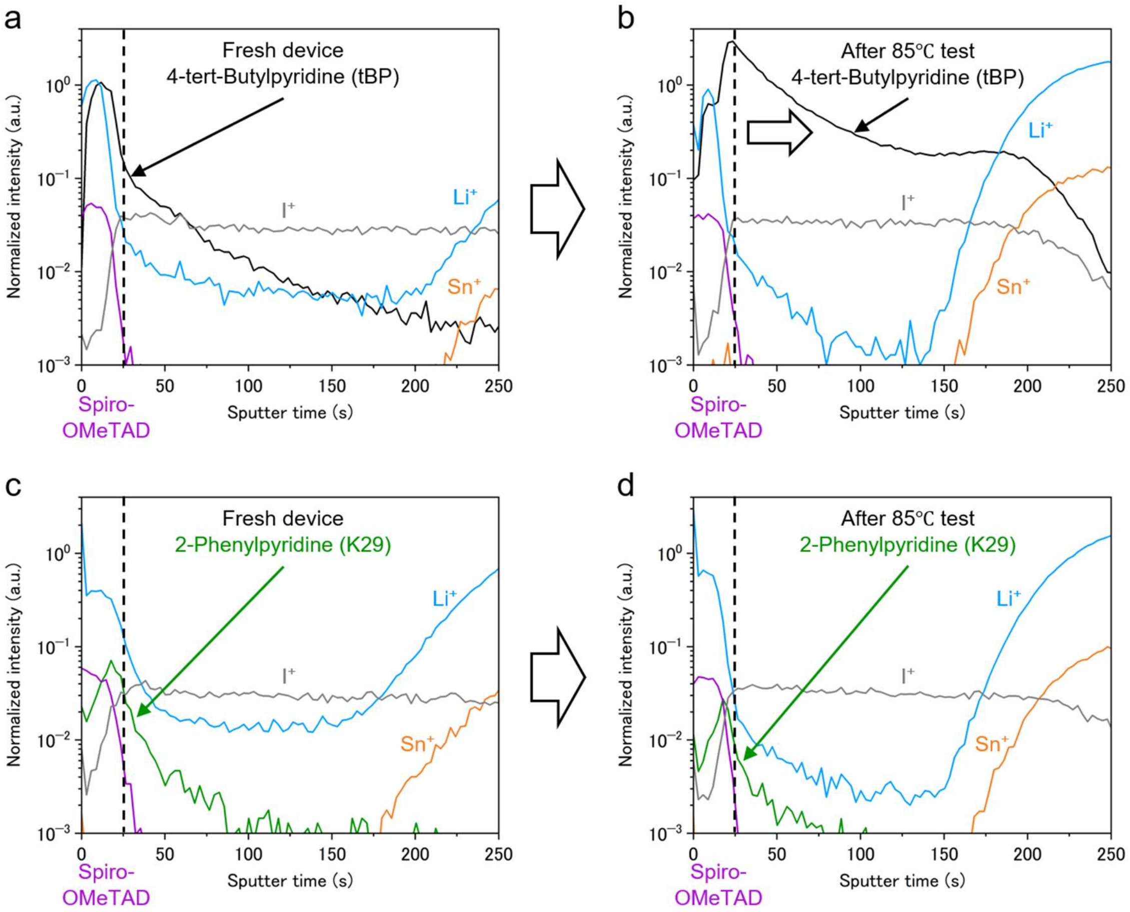

Supplementary Figure 80. ToF-SIMS depth profiles of (a) a fresh device with 4-tert-butylpyridine, (b) the same device after the 85 °C test, (c) a fresh device with 2-phenylpyridine, and (d) the same device after the 85 °C test.

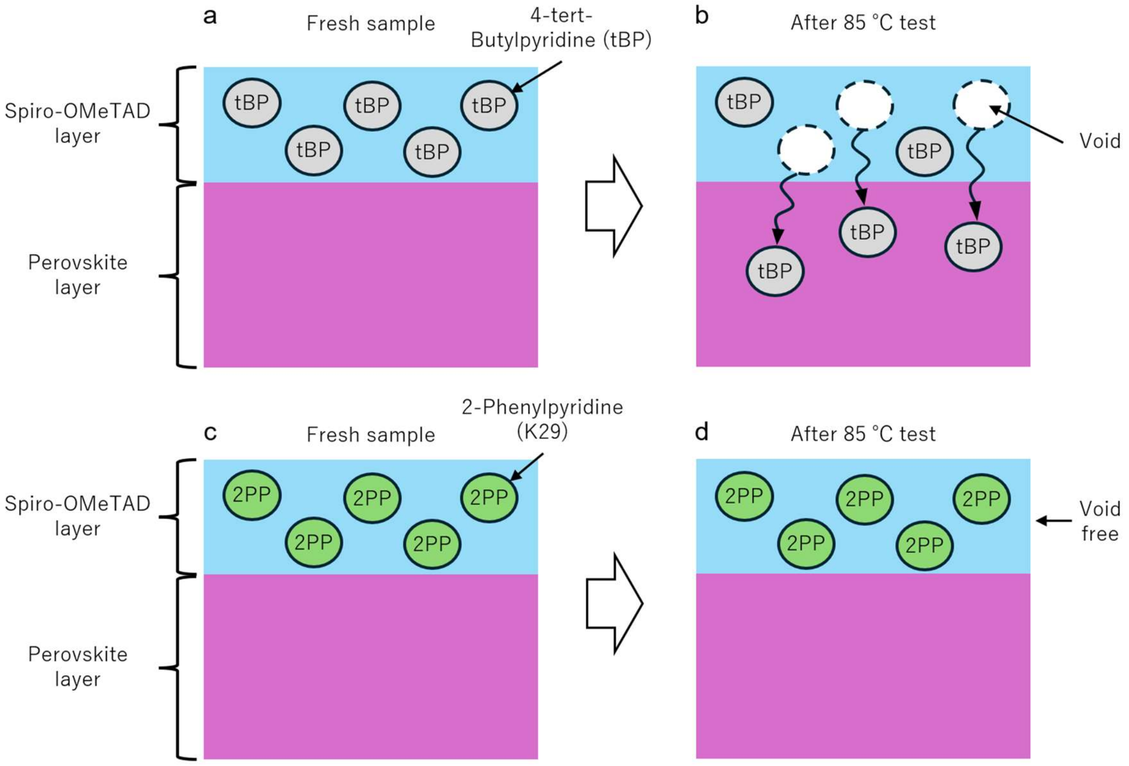

Supplementary Figure 81. Schematics images of void formation and void-free by additives. (a) a fresh device with 4-tert-butylpyridine, (b) the same device after the 85 °C test, (c) a fresh device with 2-phenylpyridine, and (d) the same device after the 85 °C test.

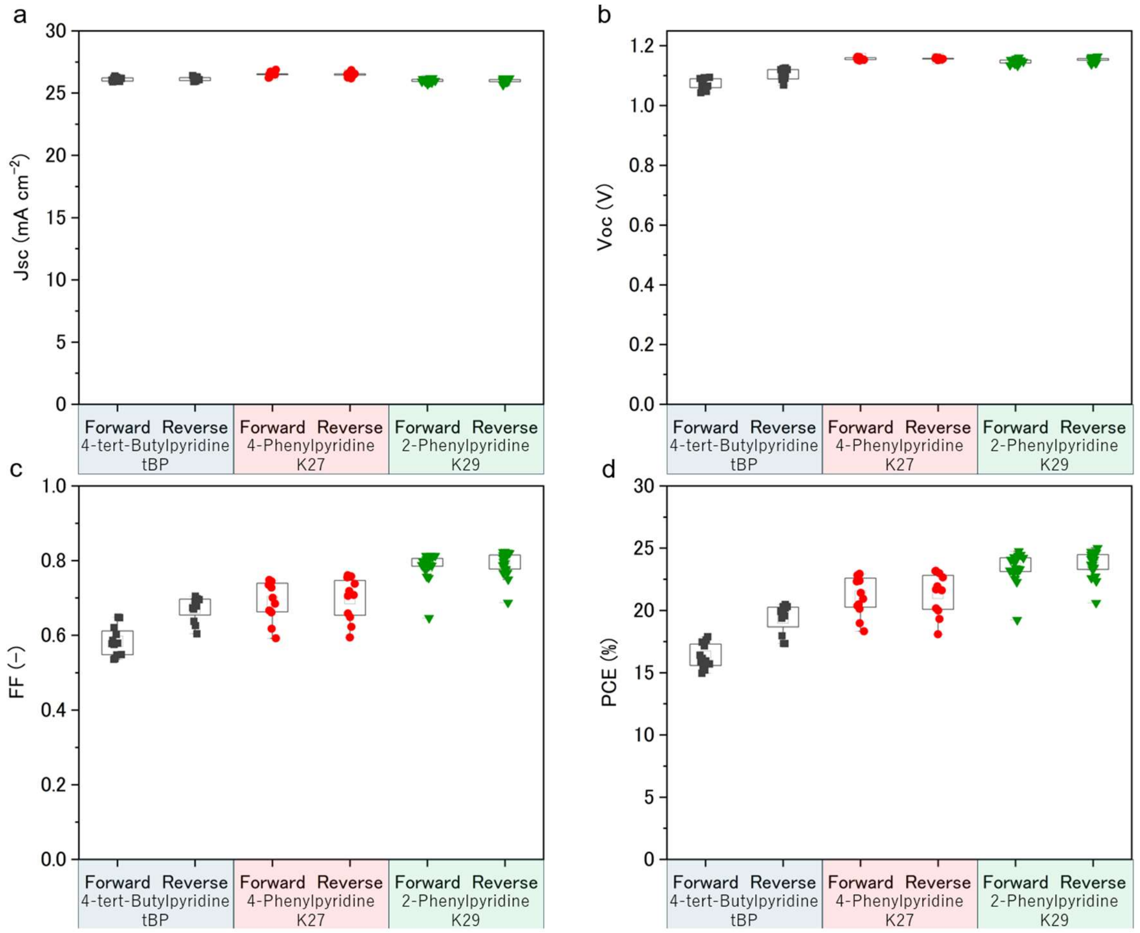

Supplementary Figure 82. Statics of photovoltaic parameters of perovskite solar cells with 4-*tert*-butylpyridine (Li+tBP), 4-phenylpyridine (Li+K27), and 2-phenylpyridine (Li+K29) for (a) Jsc, (b) Voc, (c) FF, and (e) PCE. Total number of samples was 50. MACl concentration were 10%.

Supplementary Table 63. Photovoltaic parameters for perovskite solar cells with tBP. MACl concentration increased from 10% to 20% to improve efficiency.

Cell	Jsc (mA/sq)	Jsc (mA/sq)	Voc (V)	Voc (V)	FF (-)	FF (-)	PCE (%)	PCE (%)
	Forward	Reverse	Forward	Reverse	Forward	Reverse	Forward	Reverse
1	26.17	26.17	1.058	1.085	0.549	0.726	15.21	20.63
2	26.13	26.13	1.087	1.113	0.612	0.771	17.37	22.43
3	26.20	26.19	1.103	1.123	0.541	0.717	15.66	21.11
4	26.21	26.21	1.107	1.125	0.455	0.707	13.20	20.86

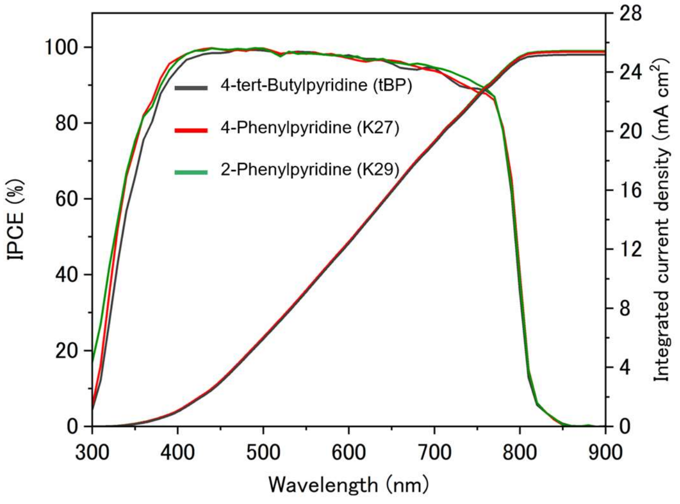

Supplementary Figure 83. IPCE of perovskite solar cells with 4-*tert*-butylpyridine (tBP), 4-phenylpyridine (K27), and 2-phenylpyridine (K29). The J_{SC} obtained from the J-V characteristics was found to be consistent with the value integrated from the external quantum efficiency (EQE) spectrum, with a relative difference of less than 4%.

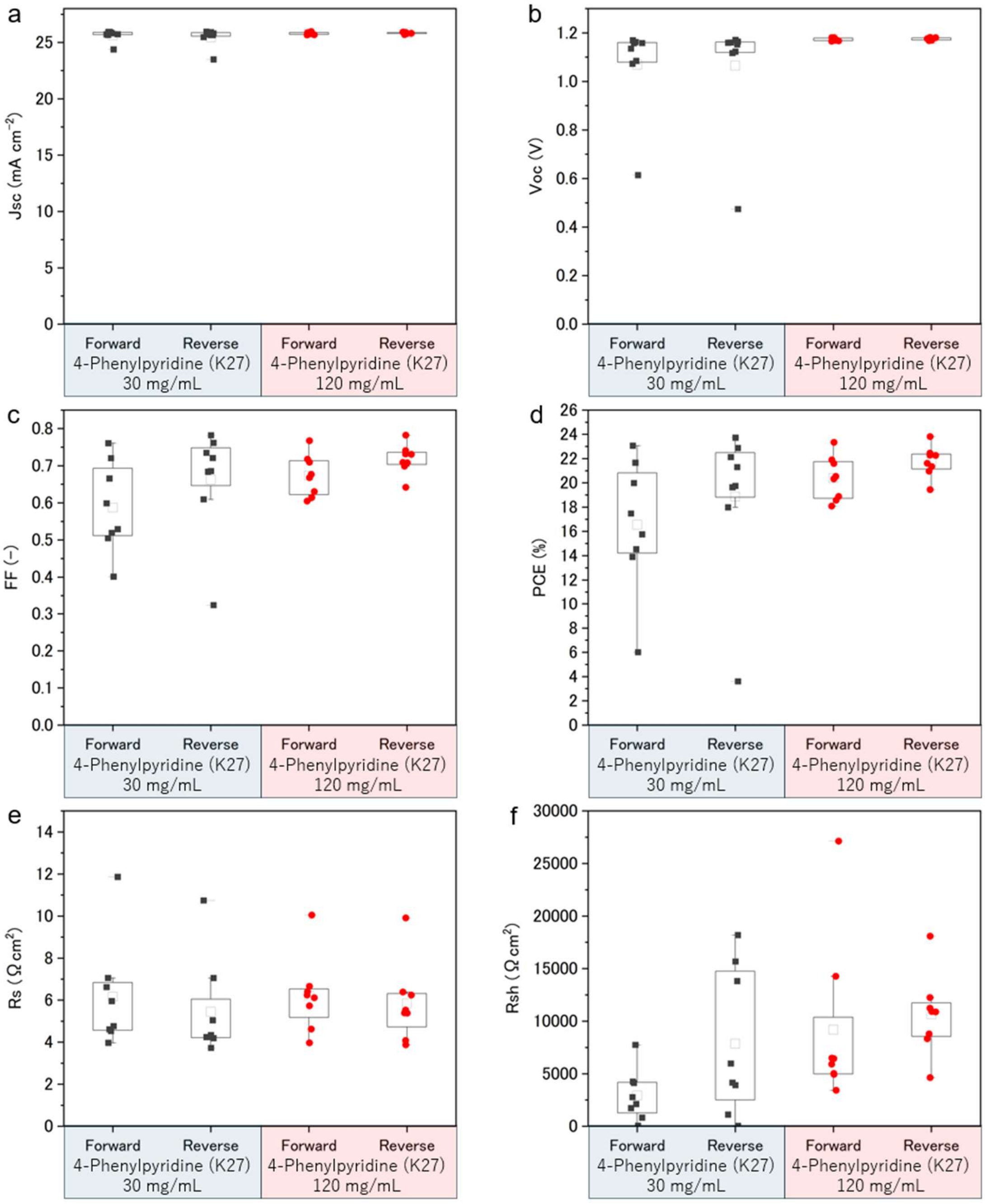

Supplementary Figure 84. Statics of photovoltaic performance of perovskite solar cells comparing 30 mg/mL and 120 mg/mL of 4-phenylpyridine in Spiro-OMeTAD solution for (a) Jsc, (b) Voc, (c), FF, (d) PCE, (e) Rs, and (f) Rsh. Total number of samples was 16.

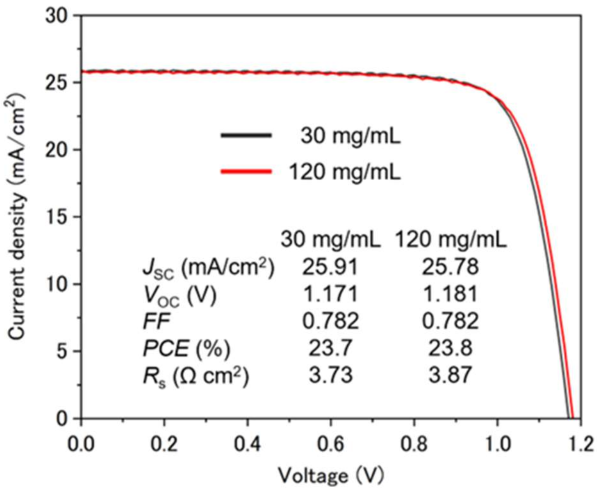

Supplementary Figure 85. IV-curves of champion solar cells comparing 30 mg/mL and 120 mg/mL of Spiro-OMeTAD solution containing 4-phenylpyridine and LiTFSI.

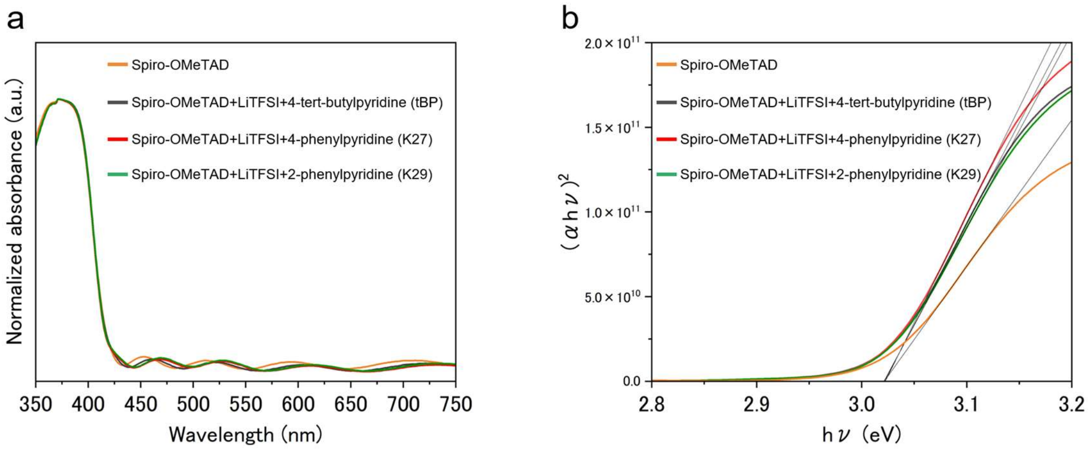

Supplementary Figure 86. (a) UV-vis spectra and (b) bandgap of Spiro-OMeTAD with additives. Bandgap was 3.02 eV.

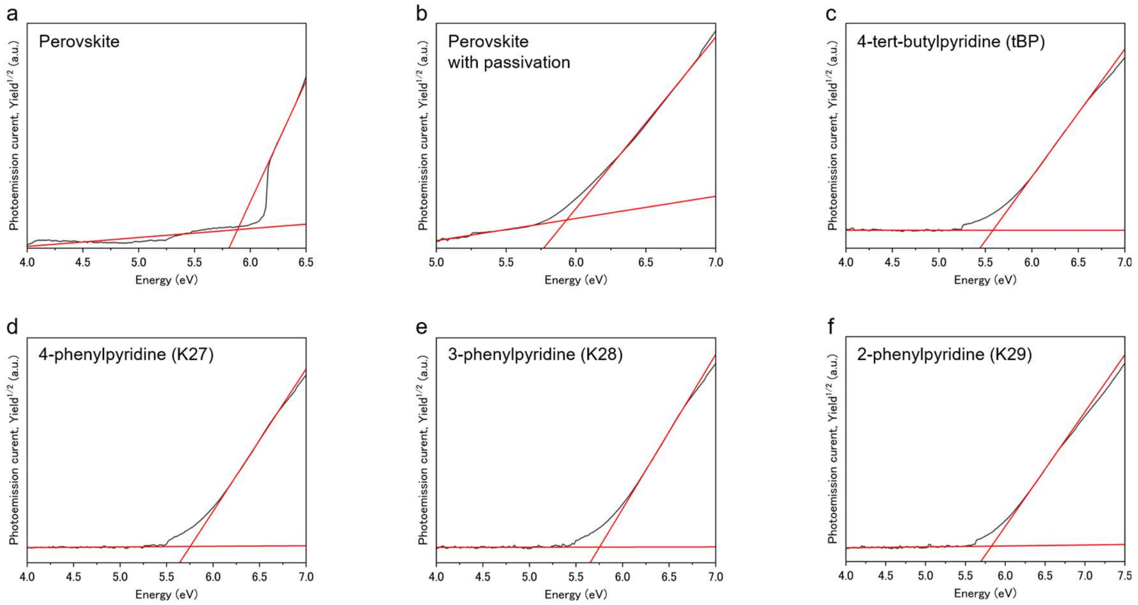

Supplementary Figure 87. Photoelectron yield spectroscopy of (a) perovskite, (b) perovskite with OAI passivation, (c) spiro-OMeTAD with LiTFSI and 4-tert-butylpyridine, (d) spiro-OMeTAD with LiTFSI and 4-phenylpyridine, (e) spiro-OMeTAD with LiTFSI and 3-phenylpyridine, (f) spiro-OMeTAD with LiTFSI and 2-phenylpyridine.

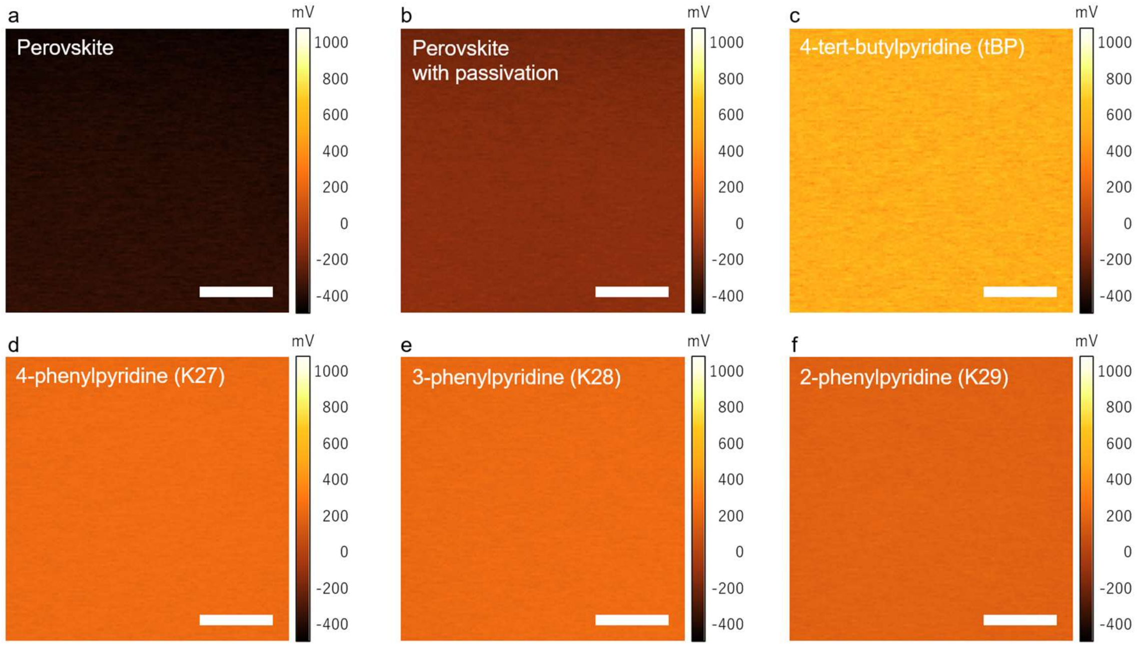

Supplementary Figure 88. KPFM mapping of (a) perovskite, (b) perovskite with OAI passivation, (c) spiro-OMeTAD with LiTFSI and 4-tert-butylpyridine, (d) spiro-OMeTAD with LiTFSI and 4-phenylpyridine, (e) spiro-OMeTAD with LiTFSI and 3-phenylpyridine, (f) spiro-OMeTAD with LiTFSI and 2-phenylpyridine. Scale bar corresponds to 5 μm.

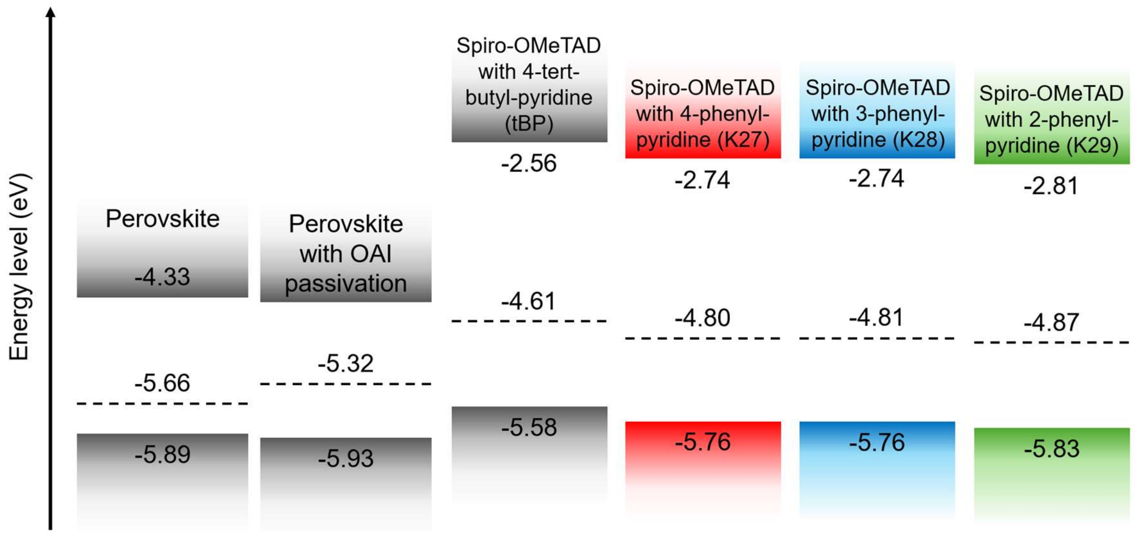

Supplementary Figure 89. Energy diagram of perovskite and spiro-OMeTAD with additives. Energy diagrams were calculated with photoelectron yield spectroscopy, KPFM, and bandgap from UVVIS spectroscopy. Bandgap of perovskite and phenylpyridine was associated with 1.56 eV and 3.02 eV, respectively. Spiro-OMeTAD include LiTFSI and each additive same as recipe with device fabrication.

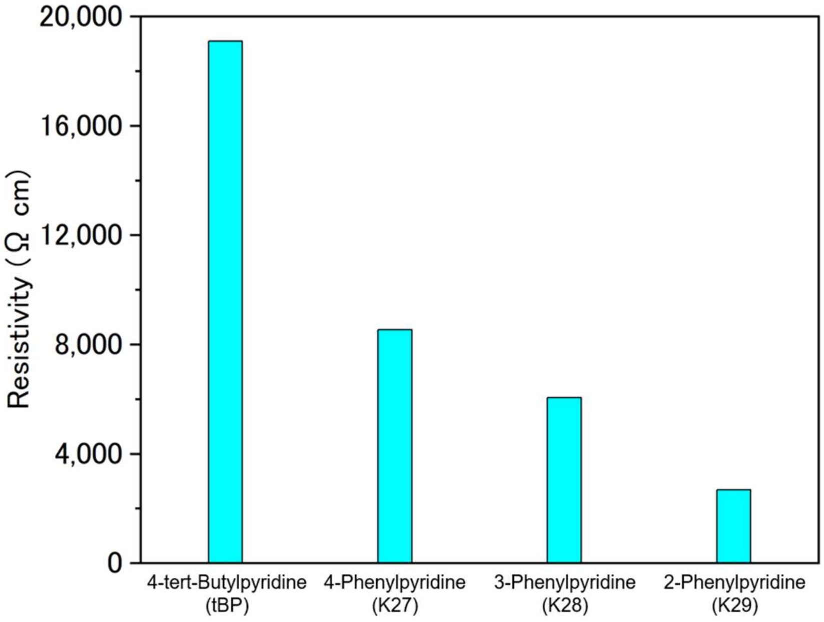

Supplementary Figure 90. The resistivity of Spiro-OMeTAD layers containing LiTFSI and each additive was measured using the van der Pauw method. The Spiro-OMeTAD layers were deposited on glass substrates by spin-coating. Spiro-OMeTAD solutions were prepared using the same procedure as in the device fabrication.

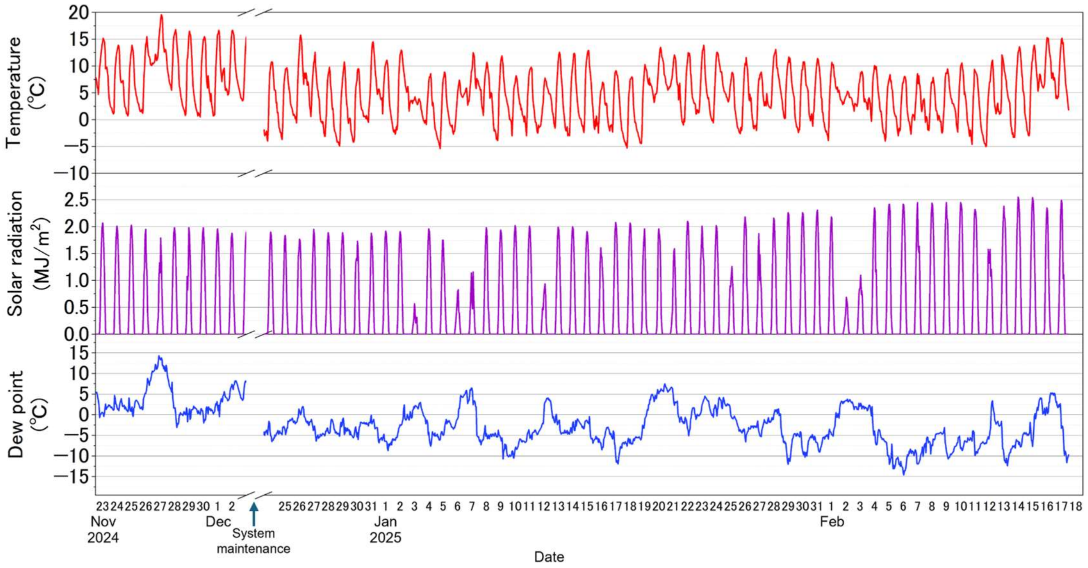

Supplementary Figure 91. Weather data of temperature, solar radiation, and dew point at Tsukuba Tateno, Ibaraki, Japan. Data was processed by using the weather data from Japan Meteorological Agency.[1]

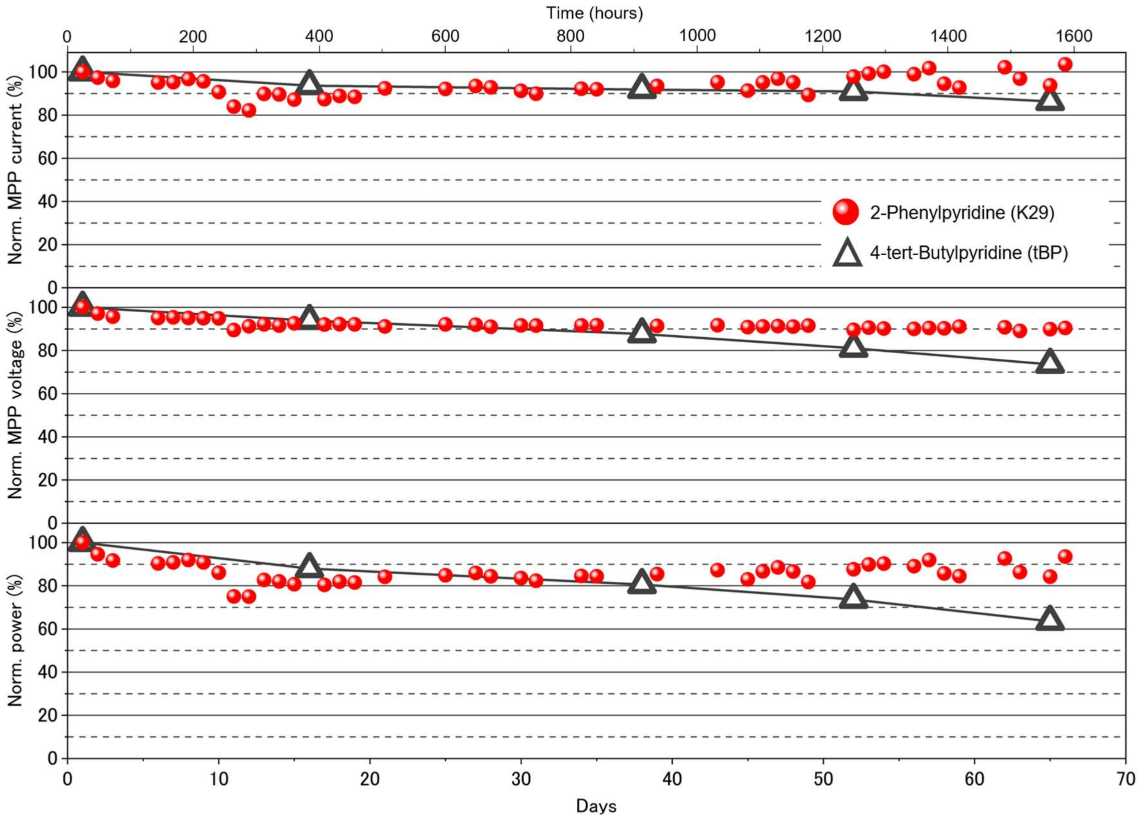

Supplementary Figure 92. Outdoor stability results of perovskite solar cells. Red circles and gray triangles are devices with 2-phenylpyridine (Li+K29) and tBP (Co,Li+tBP), respectively. Note that initial device performance with 2-phenylpyridine was *J*sc=23.69 mA/sq, *V*oc=1.089 V, *FF*=0.637, and *PCE*=16.4%. And tBP was *J*sc=23.17 mA/sq, *V*oc=0.973 V, *FF*=0.714, and *PCE*=16.1%. Average (11:30-12:30) of maximum power point current, maximum power point voltage, and maximum power were plotted for devices with 2-phenylpyridine. The aberrant values due to clouds have been removed. Devices with tBP were kept outdoors with a fixed resistance method to be maximum-power point and measured performance with solar simulator (100 mW/cm^2) from time to time starting from 18th of October 2023.

Supplementary References

1 Japan Meteorological Agency, URL: https://www.jma.go.jp/jma/index.html
