# Supplementary material for: Heteroaryl derivatives for hole-transport layers improve thermal stability of perovskite solar cells
Source: Nat Commun. 2026 Feb 13;17:1664. doi: 10.1038/s41467-025-68236-9 (PMC12909905; doi:10.1038/s41467-025-68236-9)
Supplement: Supplementary file 2 — Reporting Summary [file 41467_2025_68236_MOESM2_ESM.pdf]

nature portfolio

Corresponding author(s): *Double-anonymous peer review submissions: write DAPR and your manuscript number here instead of author names.*

Solar Cells Reporting Summary

Nature Portfolio wishes to improve the reproducibility of the work that we publish. This form is intended for publication with all accepted papers reporting the characterization of photovoltaic devices and provides structure for consistency and transparency in reporting. Some list items might not apply to an individual manuscript, but all fields must be completed for clarity.

For further information on Nature Research policies, including our data availability policy, see Authors & Referees.

• Experimental design

Please check the following details are reported in the manuscript, and provide a brief description or explanation where applicable.

1. Dimensions

Area of the tested solar cells

- [x] Yes
- [ ] No

The aperture area for the solar cells was 0.0887 cm2. The active area for mini modules was 1.44 cm2. (Aperture area was 1.664cm2) The active are for outdoor devices were 0.25 cm2. (Methods)

Explain why this information is not reported/not relevant.

Method used to determine the device area

- [x] Yes
- [ ] No

The aperture area of the solar cells was defined by an opaque metal mask with square aperture of area of 0.0887 cm2. The active and aperture area for mini module was determined by the active area excluding or including P1,P2,P3 area, respectively. The device area for outdoor devices were determined by the active area. (Methods)

Explain why this information is not reported/not relevant.

2. Current-voltage characterization

Current density-voltage (J-V) plots in both forward and backward direction

- [x] Yes
- [ ] No

See Figure 5

Voltage scan conditions

- [x] Yes
- [ ] No

The scanning step and speed for solar cells were 10 mV and 100 mV s-1, respectively. The scanning step and speed for mini modules were 50 mV and 500 mV s-1, respectively. (Methods)

Explain why this information is not reported/not relevant.

Test environment

- [x] Yes
- [ ] No

IV curves of devices with or without encapsulation were measured at room temperature in air. (Methods)

Explain why this information is not reported/not relevant.

Protocol for preconditioning of the device before its characterization

- [x] Yes
- [ ] No

No preconditioning of the device was applied before the measurement

Explain why this information is not reported/not relevant.

Stability of the J-V characteristic

- [x] Yes
- [ ] No

See Figure 2 and supplementary information

Explain why this information is not reported/not relevant.

3. Hysteresis or any other unusual behaviour

Description of the unusual behaviour observed during the characterization

- [x] Yes
- [ ] No

The devices showed some hysteresis in case of reference cells and negligible hysteresis behaviors in case of target cells. (Main text, Photovoltaic performances with heteroaryl derivatives part)

Explain why this information is not reported/not relevant.

Related experimental data

- [x] Yes
- [ ] No

See Figure 5

Explain why this information is not reported/not relevant.

4. Efficiency

External quantum efficiency (EQE) or incident photons to current efficiency (IPCE)

- [x] Yes
- [ ] No

See Supplementary Fig. S69, S83.

Explain why this information is not reported/not relevant.

A comparison between the integrated response under the standard reference spectrum and the response measure under the simulator

- [x] Yes
- [ ] No

The JSC obtained from the J-V characteristics was found to be consistent with the value integrated from the external quantum efficiency (EQE) spectrum, with a relative difference of less than 4%. (Supplementary Fig. 83).

Explain why this information is not reported/not relevant.

For tandem solar cells, the bias illumination and bias voltage used for each subcell

- [ ] Yes
- [x] No

Provide a description of the measurement conditions.

No tandem solar cell was reported in this manuscript.

5. Calibration

Light source and reference cell or sensor used for the characterization

- [x] Yes
- [ ] No

AM1.5 pseudo-solar light (100 mW cm−2) was generated using a solar simulator equipped with a xenon lamp (Bunkou Keiki). Standard silicon solar cells is from Bunkou Keiki. (Methods)

Explain why this information is not reported/not relevant.

Confirmation that the reference cell was calibrated and certified

- [x] Yes
- [ ] No

Standard silicon solar cells (BS-521BK, Bunkou Keiki) were used to calibrate the incident light intensity. (Methods)

Explain why this information is not reported/not relevant.

Calculation of spectral mismatch between the reference cell and the devices under test

- [ ] Yes
- [x] No

Provide a value of the spectral mismatch and/or a description of how it has been taken into account in the measurements.

No spectral mismatch calculation was performed.

6. Mask/aperture

Size of the mask/aperture used during testing

- [x] Yes
- [ ] No

The aperture area for the solar cells was 0.0887 cm2. The active area for mini modules was 1.44 cm2. (Aperture area was 1.664cm2). Mini module and device for outdoor were measured without mask. (Methods)

Explain why this information is not reported/not relevant.

Variation of the measured short-circuit current density with the mask/aperture area

- [ ] Yes
- [x] No

Report the difference in the short-circuit current density values measured with the mask and aperture area.

We didn't measure the solar cells with apertures of different sizes.

7. Performance certification

Identity of the independent certification laboratory that confirmed the photovoltaic performance

- [ ] Yes
- [x] No

Identify the independent certification laboratory.

We did not obtain certification for devices since this study forces on stability.

A copy of any certificate(s)

- [ ] Yes
- [x] No

Certificate copies should be provided in the Supplementary information. Please state the supplementary item number.

We did not obtain certification for devices since this study forces on stability.

8. Statistics

Number of solar cells tested

- [x] Yes
- [ ] No

Totally 50. (Supplementary Fig. S82)

Explain why this information is not reported/not relevant.

Statistical analysis of the device performance

- [x] Yes
- [ ] No

See Supplementary Fig. S82

Explain why this information is not reported/not relevant.

9. Long-term stability analysis

Type of analysis, bias conditions and environmental conditions

- [x] Yes
- [ ] No

Thermal stability (85℃, in air, in dark, without bias) were performed for encapsulated devices. IV curves were measured periodically. (Methods, Figure 2)
Outdoor stability test were performed for encapsulated devices with MPPT. (Methods, Figure 5)

Explain why this information is not reported/not relevant.
